# High‐Throughput Screening Reveals That CeeNU Acts as a New NLRP3 Inflammasome Inhibitor

**DOI:** 10.1002/mco2.70695

**Published:** 2026-04-23

**Authors:** Sen‐Lin Ji, Peipei Chen, Huaiping Tang, Chao Zhou, Zihao Li, Yunshu Wang, Xiang Cao, Liwen Zhu, Xinyu Bao, Zhuo Liu, Yan Chen, Yun Xu

**Affiliations:** ^1^ Department of Neurology Nanjing Drum Tower Hospital Affiliated Hospital of Medical School Nanjing University Nanjing China; ^2^ State Key Laboratory of Pharmaceutical Biotechnology and Institute of Translational Medicine for Brain Critical Diseases Nanjing University Nanjing China; ^3^ Jiangsu Key Laboratory for Molecular Medicine Medical School of Nanjing University Nanjing China; ^4^ Nanjing Neurology Clinical Medical Center Nanjing China

**Keywords:** CeeNU, experimental autoimmune encephalomyelitis, inhibitor, macrophages, NLRP3

## Abstract

Pyroptosis is a special form of cell death that often occurs during excessive inflammation and injury, leading to tissue damage, disease progression, and other related issues. The Nod‐like receptor family pyrin domain‐containing 3 (NLRP3) inflammasome is an important regulatory factor in cellular pyroptosis that promotes the inflammatory response. Inhibitors targeting the NLRP3 inflammasome have emerged as promising potential therapeutic agents for inflammatory diseases. Through large‐scale screening, we found that the FDA‐approved drug CeeNU strongly inhibited NLRP3‐mediated pyroptosis. CeeNU exhibited dose‐dependent suppression of NLRP3 inflammasome activation and effectively mitigated inflammasome‐driven pyroptotic cell death in both human and murine macrophages/microglia. Mechanistically, we further demonstrated that CeeNU specifically binds to arginine 335 within the NACHT domain of NLRP3, abrogating NLRP3 inflammasome activation by blocking its assembly. Importantly, CeeNU showed remarkable protective effects in multiple mouse models of NLRP3 inflammasome‐mediated diseases, including experimental autoimmune encephalomyelitis (EAE) induced by myelin oligodendrocyte glycoprotein (MOG), lipopolysaccharide (LPS)‐induced septic shock, monosodium urate (MSU)‐induced peritonitis, and MSU‐induced gouty arthritis. Our results demonstrate that CeeNU, a clinically available drug, acts as an NLRP3 inhibitor and holds therapeutic potential for NLRP3 inflammasome‐mediated pyroptotic diseases.

## Graphical abstract

We conducted a high‐throughput screen of 2747 FDA‐approved drugs to identify inhibitors of pyroptosis. We found that CeeNU significantly repressed NLRP3‐dependent IL‐1β release and GSDMD pore‐forming complex. We identified that the arginine 335 amino acid of the NACHT domain of NLRP3 is the critical point for human NLRP3 and CeeNU interaction and preventing NLRP3 inflammasome activation. Collectively, CeeNU is a specific and highly effective NLRP3 inhibitor with promising application for the treatment of NLRP3‐driven diseases.

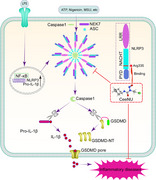

## 1 Introduction

The Nod‐like receptor family pyrin domain‐containing 3 (NLRP3)‐mediated pyroptosis is a unique form of cell death that differs from other common cell death modes, such as apoptosis and necrosis; it is considered a highly regulated and reversible process of cell death with important physiological and pathophysiological significance [1, 2]. During pyroptosis, cells are damaged or stimulated through multiple signaling pathways, resulting in a series of changes inside the cells, including organelle imbalance, abnormal mitochondrial function, and oxidative stress [3, 4]. These events lead to cell membrane rupture, as well as the massive leakage of cytosolic contents, including damage‐associated molecular patterns and mature interleukin‐1 beta (IL‐1β), triggering strong inflammation and rapid activation of the immune system [5, 6, 7]. The NLRP3 inflammasome is an intracellular multiprotein complex that critically regulates inflammatory and immune responses [7, 8]. NLRP3 inflammasome activation represents a key mechanism underlying inflammatory processes [9]. When cells are damaged, infected, or otherwise stimulated, the activated NLRP3 receptor promotes ASC aggregation and the recruitment of procaspase‐1 to the inflammasome. Subsequently, procaspase‐1 is cleaved to activate caspase‐1, thus triggering a series of inflammatory responses [10]. Activation of caspase‐1 stimulates the generation of pro‐inflammatory mediators, including IL‐1β [11]. Caspase‐1 mediates the proteolytic cleavage of gasdermin D (GSDMD), resulting in the formation of its N‐terminal fragment (GSDMD‐NT). This fragment subsequently oligomerizes to create pores in the plasma membrane, thereby inducing pyroptotic cell death and facilitating the release of intracellular components such as IL‐1β and lactate dehydrogenase (LDH) [12, 13]. These inflammatory mediators significantly contribute to both inflammatory processes and immune regulation, and are implicated in the pathogenesis of a range of disorders, such as multiple sclerosis (MS) [14, 15], sepsis [16], peritonitis [17, 18], and gouty arthritis [19, 20]. Therefore, identification of a well‐tolerated drug specifically blocking NLRP3 inflammasome is still of extreme emergency in the clinic.

Abnormal NLRP3 inflammasome activation is closely related to the inflammatory response and may play an important role in the development of various diseases [21]. The NLRP3 inflammasome has emerged as a highly promising pharmacological target for treating inflammatory diseases, owing to its potent inflammatory effects and key involvement in disease mechanisms [22, 23, 24].

In recent years, several NLRP3‐targeted inhibitors have been developed and applied in animal models or clinical studies. As an NLRP3 inhibitor, RRx‐001 treatment alleviates sepsis, colitis, and experimental autoimmune encephalomyelitis (EAE) in mice [25]. It is encouraging that RRx‐001, currently the most clinically advanced NLRP3inflammasome inhibitor, has undergone safety evaluations in over 300 treatment‐resistant cancer patients with comorbidities across 12 clinical trials [26]. The inhibitory effect of oridonin on the NLRP3 inflammasome is not achieved through traditional upstream signal interference but rather via direct covalent binding to the NLRP3 protein itself, thereby precisely locking its activation at the molecular level. In various mouse disease models associated with NLRP3 overactivation, oridonin has demonstrated remarkable therapeutic efficacy [19]. Dehydrocostus lactone (DCL), a natural product, ameliorates disease progression of Crohn's disease, septic shock, and peritonitis by covalently and irreversibly targeting NLRP3 [27].

Although a number of selective NLRP3 inflammasome inhibitors have demonstrated robust efficacy in the treatment of NLRP3‐mediated pathologies [26, 28], none of these inhibitors is clinically available due to their toxicity and lack of efficacy. Therefore, identifying candidate NLRP3 inflammasome‐specific inhibitors from FDA‐approved drug libraries remains an urgent requirement for the management of NLRP3‐associated pathologies.

CeeNU (also named CCNU, Lomustine) is an oral agent used for the treatment of recurrent or progressive glioblastoma multiforme [29]; it is effective in primary brain tumor chemotherapy due to its capacity to cross the blood‐brain barrier and can also be used to treat Hodgkin's disease and lymphoma [30]. The combined use of CeeNU and other drugs has been shown to improve the survival rate of patients [31]. CeeNU therapy significantly reduces the synthesis of central nervous system (CNS) IgG in patients with MS, indicating that CeeNU has an immunosuppressive effect [32]. CeeNU has demonstrated efficacy in clinical applications; however, its anti‐inflammatory mechanisms and specific molecular targets have yet to be fully elucidated.

In this study, we identified CeeNU as a potential and effective NLRP3 inflammasome inhibitor from a bank of 2747 FDA‐approved drugs that functions by directly binding to NLRP3 at arginine 335 (R335). Furthermore, CeeNU conferred protection in murine models of NLRP3 inflammasome‐associated pathologies, including myelin oligodendrocyte glycoprotein (MOG)‐induced EAE, lipopolysaccharide (LPS)‐induced septic shock, monosodium urate (MSU)‐induced peritonitis, and MSU‐induced gouty arthritis. Our results demonstrate that the clinically available agent CeeNU functions as a selective NLRP3 inhibitor, representing a promising therapeutic candidate for treating NLRP3 inflammasome‐mediated pyroptotic disorders.

## 2 Results

### 2.1 High‐Throughput Screening Revealed That CeeNU Is an Effective NLRP3 Inhibitor

**FIGURE 1:**
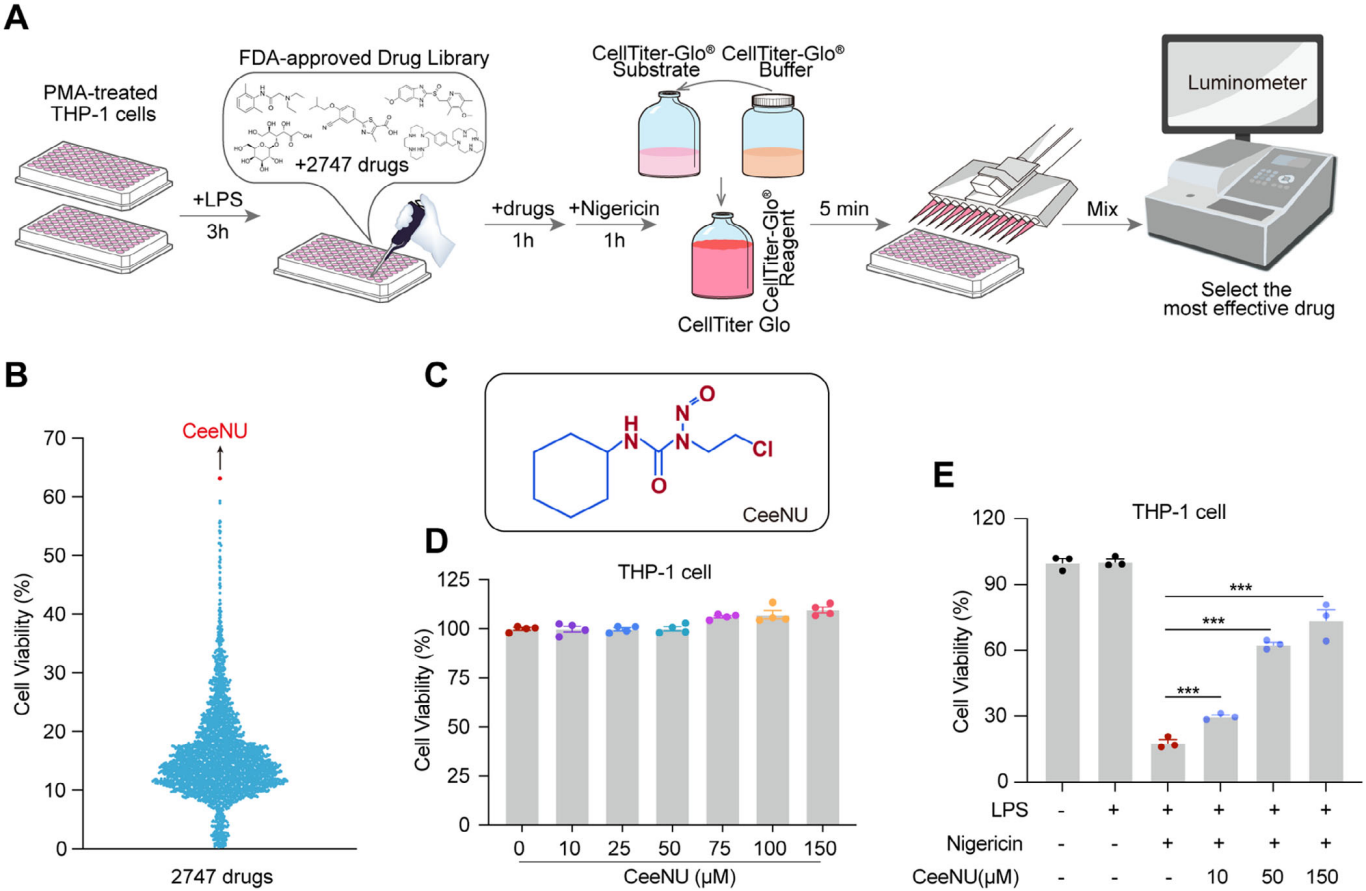
High‐throughput screening identifies CeeNU as an effective NLRP3 inhibitor. (A and B) The process of selecting the most effective drug CeeNU from the FDA‐approved Drug Library by CellTiter‐Glo. (C) The chemical structure of CeeNU. (D) THP‐1 cells were exposed to graded concentrations of CeeNU for 24 h, and cell viability was subsequently determined by the CCK‐8 assay. (E) LPS‐primed THP‐1 cells were treated with CeeNU for 1 h, stimulated with nigericin for an additional hour, and cell viability was then measured using the CellTiter‐Glo Luminescent Assay. ^***^ *p* < 0.001. Data are mean ± SEM.

The NLRP3 inflammasome plays a critical role in the regulation of the innate immune response. The inflammasome serves as the primary detector of sterile inflammatory signals, making it a crucial initiator of inflammatory responses. Various cellular and molecular processes, such as hypoxia, glutamate release, elevated reactive oxygen species (ROS) levels, impaired mitochondrial function, ion imbalance, neuronal demyelination, and cell death, contribute to inflammasome activation [33]. To identify the clinical drugs that exert anti‐NLRP3 inflammasome‐related pyroptosis effects, the CellTiter‐Glo Assay was employed with cells cultured in 96‐well plates due to its high compatibility with automated high‐throughput screening platforms for cell proliferation and cytotoxicity assessment. A CellTiter‐Glo luminescent cell viability assay was used to determine ATP‐based cell viability during pyroptosis [34]. The effects of the FDA‐approved Drug Library on the activation of NLRP3 inflammasome‐related pyroptosis were tested by quantifying the amount of ATP present, which indicates the presence of metabolically active cells (Figure 1A). Among 2747 clinically approved compounds screened (Table S1), CeeNU demonstrated the most potent suppression of LPS/nigericin‐induced NLRP3 inflammasome‐mediated cytotoxicity in phorbol 12‐myristate‐13‐acetate (PMA)‐differentiated THP‐1 macrophages (Figure 1B,C). To determine the optimal dose of CeeNU required for the effective inhibition of NLRP3 inflammasome activation, the cytotoxicity of CeeNU in THP‐1 cells was tested. Cell viability assessment revealed that CeeNU displayed no cytotoxic effects at concentrations up to 150 µM in differentiated human THP‐1 macrophages (Figure 1D). CeeNU profoundly prevented cell cytotoxicity in a dose‐dependent manner in LPS‐primed THP‐1 cells stimulated with nigericin (Figure 1E).

### 2.2 CeeNU Strongly Represses Pyroptosis and IL‐1β Release in an NLRP3‐Dependent Manner in Human Macrophages

**FIGURE 2:**
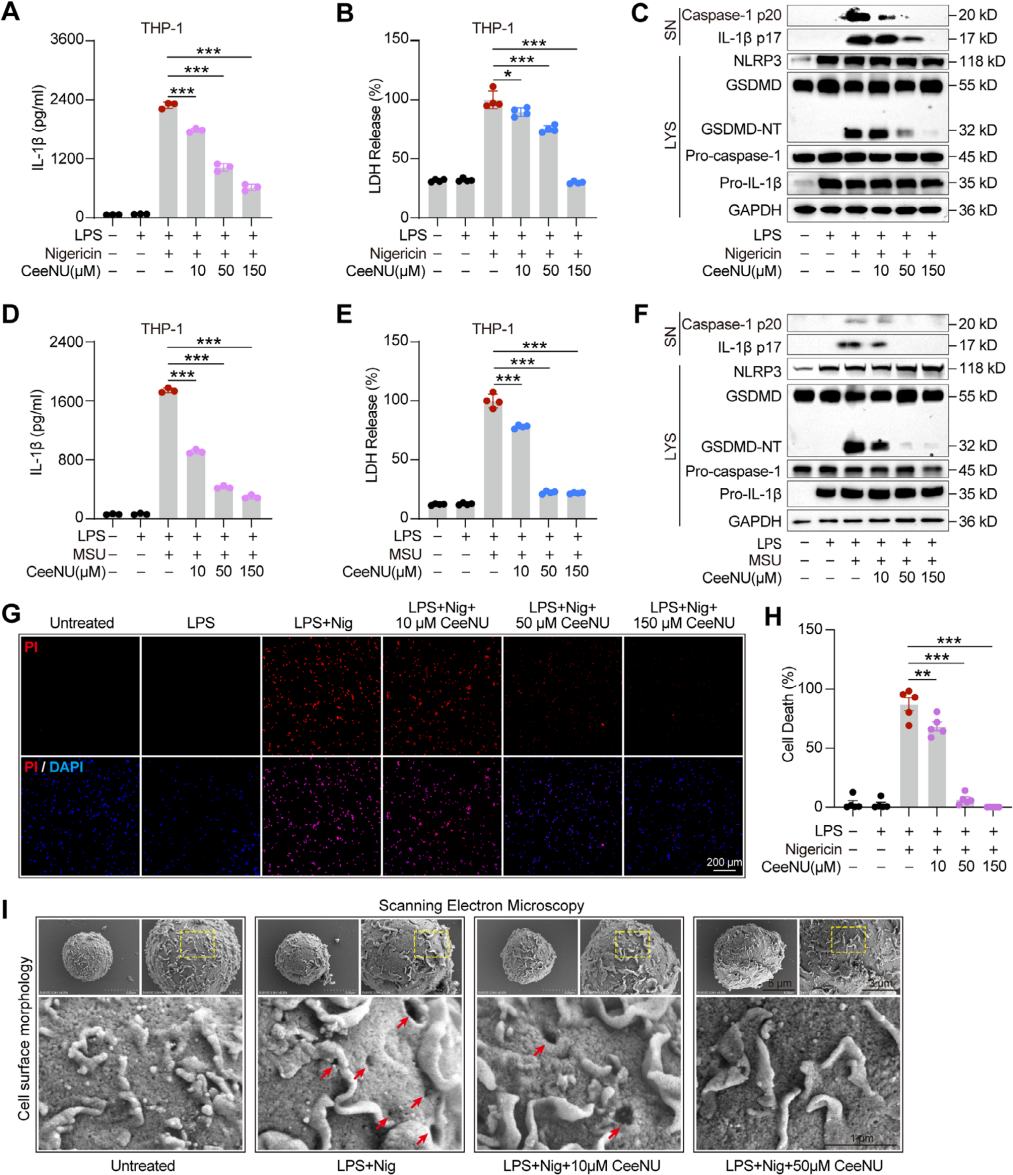
CeeNU remarkably represses pyroptosis and IL‐1β release dependent on NLRP3 in human macrophages. (A, B, D, and E) PMA‐differentiated human macrophage THP‐1 cells were either treated with LPS for 3 h plus CeeNU for 1 h, followed by stimulation with nigericin (A and B) or MSU (D and E), the supernatant was collected for ELISA of IL‐1β (A and D) and LDH assay (B and E). (C and F) Western blotting analysis of cleaved IL‐1β p17 and caspase‐1 p20 levels in cell supernatant and pro‐IL‐1β, procaspase‐1, NLRP3, GSDMD, GSDMD‐NT, and GAPDH in cell lysates of PMA‐differentiated THP‐1 cells primed with LPS for 4 h in the presence of CeeNU or control, prior to stimulation with nigericin (C) or MSU(F). (G) PMA‐differentiated THP‐1 cells were treated sequentially: first with LPS for 3 h, then with CeeNU for an additional hour. Following this, cells were stimulated with nigericin for 1 h. Subsequently, cells were stained with propidium iodide (PI, 2 µg/mL) to label dying cells and DAPI (1 µg/mL) to label all nuclei for 15 min. Imaging was performed using a 10× objective on an Olympus confocal microscope. Merged images include the corresponding bright‐field view. (H) The number of PI‐positive cells was counted in five randomly chosen microscopic fields. Lytic cell death was defined as the ratio of PI‐positive cells to the total number of DAPI‐positive nuclei and is expressed as a percentage. (I) Scanning electron microscopy of cell surface perforations caused by GSDMD in THP‐1 (Bar = 3 µm). ^*^ *p* < 0.05, ^**^ *p* < 0.01, ^***^ *p* < 0.001. Data are mean ± SEM.

First, specific cell types were exposed to a range of CeeNU concentrations, which were meticulously selected based on preliminary assays to ensure they remained within a non‐cytotoxic range (Figure S1). To further determine whether CeeNU inhibits NLRP3 inflammasome activation, we evaluated its impact on IL‐1β secretion in PMA‐differentiated human THP‐1 macrophages. The results showed that CeeNU reduced IL‐1β secretion and LDH release in a dose‐dependent manner when triggered by nigericin or MSU in LPS‐primed THP‐1 cells (Figure 2A,B,D,E). In addition, CeeNU dose‐dependently inhibited the cleavage of IL‐1β p17 and caspase‐1 p20, which are hallmarks of NLRP3 inflammasome activation, but had no impact on pro‐IL‐1β, procaspase‐1, or NLRP3 levels in cell lysates (Figure 2C,F). Moreover, CeeNU inhibited GSDMD activation, and PI uptake (Figure 2G,H). The induction of cellular pyroptosis is primarily mediated by inflammasome‐dependent activation of caspase family proteins. This activation triggers proteolytic cleavage of GSDMD proteins, generating fragments that translocate to the plasma membrane. Subsequent oligomerization of these fragments forms membrane pores, resulting in osmotic imbalance, cytoplasmic content release, and eventual lytic cell death characteristic of pyroptosis. To observe the changes in the cell membrane after treatment with LPS/nigericin, scanning electron microscopy was used. As shown in Figure 2I, GSDMD membrane pores, cell swelling, plasma membrane rupture, and pyroptosis were observed; however, CeeNU significantly mitigated LPS/nigericin‐induced cell rupture and pore formation. Collectively, these findings demonstrate that CeeNU specifically suppresses NLRP3 inflammasome activation and attenuates NLRP3‐driven pyroptotic cell death.

### 2.3 CeeNU Inhibits Pyroptotic Cell Death in Mouse Macrophages

**FIGURE 3:**
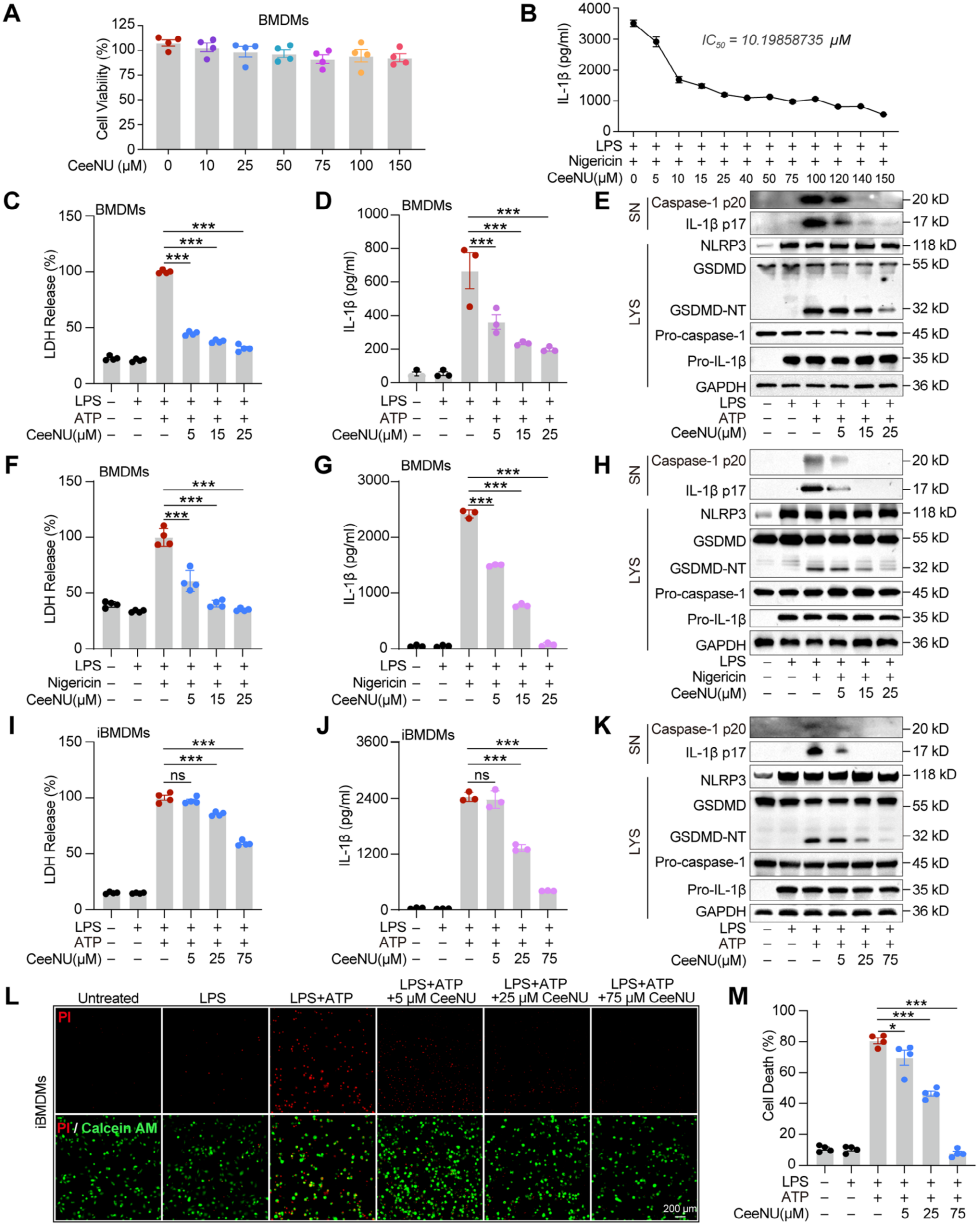
CeeNU inhibits pyroptotic cell death in mouse macrophages. LPS‐primed BMDM or iBMDMs treated with CeeNU for 1 h before stimulation with ATP for 30 min or nigericin for 1 h. (A) BMDMs were treated with different doses of CeeNU for 24 h, and cell viability was assessed by the CCK‐8 assay. (B) ELISA of IL‐1β in the supernatant of LPS+Nigericin‐stimulated BMDMs. (C and F) LDH release from BMDMs supernatant stimulated with LPS+ATP (C), or LPS+Nigericin (F). (D and G) ELISA of IL‐1β in the supernatant of BMDMs stimulated with LPS+ATP (D), or LPS+Nigericin (G). (E and H) Western blotting analysis of cleaved IL‐1β p17 and caspase‐1 p20 levels in cell supernatant and pro‐IL‐1β, procaspase‐1, NLRP3, GSDMD, GSDMD‐NT, and GAPDH in cell lysates of BMDMs stimulated with LPS+ATP (E), or LPS+Nigericin (H). (I) LDH release from BMDMs supernatant stimulated with LPS+ATP. (J) ELISA of IL‐1β in the supernatant of BMDMs stimulated with LPS+ATP. (K) Western blotting analysis of cleaved IL‐1β p17 and caspase‐1 p20 levels in cell supernatant and pro‐IL‐1β, procaspase‐1, NLRP3, GSDMD, GSDMD‐NT, and GAPDH in cell lysates of iBMDMs stimulated with LPS+ATP. (L) iBMDMs were treated with LPS and CeeNU prior to ATP stimulation. Cells were then stained with calcein AM (green; viability dye) and PI (red; death marker) to distinguish live and dead populations, respectively. Merged images include fluorescent signals overlaid on the corresponding bright‐field image. Scale bars, 50 µm. (M) PI‐positive and calcein AM‐positive cells were counted in four random fields per sample. Lytic cell death was defined as the ratio of PI‐positive cells to the total cell count (sum of PI‐positive and calcein AM‐positive cells), expressed as a percentage. ^*^ *p* < 0.05, ^***^ *p* < 0.001. Data are mean ± SEM.

Correspondingly, to further investigate the inhibitory efficacy of CeeNU against the NLRP3 inflammasome, we assessed its cytotoxic profile in mouse bone marrow‐derived macrophages (BMDMs). Cell viability assays showed that CeeNU had no obvious cytotoxic effect on BMDMs at concentrations less than 150 µM (Figure 3A). LPS‐primed BMDMs were pretreated with CeeNU followed by nigericin stimulation. CeeNU demonstrated dose‐responsive inhibition of nigericin‐induced IL‐1β secretion, exhibiting an IC_50_ of about 10.199 µM (Figure 3B). Consistent with observations in human macrophages, CeeNU dose‐dependently suppressed ATP‐ or nigericin‐induced LDH release, IL‐1β secretion, caspase‐1 activation, and GSDMD‐NT generation in LPS‐primed BMDMs. However, CeeNU did not significantly affect the expression levels of NLRP3, pro‐IL‐1β, or procaspase‐1 (p45) in whole‐cell lysates (Figure 3C–H). This indicates that CeeNU selectively inhibits NLRP3 inflammasome activation without interfering with its initial priming stage. We also assessed the inhibitory effects of CeeNU on immortalized BMDMs (iBMDMs) treated with ATP (Figure 3I–K), MSU (Figure S2A–C), or nigericin (Figure S2D–F). As expected, CeeNU significantly inhibited the release of IL‐1β and LDH induced by NLRP3 inflammasome activation. Western blot analysis revealed that CeeNU dose‐dependently suppressed the proteolytic processing of IL‐1β (p17), caspase‐1 (p20), and GSDMD‐NT, while exerting no significant effect on the expression levels of procaspase‐1, pro‐IL‐1β, or NLRP3 in iBMDMs. Calcein AM/PI staining showed that CeeNU significantly and dose‐dependently decreased the extent of lytic cell death induced by ATP or nigericin (Figure 3L,M and S2G,H).

Given CeeNU's ability to cross the blood‐brain barrier, we wanted to explore whether it can alleviate inflammation in microglia, the innate immune cells present in the CNS that are related to several neuroinflammatory diseases. Our findings demonstrated that CeeNU suppressed ATP‐induced LDH release, IL‐1β secretion, caspase‐1 activation, and GSDMD‐NT proteolysis in LPS‐primed primary microglia. (Figure S2I–K). Overall, these results demonstrated that CeeNU, which has broad‐spectrum activity, effectively inhibited NLRP3 inflammasome‐induced pyroptosis.

### 2.4 CeeNU Functions as a Selective NLRP3 Inflammasome Inhibitor

**FIGURE 4:**
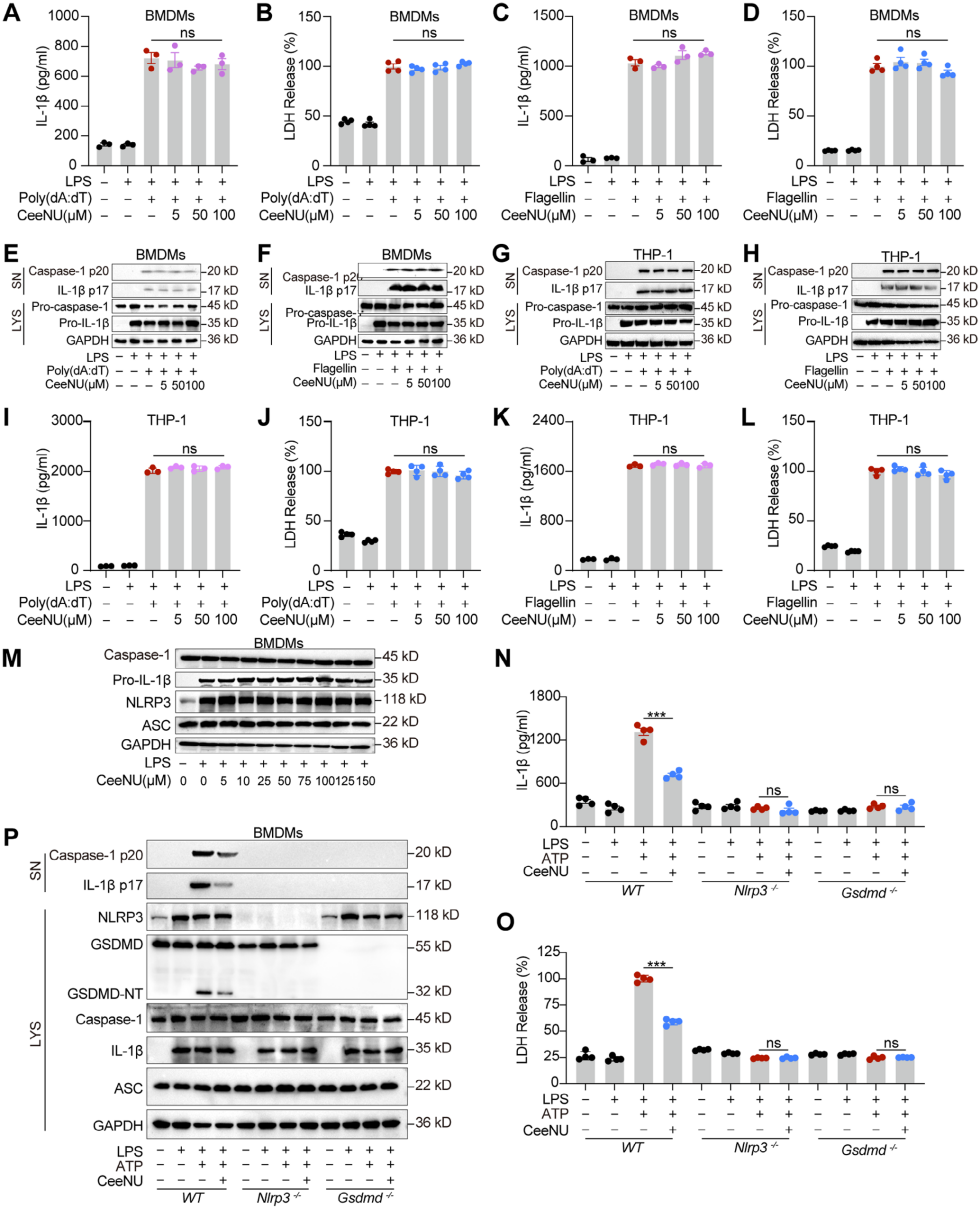
CeeNU is a specific inhibitor of the NLRP3 inflammasome. (A and C) Production of cleaved‐IL‐1β from LPS‐primed BMDMs treated with CeeNU before transfected with poly(dA:dT) (A), flagellin (C). (B and D) LDH release from LPS‐primed BMDMs treated with various doses of CeeNU before transfected with poly(dA:dT) (B), flagellin (D). (E–H) Western blotting analysis of cleaved IL‐1β p17 and caspase‐1 p20 levels in cell supernatant (SN) and pro‐IL‐1β, procaspase‐1, NLRP3, and GAPDH in cell lysates of BMDMs (E and F) and THP‐1 (G and H), LPS‐primed BMDMs and THP‐1 cells treated with CeeNU for 1 h before transfected with poly(dA:dT) for 16 h (E and G), flagellin for 16 h (F and H). (I and K) Production of cleaved‐IL‐1β from LPS‐primed THP‐1 cells treated with CeeNU before transfected with poly(dA:dT) (I), flagellin (K). (J and L) LDH release from LPS‐primed THP‐1 cells treated with various doses of CeeNU before transfected with poly(dA:dT) (J), flagellin (L). (M) BMDMs were treated with or without LPS, and plus different concentrations of CeeNU for 4 h, cell lysates were collected for Western blotting. (N) Western blotting analysis of p20 in SNs, pro‐casp1, and NLRP3 in input from LPS‐primed wild type, *nlrp3* knockout, and *gsdmd* knockout BMDMs treated with 50 µM CeeNU, and then stimulated with ATP for 30 min. (O) ELISA of IL‐1β in SNs from LPS‐primed wild type, *nlrp3* knockout, and *gsdmd* knockout BMDMs treated with 50 µM CeeNU, and then stimulated with ATP for 30 min. (P) LDH release from SN of LPS‐primed wild type, *nlrp3* knockout, and *gsdmd* knockout BMDMs treated with 50 µM CeeNU, and then stimulated with ATP for 30 min. ^***^ *p* < 0.001. Data are mean ± SEM.

Beyond the NLRP3 inflammasome, the absent in melanoma 2 (AIM2) and NLR family CARD domain‐containing 4 (NLRC4) inflammasomes are also capable of activating caspase‐1 and promoting subsequent IL‐1β maturation in response to infectious or damage‐associated signals [35, 36]. Poly(dA:dT) and flagellin are molecules that can activate the immune system to produce an inflammatory response. ELISA and Western blotting analysis showed that CeeNU treatment failed to suppress the AIM2‐ or NLRC4‐mediated cleavage and release of IL‐1β and the activation of caspase‐1 and LDH release in primary BMDMs and THP‐1 cells (Figure 4A–L). Together with the aforementioned results, these results collectively indicated that CeeNU specifically inhibited NLRP3 inflammasome activation, without affecting the AIM2 or NLRC4 inflammasomes.

To determine the mechanism by which CeeNU suppresses NLRP3 inflammasome activation, we examined the pathways upstream of the NLRP3 inflammasome. BMDMs were incubated with CeeNU prior to a 4‐h LPS stimulation. The core components of the NLRP3 inflammasome complex were detected by Western blotting. As shown in Figure 4M, no significant changes in the activation of LPS‐induced upregulated expression of NLRP3 and pro‐IL‐1β were observed, and CeeNU did not alter the expression levels of ASC or caspase‐1 in whole‐cell lysates. These findings suggest that CeeNU does not influence the priming phase of NLRP3 inflammasome activation. Potassium efflux and mitochondrial ROS generation are recognized as a pivotal upstream event that triggers NLRP3 inflammasome activation. We found that CeeNU did not significantly alter nigericin‐triggered ROS production (Figure S3A–D) or potassium efflux (Figure S3E). These results demonstrated that CeeNU had no effect on upstream signaling events. Moreover, CeeNU did not impact the production of tumor necrosis factor (TNF)‐α (Figure S3F).

To further validate the specificity of CeeNU as an NLRP3 inflammasome inhibitor, primary wild‐type (WT), *Gsdmd‐*KO, and *Nlrp3*‐KO mouse BMDMs were stimulated with LPS+ATP to initiate NLRP3 inflammasome activation in the presence or absence of CeeNU. Our data showed that treatment with CeeNU suppressed the release of IL‐1β (Figure 4N) or LDH (Figure 4O) in WT mouse BMDMs but not in *Nlrp3^−/−^* or *Gsdmd^−/−^* mouse BMDMs, suggesting that the inhibitory effects of CeeNU depend on its ability to suppress the NLRP3 inflammasome and GSDMD. This phenomenon was further validated by Western blotting (Figure 4P), which suggested that CeeNU inhibited IL‐1β release and pyroptosis (GSDMD‐NT cleavage) in an NLRP3‐dependent manner.

Previous studies have shown that CeeNU therapy increases tumor DNA damage [37]. To exclude the influence of the antitumor effect of CeeNU on cell apoptosis, we assessed the effect of CeeNU on DNA damage in macrophages. As shown in Figure S3G,H, there was no significant change in the level of phosphorylated histone H2AX (γH2AX), a marker of DNA damage, during LPS/ATP‐induced pyroptosis in macrophages.

### 2.5 CeeNU Specifically Inhibits NLRP3 Inflammasome Assembly

**FIGURE 5:**
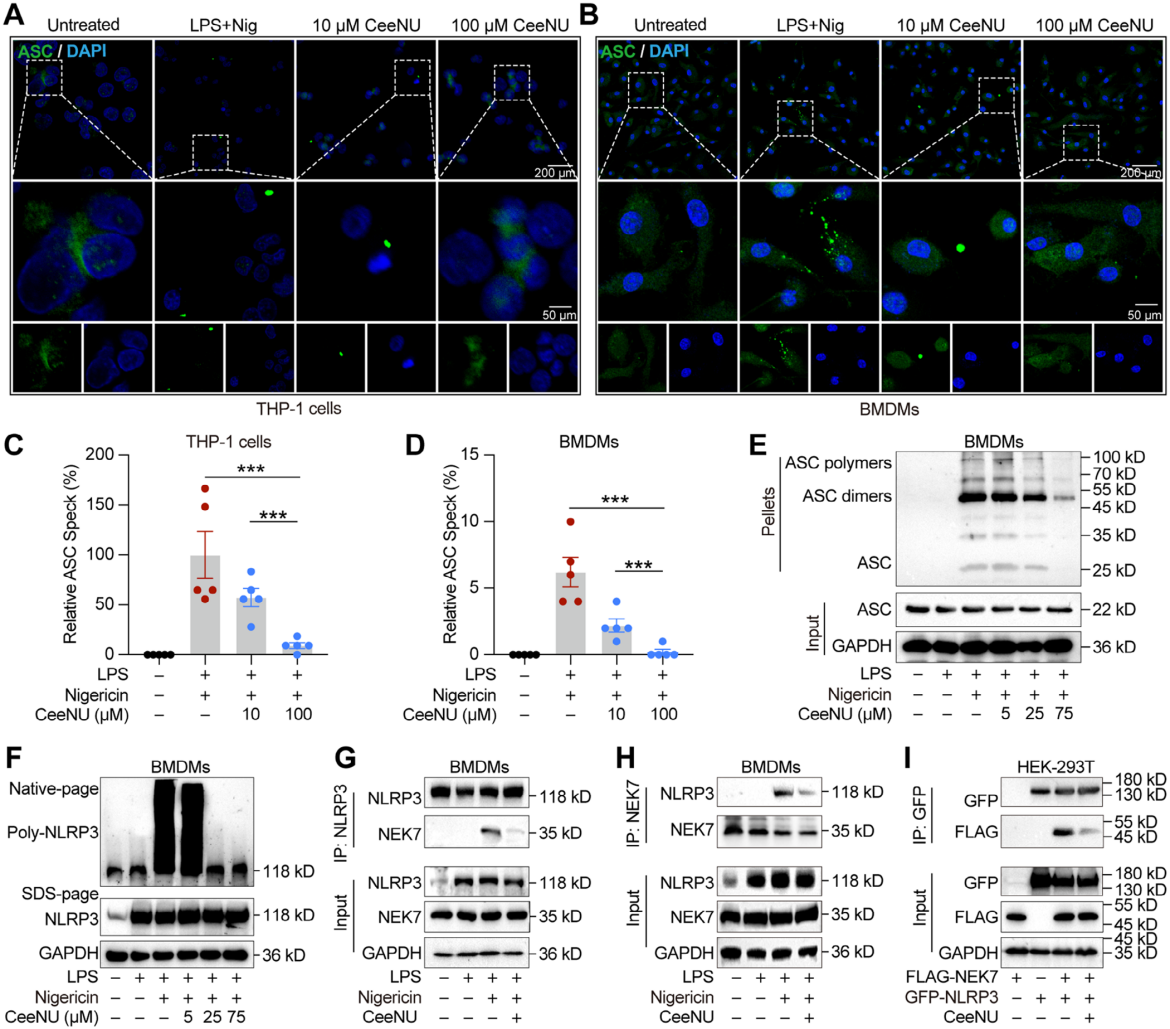
CeeNU specifically inhibits NLRP3 inflammasome assembly. (A–D) The treated THP‐1 (A and C) or BMDMs (B and D) cells were incubated with ASC primary antibody overnight, then re‐stained with secondary antibody for 1.5 h, and then stained with DAPI for 10 min before immunofluorescence images were taken by Olympus confocal microscope. Green represents ASC, DAPI (blue) shows nuclei. Five fields were randomly selected for each group of samples. The circular strong fluorescent spot indicated in the figure by the arrow was ASC speck. After that, the average fluorescence intensity was analyzed by ImageJ software, and the data were processed by GraphPad. (E) Western blotting analysis of ASC oligomerization level after cross‐linking with DSS in BMDMs. (F) Western blotting analysis of NLRP3 self‐oligomerization level of BMDMs pretreated with different doses of CeeNU after LPS stimulation for 3 h, then plus with nigericin. (G and H) BMDMs were primed with LPS, treated with 50 µM CeeNU, and then stimulated with 5 mM nigericin for 1 h. The interaction between endogenous NEK7 and NLRP3 was assessed by co‐immunoprecipitation (co‐IP) using an anti‐NEK7 (or anti‐NLRP3) antibody, followed by immunoblotting with antibodies against the reciprocal protein. (I) HEK293T cells were co‐transfected with plasmids expressing GFP‐NLRP3 and FLAG‐NEK7. Following transfection, cells were treated with 50 µM CeeNU or vehicle control as indicated. Cell lysates were subjected to IP using an anti‐GFP or FLAG antibody, followed by immunoblotting with antibodies against FLAG and GFP. ^**^ *p* < 0.01. Data are mean ± SEM.

We postulated that CeeNU disrupts the assembly of the NLRP3 inflammasome. First, we evaluated the assembly of ASC specks and oligomerization of ASC, which is a critical step for the activation of downstream signaling cascades, such as procaspase‐1 autocatalytic cleavage. Immunofluorescence assays showed that CeeNU significantly attenuated the number of ASC specks in LPS‐primed human THP‐1 cells and murine BMDMs (Figure 5A–D). Next, CeeNU dose‐dependently suppressed nigericin‐induced formation of ASC monomers, dimers, tetramers, and higher‐order oligomeric complexes in LPS‐primed BMDMs (Figure 5E). This effect correlated with its inhibition of IL‐1β secretion, caspase‐1 activation, and GSDMD‐NT generation. In addition, the effect of CeeNU on endogenous NLRP3 self‐oligomerization was analyzed by native PAGE. As shown in Figure 5F, CeeNU treatment significantly inhibited nigericin‐induced endogenous NLRP3 oligomerization. We hypothesized that CeeNU could potentially inhibit the protein–protein interaction between NLRP3 and NEK7, the NEK7–NLRP3 interaction playing a critical role in NLRP3 oligomerization and inflammasome complex formation. Endogenous IP revealed that CeeNU treatment significantly inhibited the NLRP3–NEK7 interaction (Figure 5G,H). We also tested the effect of CeeNU on the interaction between exogenous NLRP3 and NEK7 in HEK293T cells transfected with FLAG‐NEK7 and GFP‐NLRP3 plasmids. As expected, CeeNU markedly disrupted the protein–protein interaction between NLRP3 and NEK7 (Figure 5I). Collectively, these findings indicate that CeeNU attenuates inflammasome activation through suppression of NLRP3 inflammasome assembly.

### 2.6 CeeNU Directly Interacts With the NACHT Domain of NLRP3 at R335

**FIGURE 6:**
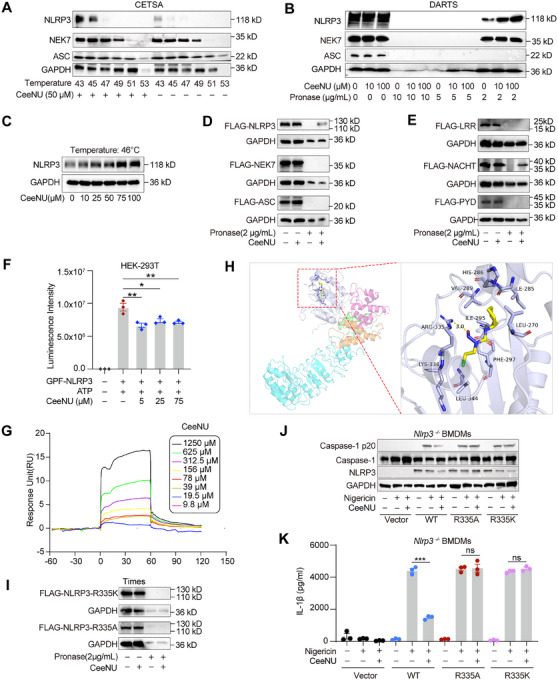
CeeNU directly binds to NLRP3 at Arg335. (A) LPS‐primed BMDMs were treated with 50 µM CeeNU or vehicle control. Cell lysates were then heated at the indicated temperatures, and the thermal stability of NLRP3, NEK7, and ASC was assessed by immunoblotting. (B) DARTS and Western blotting analysis of NLRP3, NEK7, and ASC stability LPS‐primed BMDMs treated with 50 µM CeeNU at different concentrations of pronase (0, 2, 5, and 10 µg/mL). (C) Following treatment of LPS‐primed BMDM lysates with increasing concentrations of CeeNU and heating at 46°C, the thermal stability of NLRP3 was analyzed via immunoblotting. (D) HEK293T cells were transfected with plasmids encoding FLAG‐NLRP3, FLAG‐NEK7, or FLAG‐ASC. Cell lysates were incubated with 50 µM CeeNU or vehicle control, followed by limited proteolysis with pronase (2 µg/mL). The stability of the target proteins was subsequently analyzed by immunoblotting using an anti‐FLAG antibody. (E) HEK293T cells were transfected with constructs encoding the FLAG‐tagged LRR, NACHT, and PYD domains of NLRP3. Cell lysates were incubated with 50 µM CeeNU or vehicle control, followed by limited proteolysis with pronase (2 µg/mL). The protease resistance of each domain was analyzed by anti‐FLAG immunoblotting. (F) After overnight transfection with GFP‐NLRP3, HEK293T cells were treated with increasing doses of CeeNU. The ATPase activity was then quantified. (G) The direct interaction kinetics between CeeNU and immobilized recombinant human NLRP3 protein were measured using single‐cycle kinetics on an SPR biosensor. (H) Molecular docking of CeeNU with NLRP3 by AutoDock 4.2.6. (I) DARTS and immunoblot analysis of R335A or R335K mutant NLRP3 in HEK293T cells transfected with FLAG‐NLRP3‐R335A, and FLAG‐NLRP3‐R335K, and then treated with 50 µM CeeNU and pronase (2 µg/mL). (J) Western blotting analysis of p20 in SNs, pro‐casp1, and NLRP3 in input from NLRP3 knockout BMDMs recombined with human WT or R335A or R335K mutant NLRP3, then stimulated with LPS‐nigericin. (K) ELISA of IL‐1β in SNs from NLRP3 knockout BMDMs recombined with human WT or R335A or R335K mutant NLRP3. ^*^ *p* < 0.05, ^**^ *p* < 0.01, ^***^ *p* < 0.001. Data are mean ± SEM.

Based on the above results, we speculated that CeeNU may directly bind to protein components of the NLRP3 inflammasome. To test this hypothesis, we employed drug affinity responsive target stability (DARTS) combined with mass spectrometry to identify the protein targets that directly interact with CeeNU [25]. Interestingly, mass spectrometry revealed that NLRP3 was a CeeNU‐interacting protein (Figure S4, Table S2). To further validate the binding of CeeNU to NLRP3, a cellular thermal shift assay (CETSA) [38] and a DARTS assay, which are well‐established techniques for determining drug‐target interactions, were used. CETSA showed that preincubation of BMDMs with CeeNU (50 µM) increased the thermostability of the NLRP3 protein but not that of ASC or NEK7 and retarded the heat‐mediated degradation of NLRP3 (Figure 6A). In addition, the DARTS assay showed that the preincubation of BMDMs with CeeNU in cell lysates effectively suppressed the pronase‐induced proteolysis of the NLRP3 protein (Figure 6B). There is no proteolysis for GAPDH and NEK7 upon pronase treatment at 2 µg/mL, which may be due to the differences in protein structure. Furthermore, the heat‐mediated degradation of NLRP3 was inhibited with increasing CeeNU at the half‐maximal inhibitory temperature of 46°C (Figure 6C). DARTS and CETSA assays suggested that the anti‐inflammasome activity of CeeNU was mediated by direct and selective targeting NLRP3. Subsequently, a DARTS assay was conducted in HEK293T cells transiently transfected with plasmids encoding NLRP3, NEK7, and ASC. (Figure 6D). We found that NLRP3, but not ASC or NEK7, bound to CeeNU. To determine the domain involved in the interaction between CeeNU and NLRP3, we transfected plasmids containing three domains of NLRP3—LRR, NACHT, and PYD—in HEK293T cells for the DARTS assay. Our findings revealed that CeeNU specifically interacts with the NACHT domain, but not with the LRR or PYD domains (Figure 6E). The NACHT domain is essential for NLRP3 oligomerization, primarily mediated by its ATPase function; therefore, we assessed the potential effect of CeeNU on the ATPase activity of NLRP3. As shown in Figure 6F, CeeNU repressed the ATPase activity of NLRP3, suggesting that CeeNU suppresses NLRP3 activation by targeting its ATPase activity. To confirm direct binding and to quantify its affinity, we performed surface plasmon resonance (SPR) assays between CeeNU and recombinant human NLRP3 protein (containing the NACHT domain). As shown in Figure 6G, CeeNU directly bound to the recombinant hNLRP3 protein in a dose‐dependent manner, with a KD value of 19.26 µmol/L. In addition, molecular docking model analysis was utilized to determine the specific site at which CeeNU binds to NLRP3. The results showed that the binding energy between CeeNU and human NLRP3 was −6.1 kcal/mol, indicating significant affinity. CeeNU engages the NACHT domain of NLRP3 by forming hydrogen bonds with Arg335. In addition, it engages in hydrophobic interactions with Lys336, Leu344, Phe297, Leu270, Ile285, His286, Val289, and Ile295 (Figure 6H). To determine the binding between CeeNU and Arg335 in NLRP3, we constructed an NLRP3 plasmid with a mutation of amino acid 335. The DARTS results showed that replacing the R335 residue with lysine or alanine abolished the stability of CeeNU for NLRP3(Figure 6I). Furthermore, CeeNU suppressed NLRP3 inflammasome activation in NLRP3‐deficient BMDMs reconstituted with WT human NLRP3, whereas no inhibitory effect was observed in cells expressing R335A or R335K NLRP3 mutants (Figure 6J,K). Taken together, these findings demonstrate that CeeNU directly binds to the NACHT domain of NLRP3 to attenuate inflammasome activation, with Arg335 identified as an essential residue for its inhibitory function.

### 2.7 CeeNU Significantly Alleviates MSU‐Induced Peritonitis and Gouty Arthritis

**FIGURE 7:**
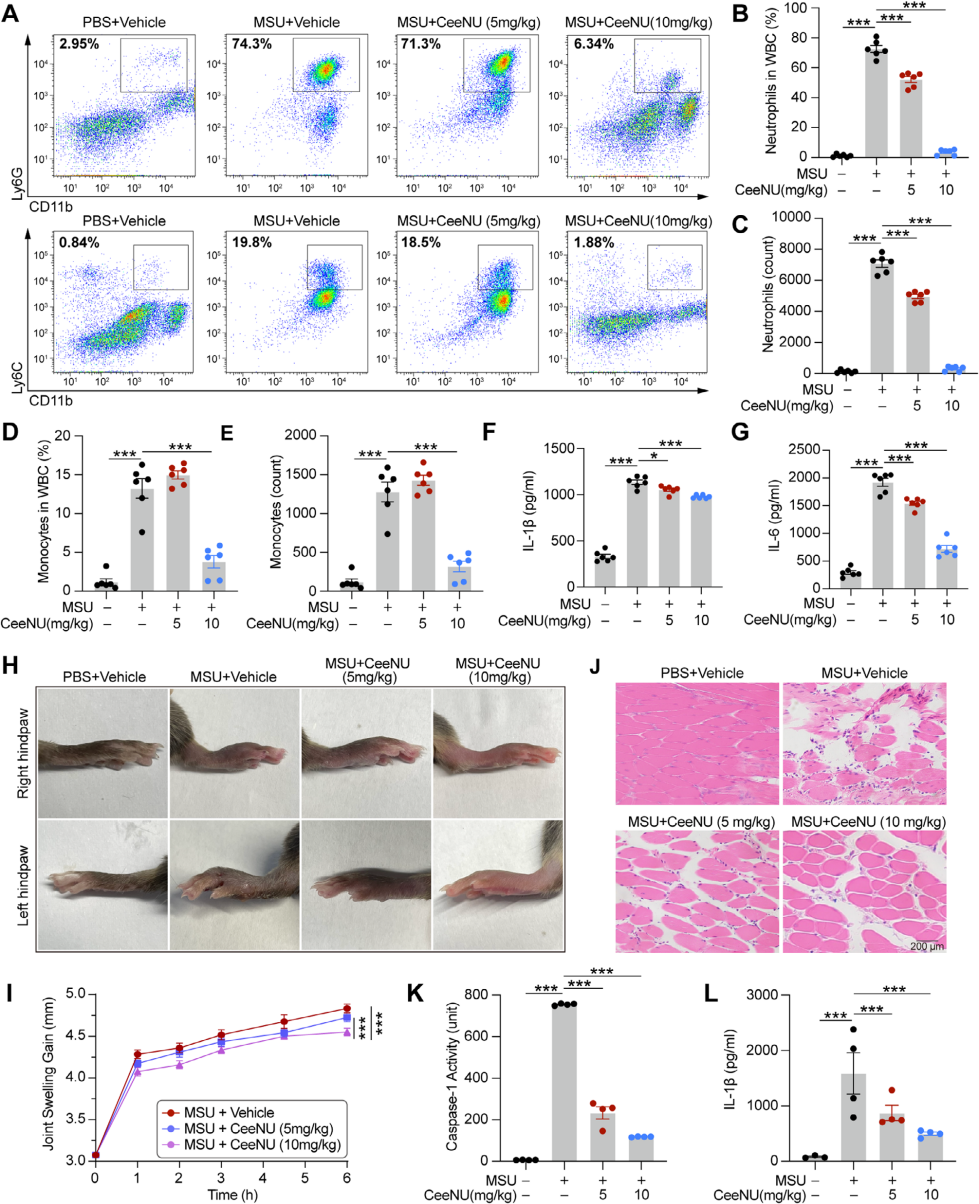
CeeNU significantly alleviates MSU‐induced peritonitis and gouty arthritis. Eight‐week‐old male C57BL/6 mice were intraperitoneally injected with 100 µL vehicle control or 100 µL CeeNU before intraperitoneal injection with 100 µL PBS or 1 mg MSU (dissolved in 100 µL PBS). (A) Flow cytometry analysis of neutrophils (CD11b^+^ and Ly6G^+^) and monocytes (CD11b^+^ and Ly6C^+^) in peritoneal lavage fluid. Statistical analysis of flow cytometry results of neutrophil percentage in white blood cells (B), neutrophil number (C), monocyte percentage in white blood cells (D), and monocyte number (E). (F) ELISA of IL‐1β levels in the supernatants of peritoneal lavage fluid. (G) ELISA of IL‐6 levels in the supernatants of peritoneal lavage fluid. (H) The photograph of mice joint swelling level. (I) Mice joint thickness after injection of MSU (0.8 mg in 20 µL PBS). (J) H&E staining of the joint tissue. (K) Caspase‐1 activity in the foot joints of mice. (L) IL‐1β levels in the foot joints of mice. ^*^ *p* < 0.05, ^***^ *p* < 0.001. Data are mean ± SEM.

Subsequently, we investigated the effect of CeeNU on NLRP3 inflammasome activity in a murine model of MSU‐induced peritonitis, a pathology previously demonstrated to be critically dependent on NLRP3. To evaluate the efficacy of CeeNU, mice were administered either a vehicle control or CeeNU prior to intraperitoneal injection of either PBS or MSU. Flow cytometric analysis of peritoneal lavage fluid indicated that both the proportion and absolute number of neutrophils (CD11b^+^ and Ly6G^+^) and monocytes (CD11b^+^ and Ly6C^+^) were markedly reduced in CeeNU‐treated mice compared to controls (Figure 7A–E). In addition, IL1β and IL6 levels were assessed in supernatants of peritoneal lavage fluid, and the results demonstrated a significant decrease in the levels of these cytokines in CeeNU‐treated mice compared to those in control mice (Figure 7F,G). These findings underscore CeeNU as a potential therapeutic agent for the treatment of MSU‐induced peritonitis.

Inflammatory models linked to the activation of the NLRP3 inflammasome include not only peritonitis induced by MSU but also gouty arthritis provoked by MSU [39, 40]. Gouty arthritis, a condition triggered by MSU crystal deposition in joints and periarticular tissues, is pathogenically linked to dysregulated NLRP3 inflammasome activation. To ascertain the efficacy of CeeNU in this regard, mice were administered MSU in their footpads to induce acute gouty arthritis, followed by treatment with either CeeNU or a vehicle control. The thickness of the joints of the mice was measured after MSU injection, and the data revealed that CeeNU treatment significantly reduced the joint thickness compared to that of the vehicle control. Photographic evidence of the paws of the mice confirmed that CeeNU treatment alleviated joint swelling to a greater extent than the vehicle control (Figure 7H,I). To further investigate the effects of CeeNU on acute gouty arthritis, we performed histological staining of the joint tissue. The staining results revealed that CeeNU effectively mitigated MSU‐induced tissue damage, as evidenced by the preservation of the normal architecture of the joint tissue (Figure 7J). Furthermore, CeeNU treatment markedly inhibited the activity of caspase‐1 in mouse foot joints, indicating that CeeNU may induce an anti‐inflammatory response in these joints (Figure 7K). This suggestion is further bolstered by the significant reduction in IL‐1β levels observed in the paw joints of mice treated with CeeNU. IL‐1β, a pro‐inflammatory cytokine, plays a pathogenic role in gouty arthritis by inducing the expression of additional inflammatory mediators. The decrease of IL‐1β levels observed with CeeNU treatment implied an anti‐inflammatory effect, which is likely responsible for the alleviation of symptoms associated with gouty arthritis (Figure 7L). These findings unequivocally demonstrate the effectiveness of CeeNU in alleviating acute gouty arthritis in mice. Overall, the data presented in this study suggest that CeeNU holds promise as a therapeutic agent for managing these conditions in humans.

### 2.8 CeeNU Effectively Prevents MOG‐Induced EAE

**FIGURE 8:**
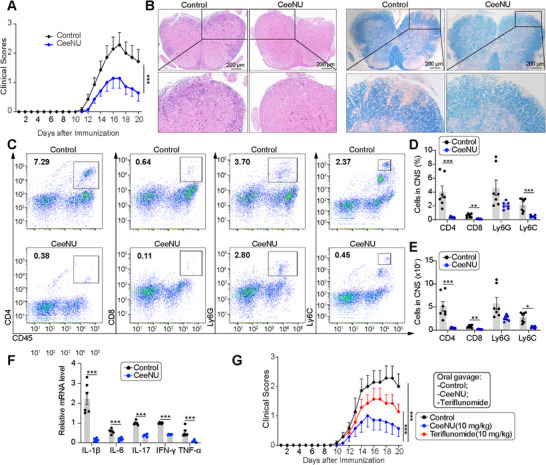
CeeNU effectively prevents MOG‐induced EAE. (A) Clinical scores of EAE mice treated with CeeNU or control. WT mice were immunized with MOG_35–55_ antigen and treated with CeeNU (*n* = 7) or PBS (*n* = 7) daily from Day 0 by intraperitoneal injection. (B) Spinal cord sections from mice at the peak of disease were stained with H&E (to visualize inflammatory foci) and LFB (to stain myelin). (C) Flow cytometry analysis of neutrophils (CD45^+^ and Ly6G^+^), monocytes (CD45^+^ and Ly6C^+^), CD4+ and CD8+ cells in EAE mice brain at Day 20. (D) Percentage and (E) number of different cells from flow cytometry data. (F) Total RNA was extracted from the spinal cord tissues of mice. The relative mRNA expression levels of IL‐1β, IL‐6, IL‐17A, TNF‐α, and IFN‐γ were determined by quantitative real‐time PCR (qRT‐PCR) and normalized to the GAPDH levels. (G) Comparison of clinical scores of teriflunomide and CeeNU in EAE model mice. ^*^ *p* < 0.05, ^**^ *p* < 0.01, ^***^ *p* < 0.001. Data are mean ± SEM. EAE clinical score by two‐way ANOVA.

The EAE mouse model faithfully recapitulates the clinical manifestations of MS, a human disease characterized by T cell‐mediated inflammation and demyelination of the CNS due to self‐antigens [41]. The critical involvement of the NLRP3 inflammasome in the EAE model has been well established [42]. Thus, we investigated the potential therapeutic effects of CeeNU on EAE. Our findings demonstrated that EAE mice treated with daily CeeNU (at a dosage of 10 mg/kg) exhibited lower EAE clinical scores compared to EAE mice treated with vehicle (Figure 8A). As assessed by hematoxylin and eosin (H&E) and luxol fast blue (LFB) staining, CeeNU treatment led to a conspicuous decrease in immune cell infiltration and demyelination within the spinal cords of EAE mice (Figure 8B). Furthermore, flow cytometry analysis of mouse brain tissue revealed a significant decrease in the number and proportion of leukocytes, monocytes, CD4+ cells, and CD8+ cells in mice treated with CeeNU (Figure 8C–E). Moreover, CeeNU administration led to a marked reduction in the mRNA levels of pro‐inflammatory cytokines (IL1β, IL6, IL17A, TNF‐α, and IFN‐γ) within the spinal cord (Figure 8F). To further clarify the clinical application value of CeeNU, we administered CeeNU via oral gavage to EAE mice and compared the effects of CeeNU with teriflunomide, a first‐line clinical drug for MS. EAE clinical scores showed that CeeNU had a significantly better therapeutic effect on EAE than teriflunomide (Figure 8G). These results suggest that CeeNU decreases the severity of EAE and is a promising therapeutic candidate for the treatment of MS.

## 3 Discussion

NLRP3‐mediated pyroptosis and inflammatory cytokine release play critical roles in the exacerbation of a series of autoimmune or inflammatory diseases, including sepsis, MS, gouty arthritis, etc. [43, 44, 45], suggesting that NLRP3 is an extremely attractive checkpoint for drug development. Although NLRP3 has been recognized as central to these diseases, almost no related drugs have been clinically translated due to their toxicity and efficacy, suggesting that the selection of clinically applicable drugs is urgently needed. ATP‐based cell viability assays are the most sensitive analyses under acute cytotoxic effects and have been widely used for detecting pyroptosis [34, 46]. Herein, by evaluating ATP‐based cell viability, we developed a high‐throughput experimental screen for cell pyroptosis inhibitors and successfully identified CeeNU as a highly selective NLRP3 inhibitor from a library of 2747 FDA‐approved clinical drugs.

The release of inflammatory cytokines mediated by the NLRP3 inflammasome exerts a dual role in the pathogenesis of human diseases. Although inflammatory cytokines contribute detrimentally to the pathogenesis of inflammatory and metabolic disorders, they play beneficial roles in some cancers, such as glioblastoma [13, 47, 48]. CeeNU is well known as a well‐tolerated FDA‐approved drug for the treatment of melanoma, glioblastoma, and leukemia [29, 31, 49, 50]. Moreover, many recent studies have shown that the combination of CeeNU and other drugs significantly increases median survival and has a high number of curative effects [51]. The inhibition of NLRP3 inflammasome activation by CeeNU may be the mechanism by which CeeNU exerts its antitumor effects. The specific details need to be further studied.

In recent years, a number of antineoplastic agents have also been characterized as NLRP3 inflammasome inhibitors. Beyond its established role as a potent multikinase inhibitor against NTRK, ROS1, and ALK, entrectinib (ENB) has now been identified as an NLRP3 inflammasome inhibitor. It functions by directly binding to the arginine 121 (R121) residue of NEK7, which blocks the interaction between NEK7 and NLRP3 [52]. Another anticancer drug, ergolide, has been shown to have anticancer effects on several human tumor cell lines. Recent research has identified ergolide as an inhibitor of the NLRP3 inflammasome that operates via covalent modification of NLRP3, effectively blocking pyroptosis [53]. Similarly, the antitumor STAT3/JAK inhibitor brevilin A inhibits NLRP3 inflammasome activation in vivo and in vitro by influencing the upstream pathway of NLRP3‐induced ASC oligomerization [54]. In this study, we found that the anticancer drug CeeNU profoundly repressed NLRP3 inflammasome activation and IL‐1β release in both mouse and human macrophages. CeeNU inhibited various inflammatory ligands that triggered NLRP3 activation, suggesting its potential as a robust therapeutic agent for a spectrum of autoimmune and inflammatory disorders. Given that inflammasome activation results in GSDMD cleavage and cell membrane rupture, it is important to determine whether the reduced IL‐1β release and pyroptosis are due to the inhibition of NLRP3 or suppression of its upstream molecules. Our results demonstrated that AIM2 and NLRC4 inflammasome activation remains unchanged by CeeNU treatment, thus establishing its specific inhibitory activity against the NLRP3 inflammasome. In addition, CeeNU did not influence upstream signaling events preceding NLRP3 activation, including mitochondrial ROS generation and potassium efflux. Moreover, our results indicated that CeeNU does not alter LPS‐induced priming of the NLRP3 inflammasome, gene expression, or synthesis of the IL‐1β precursor. However, CeeNU specifically blocked NLRP3 inflammasome complex assembly and repressed the cleavage of IL‐1β and GSDMD in macrophages and microglia.

One of the critical questions is whether CeeNU interacts with NLRP3 or also interacts with ASC or NEK7, which are related to the assembly of the NLRP3 inflammasome complex. Most inhibitors, such as tranilast [55], MCC950 [42], mefloquine [56], and costunolide [39], suppress NLRP3 inflammasome activation through direct binding to NLRP3. In addition to targeting NLRP3 itself, some inhibitors also act by binding to other components of the inflammasome, such as ASC and NEK7. Recent research has demonstrated that spirodalesol analog 8A exerts its inhibitory effect on the NLRP3 inflammasome via direct interaction with the adaptor protein ASC [57]. ENB inhibits the NLRP3 inflammasome by directly binding to R121 of NEK7 [52]. Licochalcone B acts by directly binding to NEK7, which selectively inhibits the NLRP3 inflammasome through disruption of the NLRP3–NEK7 interaction and subsequent blockade of inflammasome activation [58]. Thiolutin inhibits BRCC3‐containing isopeptidase complex‐mediated NLRP3 deubiquitination and activation. 1,2,4‐Trimethoxybenzene selectively inhibits NLRP3 inflammasome activation, attenuates EAE, and inhibits the oligomerization of ASC and the interaction between NLRP3 and ASC, thus blocking NLRP3 inflammasome assembly [9]. By suppressing NLRP3 inflammasome activation, itaconate and its derivative 4‐OI (4‐octyl itaconate) specifically inhibit the interaction between NLRP3 and NEK7 via “dicarboxypropylated” C548 on NLRP3 [59]. OLT1177 directly targets NLRP3, which not only disrupts its interaction with ASC and suppresses subsequent oligomerization but also attenuates the ATPase activity of recombinant NLRP3 in vitro. Mechanistically, OLT1177 had no significant effect on potassium efflux, gene expression, or IL‐1β synthesis [60]. In this study, CETSA and DARTS assays provided evidence that the anti‐inflammasome activity of CeeNU is mediated through the direct targeting of NLRP3. Given the numerous potential targets identified via mass spectrometry (Table S2), PARP1 is also known to regulate NLRP3 [61]. Whether CeeNU regulates NLRP3 through binding with PARP1 requires further in‐depth investigation.

To elucidate the mechanism underlying the interaction between NLRP3 and CeeNU, based on the published crystal structure, we generated an interaction model between NLRP3 and CeeNU and found that CeeNU bound to the NACHT domain of NLRP3, forming hydrogen bonds with Arg335. In general, hydrogen bonding exhibits a higher interaction strength compared to hydrophobic interactions. Therefore, we selected Arg335 for an in‐depth investigation to elucidate the underlying molecular mechanisms. Homologous alignment revealed that the NLRP3 Arg335 site is conserved in both humans and mice (Figure S5). Some key amino acid sites of NLRP3 have become targets for drug development. Costunolide targeting of cysteine 598 in NLRP3 alters the ATPase activity and assembly of the NLRP3 inflammasome [39]. Britannin interacts directly with the NLRP3 NACHT domain at residues Arg335 and Gly271, disrupting the assembly process by specifically inhibiting the NLRP3–NEK7 interaction [62]. Oridonin covalently modifies cysteine 279 (Cys279) within the NLRP3 NACHT domain, thereby preventing NLRP3–NEK7 interaction and subsequent inflammasome assembly and activation [19]. By generating a point mutation in the recombinant NLRP3 protein, we successfully identified that CeeNU interacted with NLRP3 at Arg335. Arg335 was also predicted to be the specific binding site that mediates the inhibitory effects of britannin [62], clioquinol [63], and alantolactone [64]. Replacing the R335 residue with lysine or alanine substantially abolished the inhibitory effect of CeeNU, indicating that this amino acid residue is critical for CeeNU inhibition of NLRP3.

In addition, our results showed that CeeNU has significant therapeutic efficacy in several experimental mouse models, such as EAE, MSU‐induced gouty arthritis, and peritonitis. CeeNU markedly impeded EAE and displayed better therapeutic effects than did teriflunomide [65], a first‐line clinical drug for MS. CeeNU administration attenuated the progression and pathological features of MSU‐induced gouty arthritis and peritonitis. To evaluate the therapeutic efficacy of CeeNU under physiological conditions, its impact was further investigated in a sepsis model. As shown in Figure S6A, it was observed that intraperitoneal administration of CeeNU significantly enhanced the survival rate in a murine model of LPS‐induced sepsis. Consistently, H&E staining also demonstrated that CeeNU significantly alleviated the infiltration of immune cells and lung injury (Figure S6B). In addition, the administration of CeeNU resulted in a marked decrease in serum levels of IL‐1β, IL‐6, and TNF‐α, indicating a decisive anti‐inflammatory effect against endotoxin shock (Figure S6C–E). Overall, CeeNU represents a highly specific and potent NLRP3 inhibitor, demonstrating considerable therapeutic potential for a range of autoimmune and inflammatory disorders.

As an alkylating chemotherapeutic agent with antitumor properties, CeeNU commonly presents side effects such as bone marrow suppression and hepatorenal toxicity, which limit its clinical application. According to relevant literature reports, the dosage of CCNU (CeeNU) used in mouse models is 20 mg/kg [70]. To avoid the impact of side effects, we selected concentrations below 10 mg/kg for related model studies. In addition, future research could focus on leveraging nanomaterial‐based targeted drug delivery systems and structural optimization strategies to mitigate or inhibit the progression of these adverse effects.

Overall, our findings identified CeeNU as a potential and effective NLRP3 inflammasome inhibitor from a library of 2747 FDA‐approved drugs that strongly inhibit NLRP3‐mediated pyroptosis. By suppressing ASC oligomerization and disrupting the NLRP3–NEK7 interaction, CeeNU inhibits NLRP3 inflammasome assembly. We further established that CeeNU engages the NACHT domain of NLRP3, thereby suppressing NLRP3 inflammasome activation. We identified that Arg335 might be a critical residue for the interaction between human NLRP3 and CeeNU. Importantly, CeeNU confers protection in murine models of NLRP3 inflammasome‐driven pathologies, including MOG‐induced EAE, LPS‐induced septic shock, and MSU‐induced peritonitis and gouty arthritis. Collectively, these data identify CeeNU as a selective NLRP3 inhibitor and a viable therapeutic candidate for NLRP3 inflammasome‐driven pyroptotic disorders.

Notwithstanding the compelling evidence positioning CeeNU as a potent and specific NLRP3 inhibitor with demonstrated therapeutic efficacy across multiple preclinical models, certain limitations merit consideration. Primarily, despite our identification of Arg335 within the NACHT domain as a critical binding residue for CeeNU, the precise structural mechanisms underlying this interaction—including potential conformational rearrangements in NLRP3 or modulation of its ATPase activity—remain incompletely resolved and necessitate further investigation via structural approaches such as X‐ray crystallography or cryo‐electron microscopy. Moreover, whereas CeeNU exhibited consistent efficacy in murine systems, its translational applicability to human subjects with chronic NLRP3‐mediated pathologies requires validation in controlled clinical settings. In addition, this study did not assess the potential effects of sustained CeeNU administration on alternative inflammasome pathways or broader immune homeostasis, thereby underscoring the need for extended mechanistic and toxicological profiling.

## 4 Materials and Methods

### 4.1 Materials

FDA‐approved Drug Library (L1300) and CeeNU (Lomustine) (S1840) were purchased from Selleck Chemicals (Houston, TX, USA). The CellTiter‐Glo Luminescent Cell Viability Assay kit was obtained from Promega (Madison, WI, USA). Antibodies to GSDMD (ab219800, ab209845) were from Abcam (Cambridge, UK). H2A.X (phospho S139) was from Abcam. Anti‐capase‐1 (AG‐20B‐0042) and anti‐NLRP3 (AG‐20B‐0014) antibodies were purchased from Adipogen (San Diego, CA, USA). Anti‐ASC (SC‐22514‐R) antibody was from Santa Cruz Biotechnology (Dallas, Texas, USA). Anti‐IL‐1β (AB‐401‐NA) antibody was obtained from R&D (Minneapolis, Minnesota, USA). Anti‐Flag (F1804‐200UG), LPS (L2630), ATP (A6419), Nigericin (481990), Pronase E (1.07433), and PMA (P1585) were from Sigma. Anti‐GAPDH was from Cell Signaling Technology (5174S) (Danvers, MA, USA). DMEM (31985‐070), RPMI 1640 (11875119), and FBS (10091148) were from Gibco (Carlsbad, CA, USA). Poly(dA:dT) was from CA (6249‐42‐01). LPS‐B5 Ultrapure (tlrl‐pb5lps), Nigericin (tlrl‐nig), and Poly(dA:dT) (tlrl‐patn) were from InvivoGen (San Diego, California, USA). MSU crystals (tlrl‐msu) and TRIzol (15596026) were from Invitrogen (Carlsbad, CA, USA). BCA Protein Assay Kit (23225) was from Thermo (MA, USA). CFA (F5881) and M. tuberculosis H37Ra (231141) were from BD Diagnostics (Franklin Lakes, New Jersey, USA). Anti‐mouse IL‐1β, Human IL‐1β, IL‐6, and TNF‐α ELISA kits (CEK1788, CEK1731, CEK1785, CEK1783) were from Bioworld (USA). Cytotoxicity (LDH) assay kit (C10007), Caspase‐1 activity assay kit (C1102), and Flagellin (P7388) were from Beyotime (Shanghai, China). Anti‐CD4‐APC (317416, 1:400) and anti‐CD45‐PE‐Cyanine7 (25‐0451‐82, 1:400) antibodies were from Biolegend (San Diego, CA, USA). Anti‐CD8‐FITC (11‐0081‐85, 1:400, 1:400), anti‐F4/80‐Brilliant 711 (BM8, 1:200), anti‐CD11b‐Alexa Fluor 488 (53‐0112‐82), anti‐LY6C‐Brilliant 421 (HK1.4), and anti‐LY6G‐PE (12‐5931‐81, 1:500) antibodies were from eBioscience (San Diego, CA, USA). Lipofectamine 3000 (L3000008) was from Invitrogen (California, USA). Pertussis toxin (P7208) was from List Biological Laboratories (Campbell, California, USA). MOG_35–55_ peptide (MEVGWYRSPFSRVVHLYRNGK) was synthesized in Nanjing Peptide Biotech. Plasmids were commercially sourced from Wuhan Miaoling Biotechnology Co. Ltd. (Wuhan, China). The lentiviruses encoding WT NLRP3 and its mutants (R335A, R335K) were custom‐generated by Nanjing Corues Biotechnology Co. Ltd. (Nanjing, China).

### 4.2 Cell Culture

Mouse iBMDM cell line was preserved in our laboratory. Human THP‐1 cell line (THP‐1 ATCC TIB‐202) was from ATCC. iBMDMs and THP‐1 cells were cultured in DMEM‐high glucose and RPMI 1640 medium, respectively. Both media were supplemented with 10% heat‐inactivated fetal bovine serum (FBS; Gibco, 10099141 for THP‐1) and 1% penicillin/streptomycin (P/S). All cultures were grown at 37°C under 5% CO_2_.

### 4.3 Primary Cell Isolation and Culture

#### 4.3.1 Differentiation of Macrophages Derived From Bone Marrow

C57BL/6 mice were euthanized by cervical dislocation and surface‐sterilized with 75% ethanol. Bone marrow cells were isolated from the femora and tibiae and cultured in BM‐Mac medium—composed of 80% DMEM, 10% FBS, 1% P/S, and 20% L929 cell‐conditioned medium containing M‐CSF—at 37°C under 5% CO_2_. After harvesting the 7‐day adherent BMDMs with a cell scraper, the cells were seeded in 6‐well plates at 1.6 × 10^6^ cells per well using 2 mL of complete DMEM medium for subsequent experiments. Following overnight incubation, the cells were utilized for subsequent experiments.

#### 4.3.2 Isolation and Culture of Primary Microglia

Primary microglial cells were isolated from postnatal Day 1 (P1) mice following established protocols. Briefly, neonates were anesthetized on ice and surface‐disinfected with 75% ethanol. Cerebral cortices were aseptically dissected, followed by removal of meninges and superficial vasculature. After 15 min of digestion with 0.125% trypsin at 37°C, the enzymatic activity was quenched with serum‐supplemented medium. The pellet was collected by centrifugation (1000 rpm, 5 min), subsequently resuspended, and transferred to 75 cm^2^ culture flasks coated with poly‐L‐lysine. Following a 14‐day incubation period, microglia were detached via trypsinization, collected, centrifuged, and resuspended in complete medium. Cells were then seeded into pre‐coated 24‐well plates for experimental use.

### 4.4 Inflammasome Activation

THP‐1 cells, iBMDMs, or microglial cells were seeded into 6‐well plates for NLRP3 inflammasome activation assays. After overnight culture, the cells were primed with 100 ng/mL LPS for 3 h (200 ng/mL for iBMDM, THP‐1, or microglia). Cells were treated with graded concentrations of CeeNU for 1 h and subsequently exposed to either ATP (5 mM, 1 h), nigericin (10 µM, 1 h), or MSU (200 µg/mL, 6 h). To stimulate the AIM2 and NLRC4 inflammasomes, 2 µg/mL poly(dA:dT) was transfected into the BMDMs for 16 h, and 1 µg/mL flagellin was transfected into the BMDMs for 16 h.

### 4.5 Measurement of Cell LDH Release

The cell treatments were the same as described above. To assess cell death, the levels of LDH in the cell culture supernatant were measured. LDH measurement was carried out following the instructions of a Cytotoxicity detection kit (LDH) from Roche, and all values were normalized based on LDH release induced by LPS plus ATP (nigericin/MSU) stimulation.

### 4.6 Calcein AM/PI Staining

Mouse primary macrophages were seeded in a 12‐well plate and treated using the same method as the above‐mentioned typical NLRP3 inflammasome activation protocol. Calcein AM was diluted with DMSO to a concentration of 1 µg/µL, and PI was diluted with sterile water to a ratio of 1 mg/mL. Then, 10 µL/15 µL of the diluted calcein AM/PI was added to 5 mL PBS, and the mixture was aliquoted into the prepared 12‐well plate. The plates were maintained at 37°C for 15 min, and randomly five fields were captured using an immunofluorescence electron microscope. The obtained images were processed using ImageJ to obtain the proportion of cell death data.

### 4.7 Scanning Electron Microscope

THP‐1 cells were seeded onto sterile cover glasses within petri dishes and stimulated with LPS/nigericin to induce NLRP3 inflammasome activation in the presence or absence of CeeNU. The culture medium was aspirated, and slides were gently rinsed with PBS before adding electron microscopy fixative to the dish. Following fixation for 2 h at room temperature, samples were transferred to 4°C for storage and transport. After three 15‐min washes in 0.1 M phosphate buffer (PB, pH 7.4), the tissue blocks were subsequently incubated at room temperature for 1–2 h in 1% osmium tetroxide (OsO_4_), which was prepared in the same 0.1 M PB (pH 7.4). After additional washes in 0.1 M PB (pH 7.4) under the same conditions, dehydration was performed through a graded ethanol series (30%, 50%, 70%, 80%, 90%, 95%, and two changes of 100% ethanol, 15 min each), followed by isoamyl acetate for 15 min. Coverslips were critical‐point dried using a Quorum K850 system, mounted on sample holders, and imaged with a field emission scanning electron microscope (SU8100, Hitachi, Japan).

### 4.8 Western Blotting

Western blot analysis was carried out as previously reported [66]. Briefly, tissue or cell samples were lysed in RIPA buffer containing protease and phosphatase inhibitors. Protein concentration was determined using a BCA assay, and equal amounts of protein were separated by SDS‐PAGE and electrophoretically transferred to polyvinylidene fluoride (PVDF) membranes (Amersham Bioscience). After blocking with 5% non‐fat milk in Tris‐buffered saline (TBS) for 2 h, the membranes were incubated with specific primary antibodies overnight at 4°C. After washing, membranes were probed with appropriate secondary antibodies. Chemiluminescent detection was performed using SuperSignal West Pico substrate (Thermo Scientific, 34577), and signal acquisition/analysis was conducted on a Tanon imaging system.

### 4.9 IP Assay

First, after PBS washing, cells were lysed for 1 h at 4°C with IP lysis buffer (150 mM NaCl, 100 mM Tris‐HCl [pH 8.0], 1% Triton X‐100, 2 mM EDTA). Next, the lysates were sonicated and centrifuged (12,000 × g, 30 min, 4°C). Finally, the cleared supernatants were immunoprecipitated by incubation with specific primary antibodies (2 µg) for 8–12 h at 4°C, followed by incubation with either protein A/G agarose (Santa Cruz Biotechnology, sc‐2003) for 6 h or anti‐c‐Myc agarose (Sigma‐Aldrich, A7470) overnight at 4°C.

### 4.10 Cross‐Linking of ASC Oligomers

First, BMDMs were cultured overnight in 6‐well plates at 37°C under 5% CO_2_. After treatment, cells were lysed in ice‐cold PBS containing 0.5% Triton X‐100 and centrifuged at 6000 × g for 15 min at 4°C. Next, the pellets were washed twice with PBS, resuspended in 200 µL PBS, and cross‐linked with 2 mM BS^3^ for 30 min at room temperature. Finally, following another centrifugation step (6000 × g, 15 min, 4°C), the samples were resuspended in 20 µL of 5× SDS‐PAGE loading buffer, boiled for 5 min, and prepared for Western blot analysis.

### 4.11 Assay for NLRP3 Oligomerization

Following the designated treatments, THP‐1 cells were lysed in a buffer comprising 0.5% Triton X‐100, 50 mM Tris‐HCl, 150 mM NaCl, 10% glycerol, and protease inhibitors. The pellet obtained after centrifugation was resuspended in sample buffer (0.5× TBE, 10% glycerol, 2% SDS, 0.0025% bromophenol blue) for electrophoretic separation. Separation was carried out on a 1.5% agarose gel in running buffer (1× TAE, 0.1% SDS) for 2 h. Proteins were subsequently transferred to PVDF membranes for immunoblotting analysis.

### 4.12 HEK293T Transfection

HEK293T cells were transfected with plasmid DNA using linear polyethylenimine (PEI) at a 1:2 ratio, seeded into 6‐well plates, and cultured overnight at 37°C. CeeNU was administered 8 h after transfection, and samples were harvested 24 h post‐transfection.

### 4.13 Cytokine Detection via ELISA

The concentrations of mouse IL‐1β, IL‐6, TNF‐α, and human IL‐1β in cell culture supernatants, footpad tissue cultures, colon homogenates, and serum were quantified using commercial ELISA kits (Bioworld), following the manufacturer's protocols.

### 4.14 DARTS

BMDMs were plated in 6 cm dishes and cultured overnight. After replacing the medium with fresh DMEM, cells were stimulated with LPS for 4 h and lysed in NP‐40 buffer. The lysates were cleared by centrifugation (12,000 × g, 10 min, 4°C). After determining protein concentration by BCA assay, 80 µg aliquots were incubated with CeeNU overnight at 4°C. These samples were then treated with pronase (2–10 µg/mL; Sigma, PRON‐RO) for 30 min at room temperature. The reaction was quenched by adding 20× PMSF and placing the samples on ice for 10 min. Finally, 5× SDS‐PAGE loading buffer was added to adjust to a 1× concentration, and proteins were analyzed by immunoblotting.

### 4.15 CETSA

Cells were primed with LPS for 4 h, treated with CeeNU for 1.5 h, and then stimulated with nigericin for 1 h. Following this treatment regimen, the cells were detached using 0.25% trypsin, collected by centrifugation (13,000 rpm, 5 min), washed twice with PBS, and resuspended in PBS containing protease inhibitors. The cell suspension was divided into six equal aliquots and subjected to heating at 37°C, 43°C, 49°C, 55°C, 61°C, and 67°C for 3 min, followed by lysis through repeated freeze‐thaw cycles in liquid nitrogen. Supernatants were collected after centrifugation at 13,000 rpm for 30 min and analyzed via Western blotting.

### 4.16 SPR Analysis

For SPR analysis, purified human NLRP3 (Mabnus, GS11683) was captured onto a CM5 sensor chip, using a blank channel as a control. CeeNU, serially diluted in PBS to the indicated concentrations, was injected across the chip. All buffers were prepared with ultrapure water and 0.22 µm filtered. The assay was run at 25 ± 1°C after system priming with running buffer. The dissociation constant (KD) was derived from steady‐state fitting using a BIAcore T200 instrument (GE Healthcare).

### 4.17 Molecular Docking

NLRP3 protein structure (PDB ID: 6NPY) was obtained from the Protein Data Bank. The crystal structure was preprocessed with Pymol 2.3.0 to remove crystallographic water molecules and bound ligands. The prepared protein was then loaded into AutoDock for hydrogen addition, charge computation, partial charge assignment, and atom type definition. Docking simulations were carried out with the following parameters: grid center set at *x* = 87.015, *y* = 95.575, *z* = 91.600; a 60 × 60 × 60 Å grid box with 0.375 Å spacing; exhaustiveness = 10; and remaining parameters at default settings. The resulting docking poses were subsequently evaluated.

### 4.18 NLRP3 Reconstitution

NLRP3*^−/−^* BMDMs were seeded into 6‐well plates and transduced at a multiplicity of infection (MOI) of 50 with lentiviruses overexpressing either WT or mutant (R335A/R335K) NLRP3. After 8 h, the culture medium was refreshed with complete DMEM containing 10% FBS. Seventy‐two hours post‐transduction, cells were subjected to inflammasome activation stimuli and CeeNU treatment.

### 4.19 Mice

The WT mice were purchased from the Model Animal Research Center of Nanjing University. The *GSDMD^−/−^* mice were purchased from Cyagen. The *NLRP3^−/−^* mice were purchased from Hangzhou Ziyuan Laboratory Animal Technology Co. Ltd. All the mice were on a C57BL/6 background and housed in an SPF animal facility at Nanjing Drum Tower Hospital. Age‐ and gender‐matched littermates were used for the experiment.

### 4.20 Mouse Models

#### 4.20.1 MSU‐Induced Murine Peritonitis Model

Male C57BL/6 mice (8–10 weeks old) were administered CeeNU (10 mg/kg) via intraperitoneal injection twice at 12‐h intervals. One hour following the second injection, mice received an intraperitoneal injection of MSU solution (1 mg in 0.2 mL PBS). After 6 h, peritoneal lavage was performed using 5 mL of ice‐cold PBS containing 0.5 mM EDTA. The lavage fluid was centrifuged at 2000 rpm for 5 min; cells were resuspended for flow cytometry, and the supernatant was retained for ELISA.

#### 4.20.2 MSU‐Induced Murine Gouty Arthritis Model

Treatment consisted of two intraperitoneal injections of CeeNU (10 mg/kg) administered to 8–10‐week‐old male C57BL/6 mice, separated by a 12‐h interval. One hour after the final administration, MSU solution (0.8 mg in 40 µL PBS) was injected into the footpad. Footpad thickness was measured hourly for 6 h using a caliper and documented photographically. At the 6‐h time point, foot joint tissues were collected for caspase‐1 activity assessment using a commercial assay kit (Beyotime, C1102), IL‐1β quantification by ELISA, and histopathological evaluation via H&E staining.

#### 4.20.3 LPS‐Induced Sepsis Murine Model

Female C57BL/6 mice (8–10 weeks old) were subjected to pretreatment with two intraperitoneal injections of CeeNU, administered 12 h apart. One hour after the second LPS injection, an additional dose was delivered intraperitoneally. Twelve hours following the final administration, whole blood was collected for flow cytometry, serum was isolated for ELISA, and lung tissues were harvested for H&E staining.

#### 4.20.4 MOG_35–55_‐Induced EAE Murine Model

To establish the EAE model, 8‐week‐old female C57BL/6 mice received subcutaneous injections on the dorsum with an emulsion comprising MOG_35–55_ (250 µg/mouse), *Mycobacterium tuberculosis* (400 µg/mouse), and complete Freund's adjuvant (CFA; 50 µL/mouse). Pertussis toxin (PTX; 500 ng/mouse) in PBS was administered intraperitoneally at 0 and 48 h. Disease progression was monitored daily using a standardized clinical scoring system: 0.5 for partial tail limpness; 1 for complete tail paralysis; 2 for hindlimb weakness; 3 for hindlimb paralysis; 4 for hindlimb paralysis accompanied by forelimb impairment; and 5 for a moribund state or death. At the disease peak, mice were euthanized and subjected to cardiac perfusion with PBS. Following harvest, brain tissues were processed for flow cytometry, while spinal cord tissues were respectively subjected to histological examination and real‐time PCR analysis.

### 4.21 Caspase‐1 Activity Assay

Footpad tissues from euthanized arthritis model mice were collected and incubated in serum‐free DMEM for 1 h. After incubation, the supernatant was isolated via centrifugation. Caspase‐1 activity in the supernatant was measured using a commercial assay kit, strictly following the manufacturer's instructions.

### 4.22 NLRP3 ATPase Activity Assay

Briefly, human NLRP3‐expressing HEK293T cells were treated with CeeNU (40 min); subsequently, ultrapure ATP was added and incubated at 37°C for 40 min. ATP‐to‐ADP conversion was quantified based on luminescent signal detection.

### 4.23 Histological Analysis

Spinal cord sections (5 µm thickness) were used in this study. To assess inflammation and demyelination, H&E and LFB staining were employed for histopathological evaluation, respectively. A double‐blind histological scoring system was employed: 0, no inflammation or demyelination; 1, minimal inflammatory infiltration or demyelination; 2, moderate involvement; 3, severe and extensive inflammatory infiltration or demyelination.

### 4.24 Real‐Time PCR

Total RNA isolation was performed with TRIzol, followed by cDNA synthesis using the GoScript Reverse Transcription System (Vazyme). Quantitative PCR analysis was then carried out on an iQ5 real‐time PCR system (Bio‐Rad) with SYBR Green Master Mix (Vazyme). Relative expression was normalized to GAPDH and calculated via the 2^−ΔΔCt^ method. The primer sequences utilized in this study were shown in Table S3.

### 4.25 Statistical Analysis

Statistical analysis was performed with GraphPad Prism 8.0, with data presented as mean ± SEM. Clinical scores were assessed by two‐way multiple‐range ANOVA; two‐group comparisons used an unpaired *t*‐test, and multiple‐group comparisons employed two‐way ANOVA (^*^ *p* < 0.05, ^**^ *p* < 0.01, ^***^ *p* < 0.001). It is pertinent to mention that all data were obtained from at least three independent experiments.

## Author Contributions

Y.X. and S.‐L.J. designed the project. S.‐L.J., P.C., H.T., C.Z., Z.L., Y.W., X.C., L.Z., Z.L., Y.C., and X.B. performed the experiments and analyzed the data. S.‐L.J., P.C., and H.T. wrote the manuscript. Y.X. revised the manuscript. All authors have read and approved the final manuscript.

## Funding

This research was supported by the National Natural Science Foundation of China (82101417, 82071408, 81920108017, and 82130036).

## Ethics Statement

This study was conducted in accordance with the ARRIVE (Animal Research: Reporting of In Vivo Experiments) guidelines, and all animal procedures received prior approval from the Animal Care Committee of Nanjing Drum Tower Hospital (Approval No.: 2023AE01029).

## Conflicts of Interest

The authors declare no conflicts of interest.

## Supporting information




**Figure S1**: The optimal concentration of CeeNU on iBMDMs, BMDMs and THP‐1 cells.
**Figure S2**: CeeNU inhibits pyroptotic cell death in mouse macrophages and microglia.
**Figure S3**: CeeNU has no effect on ROS, potassium efflux, or DNA damage.
**Figure S4**: DARTS and Silver Stain analysis of proteins stability in LPS‐primed THP‐1 cell lysis treated with 50 µM CeeNU at different concentrations of pronase (0, 2, and 5µg/ml).
**Figure S5**: NLRP3 Arg335 is a conserved site among different species. Through amino acid sequence analysis and homology alignment, we found that NLRP3 Arg335 is a conserved site in both humans and mice.
**Figure S6**: CeeNU significantly alleviate LPS‐induced septic shock.
**Table S1**: The list of 2747 FDA‐approved compound library.
**Table S2**: Mass spectrometry of CeeNU‐interacting protein.
**Table S3**: Oligonucleotide primers used in reverse transcription real‐time quantitative PCR

## Data Availability

Data supporting this study are available from the corresponding author upon reasonable request.
