# Supplementary material for: High‐Throughput Screening Reveals That CeeNU Acts as a New NLRP3 Inflammasome Inhibitor
Source: MedComm (2020). 2026 Apr 23;7(5):e70695. doi: 10.1002/mco2.70695 (PMC13106880; doi:10.1002/mco2.70695)
Supplement: Supplementary file 1 — Figure S1: The optimal concentration of CeeNU on iBMDMs, BMDMs and THP‐1 cells. Figure S2: CeeNU inhibits pyroptotic cell death in mouse macrophages and microglia. Figure S3: CeeNU has no effect on ROS, potassium efflux, or DNA damage. Figure S4: DARTS and Silver Stain analysis of proteins stability in LPS‐primed THP‐1 cell lysis treated with 50 µM CeeNU at different concentrations of pronase (0, 2, and 5µg/ml). Figure S5: NLRP3 Arg335 is a conserved site among different species. Through amino acid sequence analysis and homology alignment, we found that NLRP3 Arg335 is a conserved site in both humans and mice. Figure S6: CeeNU significantly alleviate LPS‐induced septic shock. Table S1: The list of 2747 FDA‐approved compound library. Table S2: Mass spectrometry of CeeNU‐interacting protein. Table S3: Oligonucleotide primers used in reverse transcription real‐time quantitative PCR [file MCO2-7-e70695-s001.docx]

**Supplementary Materials for:**

**High-throughput screening reveals that CeeNU acts as a new NLRP3 inhibitor**

Sen-Lin Ji ^1,2,3,4,#^, Peipei Chen ^1,2,3,4,#^, Huaiping Tang ^1,2,3,4,#^, Chao Zhou ^1,2,3,4^, Zihao Li^1,2,3,4^, Yunshu Wang^1,2,3,4^, Xiang Cao^1,2,3,4^, Liwen Zhu^1,2,3,4^, Xinyu Bao^1,2,3,4^, Zhuo Liu^1,2,3,4^, Yan Chen^1,2,3,4^, Yun Xu^1,2,3,4,^*

1 Department of Neurology, Nanjing Drum Tower Hospital, Affiliated Hospital of Medical School, Nanjing University, Nanjing, China

2 State Key Laboratory of Pharmaceutical Biotechnology and Institute of Translational Medicine for Brain Critical Diseases, Nanjing University, Nanjing, China

3 Jiangsu Key Laboratory for Molecular Medicine, Medical School of Nanjing University, Nanjing, China

4 Nanjing Neurology Clinical Medical Center, Nanjing, China

**∗Correspondence**

Yun Xu, Department of Neurology, Nanjing Drum Tower Hospital, Affiliated Hospital of Medical School, Nanjing University, Nanjing, 210008, China.

E-mail: xuyun20042001@aliyun.com

^#^Sen-Lin Ji, Peipei Chen, and Huaiping Tang contributed equally to the work.


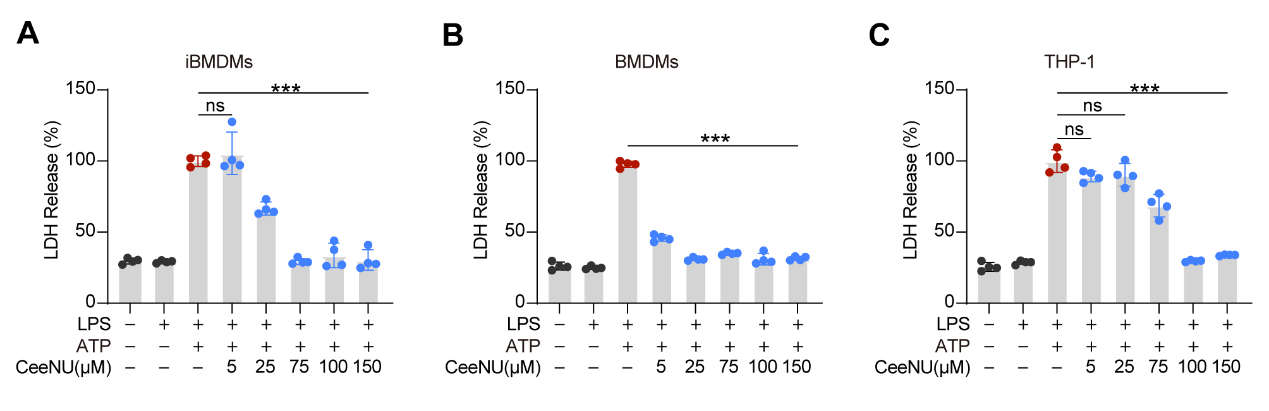


**Figure S1. The optimal concentration of CeeNU on iBMDMs, BMDMs and THP-1 cells.**

LPS-primed iBMDMs, BMDMs or THP-1 treated with different dose of CeeNU for 1 h before stimulation with ATP for 30 min. (A) LDH release from iBMDMs supernatant stimulated with LPS+ATP. (B)LDH release from BMDMs supernatant stimulated with LPS+ATP. (C) LDH release from THP-1 supernatant stimulated with LPS+ATP. *p < 0.05, **p < 0.01, ***p < 0.001. Data are mean±SEM. Blots are representative of a minimum of 4 independent experiments.


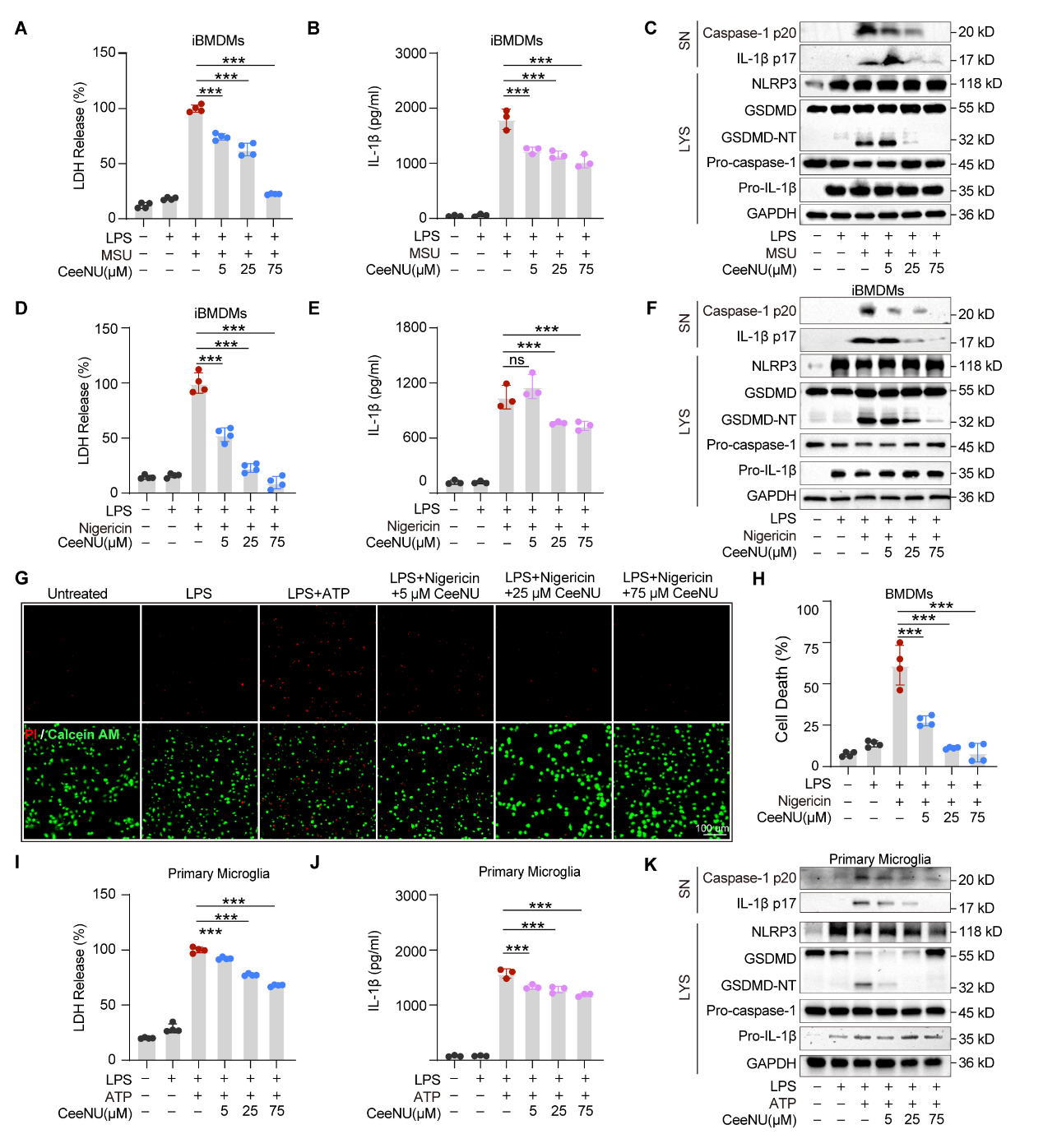


**Figure S2**. **CeeNU inhibits pyroptotic cell death in mouse macrophages and microglia.**

LPS-primed iBMDM or microglia treated with CeeNU for 1h before stimulation with ATP for 30 min or Nigericin for 1h. (**A and D**) LDH release from iBMDMs supernatant stimulated with LPS+MSU, or LPS+Nigericin (**D**). (**B and E**) ELISA of IL-1β in the supernatant of LPS+ MSU stimulated iBMDMs, or LPS+Nigericin (**E**). (**C** and **F**) Western blotting analysis of cleaved IL-1β p17 and caspase-1 p20 levels in cell supernanant and pro-IL-1β, pro-caspase-1, NLRP3, GSDMD, GSDMD-NT and GAPDH in cell lysates of BMDMs stimulated with LPS+MSU (**C**), or LPS+Nigericin (**F**). (**G**) iBMDMs were treated with LPS for 3 hours plus CeeNU for 1 hours, followed by stimulation with Nigericin1 h, then stained with cells were stained with 2 μg/mL PI (red; staining dying cells) and 3 μg/mL calcein AM (green; staining all cells) for 15 min, and then observed by fluorescence microscopy. Bright-field images are also shown in merged ones. Scale bars, 200 μm. (**H**) PI-positive cells in 4 randomly chosen fields were quantified. The percentage of lytic cell death is defined as the ratio of PI-positive cells relative to all (revealed by calcein AM). (**I**) LDH release from microglia supernatant stimulated with LPS+ATP. (**J**) ELISA of IL-1β in the supernatant of LPS+ ATP stimulated microglia. (**K**) Western blotting analysis of cleaved IL-1β p17 and caspase-1 p20 levels in cell supernanant and pro-IL-1β, pro-caspase-1, NLRP3, GSDMD, GSDMD-NT and GAPDH in cell lysates of BMDMs stimulated with LPS+ATP. *p < 0. 05, **p < 0. 01, ***p < 0. 001. Data are mean ± SEM. Blots are representative of a minimum of 3 independent experiments.


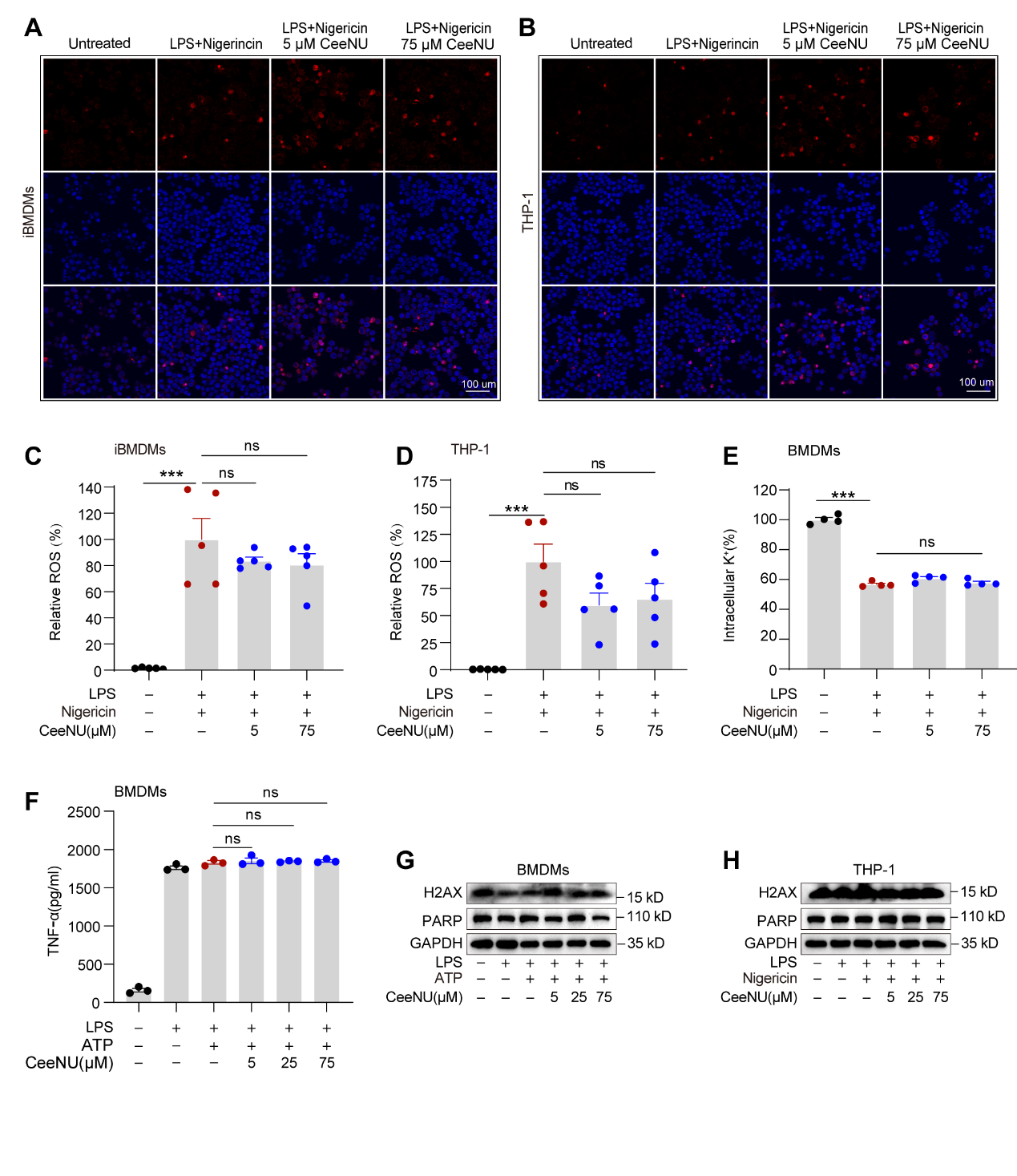


**Figure S3**. **CeeNU has no effect on ROS, potassium efflux, or DNA damage.**

(**A** and **B**) Confocal microscopy analysis of LPS-primed iBMDMs(A) and THP-1(B) cells treated with 5 and 75 μM CeeNU and then stimulated with 5 μM nigericin for 1 h, followed by staining with DAPI and mitosox. (**C** and **D**) Qualification analysis of ROS in iBMDMs(C) and THP-1(D). (**E**) Qualification analysis of intracellular potassium level in LPS-primed BMDMs treated with various doses of CeeNU and then stimulated with 5 μM nigericin for 1 h. (**F**) ELISA of TNF-α in the supernatant of LPS+ MSU stimulated BMDMs. (**G**) Immunoblot analysis of phosphorylated histone H2AX (γH2AX) in in LPS-primed BMDMs treated with various doses of CeeNU and then stimulated with 5 μM nigericin for 1 h. *p < 0. 05, **p < 0. 01, ***p < 0. 001. Data are mean ± SEM. Blots are representative of a minimum of 3 independent experiments.


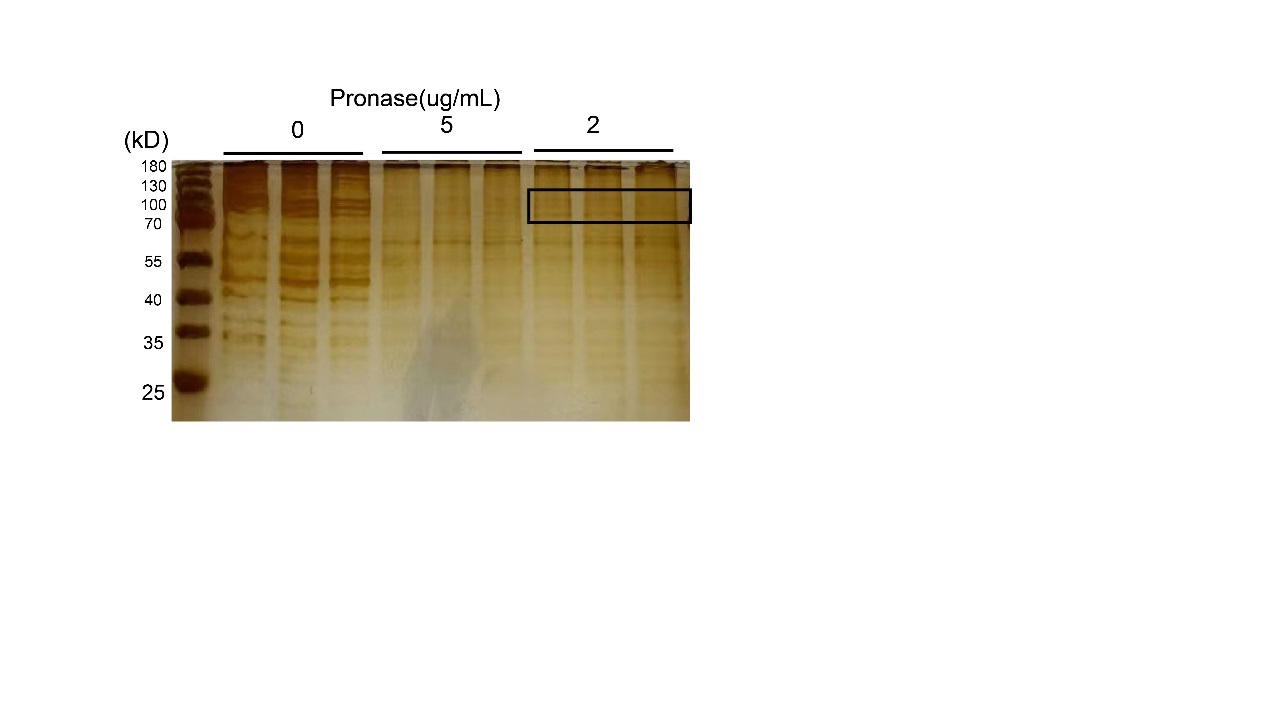


**Figure S4**. DARTS and Silver Stain analysis of proteins stability in LPS-primed THP-1 cell lysis treated with 50 μM CeeNU at different concentrations of pronase (0, 2, and 5μg/ml).


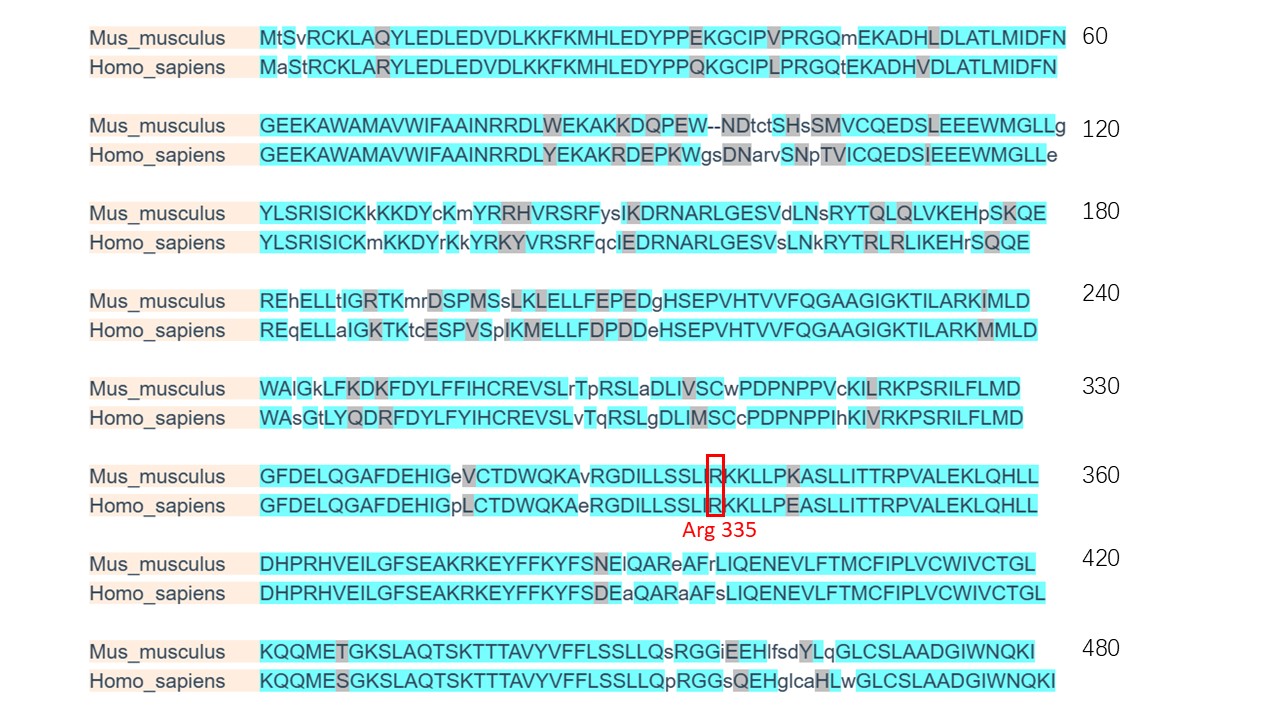


**Figure S5**. NLRP3 Arg335 is a conserved site among different species. Through amino acid sequence analysis and homology alignment, we found that NLRP3 Arg335 is a conserved site in both humans and mice.


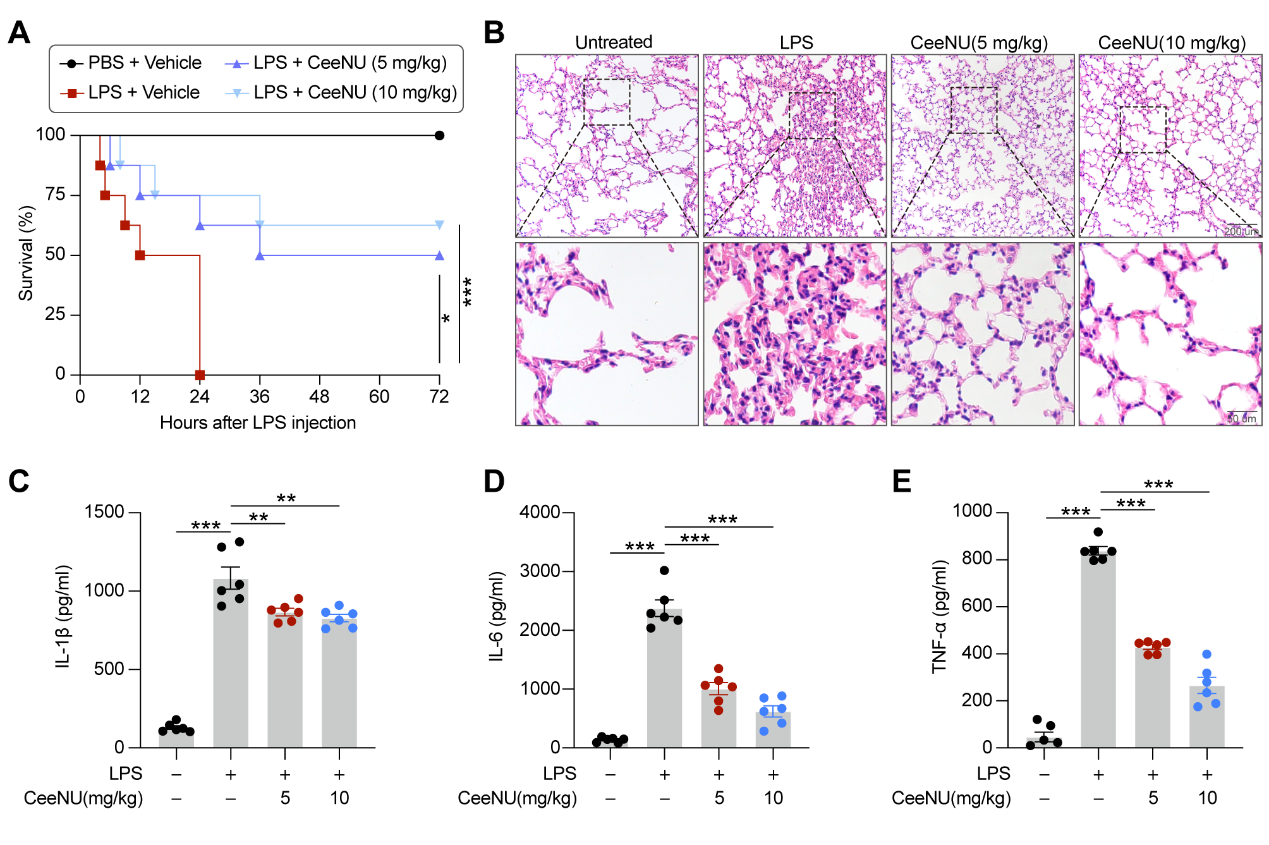


**Figure S6. CeeNU significantly alleviate LPS-induced septic shock.**

Mice were pre-injected with CeeNU (or vehicle control) twice (-12h and -1h) before intraperitoneal injection of LPS (15 mg/kg) for 12 h. (**A**) The survival percentage of mice injected with/without CeeNU. (**B**) Hematoxylin and eosin staining of the lung tissue. ELISA of IL-1β (**C**), IL-6 (**D**) and TNF-α (**E**) levels in serum. *p < 0. 05, **p < 0. 01, ***p < 0. 001. Data are mean ± SEM. Blots are representative of a minimum of 6 independent experiments.

**Table S1. The list of 2747 FDA-approved compound library.**

|  | **Cat** | **Name** | **Plate Location** | **Rack Number** |
| --- | --- | --- | --- | --- |
| 1 | S1005 | Axitinib | a2 | L1300-01 |
| 2 | S1007 | Roxadustat (FG-4592) | b2 | L1300-01 |
| 3 | S1010 | Nintedanib (BIBF 1120) | c2 | L1300-01 |
| 4 | S1011 | Afatinib (BIBW2992) | d2 | L1300-01 |
| 5 | S1013 | Bortezomib (PS-341) | e2 | L1300-01 |
| 6 | S1014 | Bosutinib (SKI-606) | f2 | L1300-01 |
| 7 | S1021 | Dasatinib | g2 | L1300-01 |
| 8 | S1022 | Ridaforolimus (Deforolimus, MK-8669) | h2 | L1300-01 |
| 9 | S1025 | Gefitinib (ZD1839) | a3 | L1300-01 |
| 10 | S1026 | Imatinib Mesylate (STI571) | b3 | L1300-01 |
| 11 | S1028 | Lapatinib (GW-572016) Ditosylate | c3 | L1300-01 |
| 12 | S1029 | Lenalidomide (CC-5013) | d3 | L1300-01 |
| 13 | S1030 | Panobinostat (LBH589) | e3 | L1300-01 |
| 14 | S1033 | Nilotinib (AMN-107) | f3 | L1300-01 |
| 15 | S1035 | Pazopanib HCl (GW786034 HCl) | g3 | L1300-01 |
| 16 | S1039 | Rapamycin (Sirolimus) | h3 | L1300-01 |
| 17 | S1040 | Sorafenib Tosylate | a4 | L1300-01 |
| 18 | S1042 | Sunitinib Malate | b4 | L1300-01 |
| 19 | S1044 | Temsirolimus (CCI-779, NSC 683864) | c4 | L1300-01 |
| 20 | S1047 | Vorinostat (SAHA, MK0683) | d4 | L1300-01 |
| 21 | S1053 | Entinostat (MS-275) | e4 | L1300-01 |
| 22 | S1055 | Enzastaurin (LY317615) | f4 | L1300-01 |
| 23 | S1060 | Olaparib (AZD2281, Ku-0059436) | g4 | L1300-01 |
| 24 | S1064 | Masitinib (AB1010) | h4 | L1300-01 |
| 25 | S1068 | Crizotinib (PF-02341066) | a5 | L1300-01 |
| 26 | S1082 | Vismodegib (GDC-0449) | b5 | L1300-01 |
| 27 | S1085 | Belinostat (PXD101) | c5 | L1300-01 |
| 28 | S1098 | Rucaparib (AG-014699,PF-01367338) phosphate | d5 | L1300-01 |
| 29 | S1119 | Cabozantinib (XL184, BMS-907351) | e5 | L1300-01 |
| 30 | S1120 | Everolimus (RAD001) | f5 | L1300-01 |
| 31 | S1137 | Malotilate | g5 | L1300-01 |
| 32 | S1144 | Ivacaftor (VX-770) | h5 | L1300-01 |
| 33 | S1148 | Docetaxel | a6 | L1300-01 |
| 34 | S1150 | Paclitaxel | b6 | L1300-01 |
| 35 | S1156 | Capecitabine | c6 | L1300-01 |
| 36 | S1164 | Lenvatinib (E7080) | d6 | L1300-01 |
| 37 | S1168 | Valproic acid sodium salt (Sodium valproate) | e6 | L1300-01 |
| 38 | S1178 | Regorafenib (BAY 73-4506) | f6 | L1300-01 |
| 39 | S1183 | Danoprevir (ITMN-191) | g6 | L1300-01 |
| 40 | S1185 | Ritonavir | h6 | L1300-01 |
| 41 | S1188 | Anastrozole | a7 | L1300-01 |
| 42 | S1189 | Aprepitant | b7 | L1300-01 |
| 43 | S1190 | Bicalutamide | c7 | L1300-01 |
| 44 | S1191 | Fulvestrant | d7 | L1300-01 |
| 45 | S1192 | Raltitrexed | e7 | L1300-01 |
| 46 | S1193 | Thalidomide | f7 | L1300-01 |
| 47 | S1196 | Exemestane | g7 | L1300-01 |
| 48 | S1197 | Finasteride | h7 | L1300-01 |
| 49 | S1199 | Cladribine | a8 | L1300-01 |
| 50 | S1200 | Decitabine | b8 | L1300-01 |
| 51 | S1202 | Dutasteride | c8 | L1300-01 |
| 52 | S1204 | Melatonin | d8 | L1300-01 |
| 53 | S1206 | Bisoprolol fumarate | e8 | L1300-01 |
| 54 | S1207 | Tivozanib (AV-951) | f8 | L1300-01 |
| 55 | S1208 | Doxorubicin (Adriamycin) HCl | g8 | L1300-01 |
| 56 | S1209 | Fluorouracil (5-Fluoracil, 5-FU) | h8 | L1300-01 |
| 57 | S1210 | Methotrexate | a9 | L1300-01 |
| 58 | S1212 | Bendamustine HCl | b9 | L1300-01 |
| 59 | S1223 | Epirubicin HCl | c9 | L1300-01 |
| 60 | S1225 | Etoposide | d9 | L1300-01 |
| 61 | S1227 | Raloxifene HCl | e9 | L1300-01 |
| 62 | S1229 | Fludarabine Phosphate | f9 | L1300-01 |
| 63 | S1231 | Topotecan HCl | g9 | L1300-01 |
| 64 | S1233 | 2-Methoxyestradiol (2-MeOE2) | h9 | L1300-01 |
| 65 | S1235 | Letrozole | a10 | L1300-01 |
| 66 | S1237 | Temozolomide | b10 | L1300-01 |
| 67 | S1238 | Tamoxifen | c10 | L1300-01 |
| 68 | S1241 | Vincristine sulfate | d10 | L1300-01 |
| 69 | S1243 | Agomelatine | e10 | L1300-01 |
| 70 | S1247 | Leflunomide | f10 | L1300-01 |
| 71 | S1250 | Enzalutamide (MDV3100) | g10 | L1300-01 |
| 72 | S1251 | Dienogest | h10 | L1300-01 |
| 73 | S1252 | Entecavir Hydrate | a11 | L1300-01 |
| 74 | S1255 | Nepafenac | b11 | L1300-01 |
| 75 | S1256 | Rufinamide | c11 | L1300-01 |
| 76 | S1257 | Posaconazole | d11 | L1300-01 |
| 77 | S1258 | Prasugrel | e11 | L1300-01 |
| 78 | S1259 | Ramelteon | f11 | L1300-01 |
| 79 | S1260 | Cinacalcet HCl | g11 | L1300-01 |
| 80 | S1261 | Celecoxib | h11 | L1300-01 |
| 81 | S1267 | Vemurafenib (PLX4032, RG7204) | a2 | L1300-02 |
| 82 | S1271 | Acarbose | b2 | L1300-02 |
| 83 | S1276 | Adapalene | c2 | L1300-02 |
| 84 | S1278 | Altretamine | d2 | L1300-02 |
| 85 | S1280 | Amisulpride | e2 | L1300-02 |
| 86 | S1281 | Aniracetam | f2 | L1300-02 |
| 87 | S1282 | Artemisinin | g2 | L1300-02 |
| 88 | S1283 | Asenapine maleate | h2 | L1300-02 |
| 89 | S1284 | Benazepril HCl | a3 | L1300-02 |
| 90 | S1286 | Budesonide | b3 | L1300-02 |
| 91 | S1287 | Bumetanide | c3 | L1300-02 |
| 92 | S1289 | Carmofur | d3 | L1300-02 |
| 93 | S1291 | Cetirizine DiHCl | e3 | L1300-02 |
| 94 | S1293 | Cilnidipine | f3 | L1300-02 |
| 95 | S1294 | Cilostazol | g3 | L1300-02 |
| 96 | S1299 | Floxuridine | h3 | L1300-02 |
| 97 | S1300 | Tegafur (FT-207, NSC 148958) | a4 | L1300-02 |
| 98 | S1302 | Ifosfamide | b4 | L1300-02 |
| 99 | S1304 | Megestrol Acetate | c4 | L1300-02 |
| 100 | S1305 | Mercaptopurine (6-MP) | d4 | L1300-02 |
| 101 | S1312 | Streptozotocin (STZ) | e4 | L1300-02 |
| 102 | S1319 | Costunolide | f4 | L1300-02 |
| 103 | S1322 | Dexamethasone (DHAP) | g4 | L1300-02 |
| 104 | S1324 | Doxazosin Mesylate | h4 | L1300-02 |
| 105 | S1326 | Edaravone | a5 | L1300-02 |
| 106 | S1328 | Etodolac | b5 | L1300-02 |
| 107 | S1329 | Etomidate | c5 | L1300-02 |
| 108 | S1330 | Felbamate | d5 | L1300-02 |
| 109 | S1331 | Fluconazole | e5 | L1300-02 |
| 110 | S1332 | Flumazenil | f5 | L1300-02 |
| 111 | S1333 | Fluoxetine HCl | g5 | L1300-02 |
| 112 | S1334 | Flupirtine maleate | h5 | L1300-02 |
| 113 | S1336 | Fluvoxamine maleate | a6 | L1300-02 |
| 114 | S1340 | Gatifloxacin | b6 | L1300-02 |
| 115 | S1342 | Genistein | c6 | L1300-02 |
| 116 | S1344 | Glimepiride | d6 | L1300-02 |
| 117 | S1351 | Ivermectin | e6 | L1300-02 |
| 118 | S1354 | Lansoprazole | f6 | L1300-02 |
| 119 | S1356 | Levetiracetam | g6 | L1300-02 |
| 120 | S1357 | Lidocaine | h6 | L1300-02 |
| 121 | S1358 | Loratadine | a7 | L1300-02 |
| 122 | S1359 | Losartan Potassium (DuP 753) | b7 | L1300-02 |
| 123 | S1367 | Amonafide | c7 | L1300-02 |
| 124 | S1368 | Acitretin | d7 | L1300-02 |
| 125 | S1373 | Daptomycin | e7 | L1300-02 |
| 126 | S1374 | Doripenem Hydrate | f7 | L1300-02 |
| 127 | S1376 | Gestodene | g7 | L1300-02 |
| 128 | S1377 | Drospirenone | h7 | L1300-02 |
| 129 | S1378 | Ruxolitinib (INCB018424) | a8 | L1300-02 |
| 130 | S1379 | Isotretinoin | b8 | L1300-02 |
| 131 | S1380 | Lopinavir | c8 | L1300-02 |
| 132 | S1381 | Meropenem | d8 | L1300-02 |
| 133 | S1382 | Mianserin HCl | e8 | L1300-02 |
| 134 | S1385 | Mosapride Citrate | f8 | L1300-02 |
| 135 | S1386 | Nafamostat Mesylate | g8 | L1300-02 |
| 136 | S1387 | Naftopidil DiHCl | h8 | L1300-02 |
| 137 | S1389 | Omeprazole | a9 | L1300-02 |
| 138 | S1390 | Ondansetron HCl | b9 | L1300-02 |
| 139 | S1391 | Oxcarbazepine | c9 | L1300-02 |
| 140 | S1393 | Pirarubicin | d9 | L1300-02 |
| 141 | S1394 | Pizotifen Malate | e9 | L1300-02 |
| 142 | S1396 | Resveratrol | f9 | L1300-02 |
| 143 | S1397 | Rocuronium Bromide | g9 | L1300-02 |
| 144 | S1398 | Stavudine (d4T) | h9 | L1300-02 |
| 145 | S1400 | Tenofovir Disoproxil Fumarate | a10 | L1300-02 |
| 146 | S1401 | Tenofovir | b10 | L1300-02 |
| 147 | S1403 | Tigecycline | c10 | L1300-02 |
| 148 | S1404 | Trilostane | d10 | L1300-02 |
| 149 | S1405 | Vecuronium Bromide | e10 | L1300-02 |
| 150 | S1407 | Bimatoprost | f10 | L1300-02 |
| 151 | S1408 | Linezolid | g10 | L1300-02 |
| 152 | S1409 | Alfuzosin HCl | h10 | L1300-02 |
| 153 | S1415 | Clopidogrel | a11 | L1300-02 |
| 154 | S1425 | Ranolazine 2HCl | b11 | L1300-02 |
| 155 | S1426 | Repaglinide | c11 | L1300-02 |
| 156 | S1430 | Rolipram | d11 | L1300-02 |
| 157 | S1431 | Sildenafil Citrate | e11 | L1300-02 |
| 158 | S1432 | Sumatriptan Succinate | f11 | L1300-02 |
| 159 | S1435 | Tamsulosin | g11 | L1300-02 |
| 160 | S1436 | Tianeptine sodium | h11 | L1300-02 |
| 161 | S1437 | Tizanidine HCl | a2 | L1300-03 |
| 162 | S1438 | Topiramate | b2 | L1300-03 |
| 163 | S1439 | Tranilast | c2 | L1300-03 |
| 164 | S1441 | Venlafaxine HCl | d2 | L1300-03 |
| 165 | S1442 | Voriconazole | e2 | L1300-03 |
| 166 | S1443 | Zileuton | f2 | L1300-03 |
| 167 | S1444 | Ziprasidone HCl | g2 | L1300-03 |
| 168 | S1445 | Zonisamide | h2 | L1300-03 |
| 169 | S1453 | Tipifarnib | a3 | L1300-03 |
| 170 | S1457 | Atazanavir Sulfate | b3 | L1300-03 |
| 171 | S1458 | VX-745 | c3 | L1300-03 |
| 172 | S1465 | Moxifloxacin HCl | d3 | L1300-03 |
| 173 | S1466 | Calcitriol | e3 | L1300-03 |
| 174 | S1467 | Doxercalciferol | f3 | L1300-03 |
| 175 | S1468 | Alfacalcidol | g3 | L1300-03 |
| 176 | S1472 | Safinamide Mesylate | h3 | L1300-03 |
| 177 | S1482 | Daclatasvir (BMS-790052) | a4 | L1300-03 |
| 178 | S1483 | Iloperidone | b4 | L1300-03 |
| 179 | S1485 | HMN-214 | c4 | L1300-03 |
| 180 | S1488 | Naratriptan HCl | d4 | L1300-03 |
| 181 | S1490 | Ponatinib (AP24534) | e4 | L1300-03 |
| 182 | S1491 | Fludarabine | f4 | L1300-03 |
| 183 | S1497 | Pralatrexate | g4 | L1300-03 |
| 184 | S1499 | Cefaclor | h4 | L1300-03 |
| 185 | S1500 | Betamethasone | a5 | L1300-03 |
| 186 | S1501 | Mycophenolate Mofetil | b5 | L1300-03 |
| 187 | S1504 | Dyphylline | c5 | L1300-03 |
| 188 | S1505 | Aztreonam | d5 | L1300-03 |
| 189 | S1508 | Alprostadil | e5 | L1300-03 |
| 190 | S1511 | Lactulose | f5 | L1300-03 |
| 191 | S1512 | Tadalafil | g5 | L1300-03 |
| 192 | S1514 | Cyclosporine | h5 | L1300-03 |
| 193 | S1517 | Natamycin | a6 | L1300-03 |
| 194 | S1538 | Telaprevir (VX-950) | b6 | L1300-03 |
| 195 | S1540 | Saxagliptin | c6 | L1300-03 |
| 196 | S1547 | Febuxostat | d6 | L1300-03 |
| 197 | S1548 | Dapagliflozin | e6 | L1300-03 |
| 198 | S1549 | Nebivolol HCl | f6 | L1300-03 |
| 199 | S1550 | Pimobendan | g6 | L1300-03 |
| 200 | S1565 | VX-809 (Lumacaftor) | h6 | L1300-03 |
| 201 | S1567 | Pomalidomide | a7 | L1300-03 |
| 202 | S1569 | Tazarotene | b7 | L1300-03 |
| 203 | S1573 | Fasudil (HA-1077) HCl | c7 | L1300-03 |
| 204 | S1576 | Sulfasalazine | d7 | L1300-03 |
| 205 | S1578 | Candesartan | e7 | L1300-03 |
| 206 | S1593 | Apixaban | f7 | L1300-03 |
| 207 | S1601 | Reserpine | g7 | L1300-03 |
| 208 | S1603 | Furosemide | h7 | L1300-03 |
| 209 | S1604 | Olmesartan Medoxomil | a8 | L1300-03 |
| 210 | S1605 | Cefdinir | b8 | L1300-03 |
| 211 | S1606 | Clotrimazole | c8 | L1300-03 |
| 212 | S1607 | Rizatriptan Benzoate | d8 | L1300-03 |
| 213 | S1608 | Pyridostigmine Bromide | e8 | L1300-03 |
| 214 | S1609 | Methimazole | f8 | L1300-03 |
| 215 | S1610 | Metolazone | g8 | L1300-03 |
| 216 | S1611 | Cefoperazone | h8 | L1300-03 |
| 217 | S1613 | Silodosin | a9 | L1300-03 |
| 218 | S1614 | Riluzole | b9 | L1300-03 |
| 219 | S1618 | Sulfameter | c9 | L1300-03 |
| 220 | S1619 | Prilocaine | d9 | L1300-03 |
| 221 | S1620 | Darunavir Ethanolate | e9 | L1300-03 |
| 222 | S1622 | Prednisone | f9 | L1300-03 |
| 223 | S1623 | Acetylcysteine | g9 | L1300-03 |
| 224 | S1625 | Ethinyl Estradiol | h9 | L1300-03 |
| 225 | S1626 | Naproxen Sodium | a10 | L1300-03 |
| 226 | S1627 | Nitazoxanide | b10 | L1300-03 |
| 227 | S1628 | Triamcinolone Acetonide | c10 | L1300-03 |
| 228 | S1629 | Orlistat | d10 | L1300-03 |
| 229 | S1630 | Allopurinol | e10 | L1300-03 |
| 230 | S1631 | Allopurinol Sodium | f10 | L1300-03 |
| 231 | S1633 | Zafirlukast | g10 | L1300-03 |
| 232 | S1635 | Erythromycin | h10 | L1300-03 |
| 233 | S1636 | Amphotericin B | a11 | L1300-03 |
| 234 | S1638 | Ibuprofen | b11 | L1300-03 |
| 235 | S1639 | Amprenavir | c11 | L1300-03 |
| 236 | S1640 | Albendazole | d11 | L1300-03 |
| 237 | S1641 | Chlorothiazide | e11 | L1300-03 |
| 238 | S1643 | Ursodiol | f11 | L1300-03 |
| 239 | S1644 | Nitrofural | g11 | L1300-03 |
| 240 | S1645 | Ketoprofen | h11 | L1300-03 |
| 241 | S1646 | Ketorolac | a2 | L1300-04 |
| 242 | S1647 | Adenosine | b2 | L1300-04 |
| 243 | S1649 | Zolmitriptan | c2 | L1300-04 |
| 244 | S1651 | Telbivudine | d2 | L1300-04 |
| 245 | S1652 | Monobenzone | e2 | L1300-04 |
| 246 | S1653 | Tretinoin | f2 | L1300-04 |
| 247 | S1654 | Phenylbutazone | g2 | L1300-04 |
| 248 | S1655 | Ezetimibe | h2 | L1300-04 |
| 249 | S1657 | Enalaprilat Dihydrate | a3 | L1300-04 |
| 250 | S1658 | Dofetilide | b3 | L1300-04 |
| 251 | S1662 | Isradipine | c3 | L1300-04 |
| 252 | S1665 | Estrone | d3 | L1300-04 |
| 253 | S1666 | Flucytosine | e3 | L1300-04 |
| 254 | S1667 | Trichlormethiazide | f3 | L1300-04 |
| 255 | S1669 | Loteprednol etabonate | g3 | L1300-04 |
| 256 | S1672 | Aminoglutethimide | a4 | L1300-04 |
| 257 | S1673 | Aminophylline | b4 | L1300-04 |
| 258 | S1675 | Lubiprostone | c4 | L1300-04 |
| 259 | S1676 | Amorolfine HCl | d4 | L1300-04 |
| 260 | S1677 | Chloramphenicol | e4 | L1300-04 |
| 261 | S1679 | Flurbiprofen | f4 | L1300-04 |
| 262 | S1680 | Disulfiram | g4 | L1300-04 |
| 263 | S1681 | Mesalamine | h4 | L1300-04 |
| 264 | S1683 | Ipratropium Bromide | a5 | L1300-04 |
| 265 | S1685 | Sulfanilamide | b5 | L1300-04 |
| 266 | S1688 | Betamethasone Dipropionate | c5 | L1300-04 |
| 267 | S1689 | Meprednisone | d5 | L1300-04 |
| 268 | S1690 | Betamethasone Valerate | e5 | L1300-04 |
| 269 | S1691 | Praziquantel | f5 | L1300-04 |
| 270 | S1692 | Busulfan | g5 | L1300-04 |
| 271 | S1693 | Carbamazepine | h5 | L1300-04 |
| 272 | S1696 | Hydrocortisone | a6 | L1300-04 |
| 273 | S1701 | Desonide | b6 | L1300-04 |
| 274 | S1702 | Didanosine | c6 | L1300-04 |
| 275 | S1703 | Divalproex Sodium | d6 | L1300-04 |
| 276 | S1704 | Emtricitabine | e6 | L1300-04 |
| 277 | S1705 | Progesterone | f6 | L1300-04 |
| 278 | S1706 | Lamivudine | g6 | L1300-04 |
| 279 | S1709 | Estradiol | h6 | L1300-04 |
| 280 | S1712 | Deferasirox | a7 | L1300-04 |
| 281 | S1713 | Piroxicam | b7 | L1300-04 |
| 282 | S1714 | Gemcitabine | c7 | L1300-04 |
| 283 | S1715 | Glipizide | d7 | L1300-04 |
| 284 | S1716 | Glyburide (Glibenclamide) | e7 | L1300-04 |
| 285 | S1717 | Fomepizole | f7 | L1300-04 |
| 286 | S1718 | Adefovir Dipivoxil | g7 | L1300-04 |
| 287 | S1719 | Zalcitabine | h7 | L1300-04 |
| 288 | S1721 | Azathioprine | a8 | L1300-04 |
| 289 | S1723 | Indomethacin | b8 | L1300-04 |
| 290 | S1725 | Terbinafine | c8 | L1300-04 |
| 291 | S1727 | Levonorgestrel | d8 | L1300-04 |
| 292 | S1729 | Gemfibrozil | e8 | L1300-04 |
| 293 | S1730 | Indapamide | f8 | L1300-04 |
| 294 | S1732 | Mitotane | g8 | L1300-04 |
| 295 | S1733 | Methylprednisolone | h8 | L1300-04 |
| 296 | S1734 | Meloxicam | a9 | L1300-04 |
| 297 | S1735 | Mesna | b9 | L1300-04 |
| 298 | S1736 | Methocarbamol | c9 | L1300-04 |
| 299 | S1737 | Prednisolone | d9 | L1300-04 |
| 300 | S1738 | Telmisartan | e9 | L1300-04 |
| 301 | S1739 | Thiabendazole | f9 | L1300-04 |
| 302 | S1740 | Guaifenesin | g9 | L1300-04 |
| 303 | S1741 | Rifabutin | h9 | L1300-04 |
| 304 | S1742 | Nevirapine | a10 | L1300-04 |
| 305 | S1743 | NEXIUM (esomeprazole magnesium) | b10 | L1300-04 |
| 306 | S1744 | Nicotinic Acid | c10 | L1300-04 |
| 307 | S1747 | Nimodipine | d10 | L1300-04 |
| 308 | S1748 | Nisoldipine | e10 | L1300-04 |
| 309 | S1750 | Octocrylene | f10 | L1300-04 |
| 310 | S1754 | Oxybutynin | g10 | L1300-04 |
| 311 | S1756 | Enoxacin | h10 | L1300-04 |
| 312 | S1759 | Pitavastatin Calcium | a11 | L1300-04 |
| 313 | S1760 | Rifapentine | b11 | L1300-04 |
| 314 | S1761 | Suprofen | c11 | L1300-04 |
| 315 | S1762 | Pyrazinamide | d11 | L1300-04 |
| 316 | S1763 | Quetiapine Fumarate | e11 | L1300-04 |
| 317 | S1764 | Rifampin | f11 | L1300-04 |
| 318 | S1768 | Cefditoren Pivoxil | g11 | L1300-04 |
| 319 | S1770 | Sulfadiazine | h11 | L1300-04 |
| 320 | S1771 | Chlorprothixene | a2 | L1300-05 |
| 321 | S1773 | Oxytetracycline (Terramycin) | b2 | L1300-05 |
| 322 | S1774 | Thioguanine | c2 | L1300-05 |
| 323 | S1775 | Thiotepa | d2 | L1300-05 |
| 324 | S1776 | Toremifene Citrate | e2 | L1300-05 |
| 325 | S1777 | Ethionamide | f2 | L1300-05 |
| 326 | S1778 | Trifluridine | g2 | L1300-05 |
| 327 | S1782 | Azacitidine | h2 | L1300-05 |
| 328 | S1784 | Vidarabine | a3 | L1300-05 |
| 329 | S1786 | Verteporfin | b3 | L1300-05 |
| 330 | S1787 | Teniposide | c3 | L1300-05 |
| 331 | S1789 | Tetrabenazine (Xenazine) | d3 | L1300-05 |
| 332 | S1790 | Rifaximin | e3 | L1300-05 |
| 333 | S1792 | Simvastatin | f3 | L1300-05 |
| 334 | S1793 | Ramipril | g3 | L1300-05 |
| 335 | S1794 | Fenofibrate | h3 | L1300-05 |
| 336 | S1799 | Ranolazine | a4 | L1300-05 |
| 337 | S1801 | Ranitidine Hydrochloride | b4 | L1300-05 |
| 338 | S1806 | Acipimox | c4 | L1300-05 |
| 339 | S1807 | Aciclovir | d4 | L1300-05 |
| 340 | S1808 | Nifedipine | e4 | L1300-05 |
| 341 | S1811 | Amiloride HCl | f4 | L1300-05 |
| 342 | S1813 | Amlodipine Besylate | g4 | L1300-05 |
| 343 | S1816 | Chlorpheniramine Maleate | h4 | L1300-05 |
| 344 | S1823 | Fenoprofen Calcium | a5 | L1300-05 |
| 345 | S1825 | Erdosteine | b5 | L1300-05 |
| 346 | S1827 | Betaxolol HCl | c5 | L1300-05 |
| 347 | S1828 | Proparacaine HCl | d5 | L1300-05 |
| 348 | S1829 | Pranlukast | e5 | L1300-05 |
| 349 | S1830 | Oxfendazole | f5 | L1300-05 |
| 350 | S1831 | Carvedilol | g5 | L1300-05 |
| 351 | S1832 | Atracurium Besylate | h5 | L1300-05 |
| 352 | S1833 | Butoconazole nitrate | a6 | L1300-05 |
| 353 | S1835 | Azithromycin | b6 | L1300-05 |
| 354 | S1836 | Albendazole Oxide | c6 | L1300-05 |
| 355 | S1839 | Chloroxine | d6 | L1300-05 |
| 356 | S1840 | Lomustine | e6 | L1300-05 |
| 357 | S1843 | Chenodeoxycholic Acid | f6 | L1300-05 |
| 358 | S1845 | Cimetidine | g6 | L1300-05 |
| 359 | S1847 | Clemastine Fumarate | h6 | L1300-05 |
| 360 | S1848 | Curcumin | a7 | L1300-05 |
| 361 | S1849 | Daidzein | b7 | L1300-05 |
| 362 | S1854 | Bifonazole | c7 | L1300-05 |
| 363 | S1855 | Pefloxacin Mesylate | d7 | L1300-05 |
| 364 | S1856 | Metoprolol Tartrate | e7 | L1300-05 |
| 365 | S1858 | Dienestrol | f7 | L1300-05 |
| 366 | S1859 | Diethylstilbestrol | g7 | L1300-05 |
| 367 | S1865 | Diltiazem HCl | h7 | L1300-05 |
| 368 | S1866 | Diphenhydramine HCl | a8 | L1300-05 |
| 369 | S1869 | Dapoxetine HCl | b8 | L1300-05 |
| 370 | S1876 | Valaciclovir HCl | c8 | L1300-05 |
| 371 | S1878 | Ganciclovir | d8 | L1300-05 |
| 372 | S1880 | Roxatidine Acetate HCl | e8 | L1300-05 |
| 373 | S1881 | Protionamide | f8 | L1300-05 |
| 374 | S1883 | Idoxuridine | g8 | L1300-05 |
| 375 | S1884 | Sparfloxacin | h8 | L1300-05 |
| 376 | S1885 | Felodipine | a9 | L1300-05 |
| 377 | S1888 | Deflazacort | b9 | L1300-05 |
| 378 | S1890 | Nizatidine | c9 | L1300-05 |
| 379 | S1891 | Carbidopa | d9 | L1300-05 |
| 380 | S1894 | Valsartan | e9 | L1300-05 |
| 381 | S1895 | Dipyridamole | f9 | L1300-05 |
| 382 | S1896 | Hydroxyurea | g9 | L1300-05 |
| 383 | S1897 | Potassium Iodide | h9 | L1300-05 |
| 384 | S1898 | Tropisetron HCl | a10 | L1300-05 |
| 385 | S1899 | Nicotinamide (Vitamin B3) | b10 | L1300-05 |
| 386 | S1902 | Vitamin B12 | c10 | L1300-05 |
| 387 | S1903 | Diclofenac Sodium | d10 | L1300-05 |
| 388 | S1904 | Avobenzone | e10 | L1300-05 |
| 389 | S1905 | Amlodipine | f10 | L1300-05 |
| 390 | S1907 | Metronidazole | g10 | L1300-05 |
| 391 | S1908 | Flutamide | h10 | L1300-05 |
| 392 | S1909 | Fluvastatin Sodium | a11 | L1300-05 |
| 393 | S1910 | Tioconazole | b11 | L1300-05 |
| 394 | S1913 | Tropicamide | c11 | L1300-05 |
| 395 | S1914 | Pregnenolone | d11 | L1300-05 |
| 396 | S1915 | Sulfamethoxazole | e11 | L1300-05 |
| 397 | S1916 | Sulfisoxazole | f11 | L1300-05 |
| 398 | S1917 | Crystal Violet | g11 | L1300-05 |
| 399 | S1920 | Haloperidol | h11 | L1300-05 |
| 400 | S1921 | Phenindione | a2 | L1300-06 |
| 401 | S1933 | Triamcinolone | b2 | L1300-06 |
| 402 | S1934 | Nystatin (Fungicidin) | c2 | L1300-06 |
| 403 | S1937 | Isoniazid | d2 | L1300-06 |
| 404 | S1940 | Levofloxacin | e2 | L1300-06 |
| 405 | S1941 | Enalapril Maleate | f2 | L1300-06 |
| 406 | S1949 | Menadione | g2 | L1300-06 |
| 407 | S1950 | Metformin HCl | h2 | L1300-06 |
| 408 | S1952 | Methoxsalen | a3 | L1300-06 |
| 409 | S1956 | Miconazole Nitrate | b3 | L1300-06 |
| 410 | S1957 | Sulfamethizole | c3 | L1300-06 |
| 411 | S1958 | Sulbactam | d3 | L1300-06 |
| 412 | S1959 | Tolfenamic Acid | e3 | L1300-06 |
| 413 | S1960 | Pranoprofen | f3 | L1300-06 |
| 414 | S1964 | Rimantadine | g3 | L1300-06 |
| 415 | S1965 | Primidone | h3 | L1300-06 |
| 416 | S1969 | Nefiracetam | a4 | L1300-06 |
| 417 | S1971 | Nicorandil | b4 | L1300-06 |
| 418 | S1972 | Tamoxifen Citrate | c4 | L1300-06 |
| 419 | S1974 | Meglumine | d4 | L1300-06 |
| 420 | S1975 | Aripiprazole | e4 | L1300-06 |
| 421 | S1978 | Methscopolamine | f4 | L1300-06 |
| 422 | S1979 | Amiodarone HCl | g4 | L1300-06 |
| 423 | S1983 | Adenine HCl | h4 | L1300-06 |
| 424 | S1987 | Mometasone furoate | a5 | L1300-06 |
| 425 | S1988 | Propylthiouracil | b5 | L1300-06 |
| 426 | S1990 | Capsaicin(Vanilloid) | c5 | L1300-06 |
| 427 | S1992 | Fluticasone propionate | d5 | L1300-06 |
| 428 | S1994 | Lacidipine | e5 | L1300-06 |
| 429 | S2001 | Elvitegravir (GS-9137, JTK-303) | f5 | L1300-06 |
| 430 | S2003 | Maraviroc | g5 | L1300-06 |
| 431 | S2005 | Raltegravir (MK-0518) | h5 | L1300-06 |
| 432 | S2006 | Pyrimethamine | a6 | L1300-06 |
| 433 | S2007 | Sulindac | b6 | L1300-06 |
| 434 | S2011 | Pramipexole 2HCl Monohydrate | c6 | L1300-06 |
| 435 | S2015 | Suplatast Tosylate | d6 | L1300-06 |
| 436 | S2016 | Mirtazapine | e6 | L1300-06 |
| 437 | S2017 | Benidipine HCl | f6 | L1300-06 |
| 438 | S2020 | Formoterol Hemifumarate | g6 | L1300-06 |
| 439 | S2021 | Chlormezanone | h6 | L1300-06 |
| 440 | S1213 | Nelarabine | a7 | L1300-06 |
| 441 | S2024 | Ketotifen Fumarate | b7 | L1300-06 |
| 442 | S2025 | Urapidil HCl | c7 | L1300-06 |
| 443 | S2028 | Diclazuril | d7 | L1300-06 |
| 444 | S2029 | Uridine | e7 | L1300-06 |
| 445 | S2030 | Flunarizine 2HCl | f7 | L1300-06 |
| 446 | S2031 | Fenticonazole Nitrate | g7 | L1300-06 |
| 447 | S2032 | Rebamipide | h7 | L1300-06 |
| 448 | S2036 | Aspartame | a8 | L1300-06 |
| 449 | S2037 | Candesartan Cilexetil | b8 | L1300-06 |
| 450 | S2038 | Phentolamine Mesylate | c8 | L1300-06 |
| 451 | S2040 | Nimesulide | d8 | L1300-06 |
| 452 | S2041 | Dyclonine HCl | e8 | L1300-06 |
| 453 | S2042 | Cyproterone Acetate | f8 | L1300-06 |
| 454 | S2043 | Memantine HCl | g8 | L1300-06 |
| 455 | S2044 | Cyproheptadine HCl | h8 | L1300-06 |
| 456 | S2045 | Doxifluridine | a9 | L1300-06 |
| 457 | S2046 | Pioglitazone HCl | b9 | L1300-06 |
| 458 | S2051 | Captopril | c9 | L1300-06 |
| 459 | S2052 | Oxytetracycline Dihydrate | d9 | L1300-06 |
| 460 | S2053 | Cytidine | e9 | L1300-06 |
| 461 | S2054 | Orphenadrine Citrate | f9 | L1300-06 |
| 462 | S2055 | Gimeracil | g9 | L1300-06 |
| 463 | S2057 | Cyclophosphamide Monohydrate | h9 | L1300-06 |
| 464 | S2058 | Tolnaftate | a10 | L1300-06 |
| 465 | S2059 | Terazosin HCl Dihydrate | b10 | L1300-06 |
| 466 | S2060 | Bromhexine HCl | c10 | L1300-06 |
| 467 | S2061 | Lovastatin | d10 | L1300-06 |
| 468 | S2062 | Tiopronin | e10 | L1300-06 |
| 469 | S2064 | Balofloxacin | f10 | L1300-06 |
| 470 | S2065 | Lafutidine | g10 | L1300-06 |
| 471 | S2066 | Moxonidine | h10 | L1300-06 |
| 472 | S2067 | Ozagrel HCl | a11 | L1300-06 |
| 473 | S2069 | Argatroban | b11 | L1300-06 |
| 474 | S2071 | Prulifloxacin (NM441, AF 3013) | c11 | L1300-06 |
| 475 | S2075 | Rosiglitazone HCl | d11 | L1300-06 |
| 476 | S2077 | Atorvastatin Calcium | e11 | L1300-06 |
| 477 | S2078 | Famotidine | f11 | L1300-06 |
| 478 | S2079 | Moexipril HCl | g11 | L1300-06 |
| 479 | S2080 | Clevidipine Butyrate | h11 | L1300-06 |
| 480 | S2083 | Procaterol HCl | a2 | L1300-07 |
| 481 | S2084 | Duloxetine HCl | b2 | L1300-07 |
| 482 | S2085 | Trimebutine | c2 | L1300-07 |
| 483 | S2086 | Ivabradine HCl | d2 | L1300-07 |
| 484 | S2087 | Rivastigmine Tartrate | e2 | L1300-07 |
| 485 | S2090 | Dexmedetomidine HCl | f2 | L1300-07 |
| 486 | S2091 | Betaxolol | g2 | L1300-07 |
| 487 | S2092 | Detomidine HCl | h2 | L1300-07 |
| 488 | S2096 | Almotriptan Malate | a3 | L1300-07 |
| 489 | S2097 | Ambrisentan | b3 | L1300-07 |
| 490 | S2098 | Bexarotene | c3 | L1300-07 |
| 491 | S2099 | Temocapril HCl | d3 | L1300-07 |
| 492 | S2101 | Gabexate Mesylate | e3 | L1300-07 |
| 493 | S2102 | Rasagiline Mesylate | f3 | L1300-07 |
| 494 | S2103 | Naltrexone HCl | g3 | L1300-07 |
| 495 | S2104 | Levosulpiride | h3 | L1300-07 |
| 496 | S2105 | Pantoprazole | a4 | L1300-07 |
| 497 | S2108 | Flunixin Meglumin | b4 | L1300-07 |
| 498 | S2109 | Imidapril HCl | c4 | L1300-07 |
| 499 | S2111 | Lapatinib | d4 | L1300-07 |
| 500 | S2113 | Cisatracurium Besylate | e4 | L1300-07 |
| 501 | S2114 | Dronedarone HCl | f4 | L1300-07 |
| 502 | S2116 | Conivaptan HCl | g4 | L1300-07 |
| 503 | S2118 | Ibutilide Fumarate | h4 | L1300-07 |
| 504 | S2119 | Probucol | a5 | L1300-07 |
| 505 | S2120 | Arbidol HCl | b5 | L1300-07 |
| 506 | S2123 | Dextrose | c5 | L1300-07 |
| 507 | S2124 | Xylose | d5 | L1300-07 |
| 508 | S2125 | Mestranol | e5 | L1300-07 |
| 509 | S2126 | Naftopidil | f5 | L1300-07 |
| 510 | S2127 | S- (+)-Rolipram | g5 | L1300-07 |
| 511 | S2128 | Bazedoxifene HCl | h5 | L1300-07 |
| 512 | S2130 | Atropine sulfate monohydrate | a6 | L1300-07 |
| 513 | S2131 | Roflumilast | b6 | L1300-07 |
| 514 | S2151 | Sonidegib (Erismodegib, NVP-LDE225) | c6 | L1300-07 |
| 515 | S2154 | Dabigatran Etexilate | d6 | L1300-07 |
| 516 | S2159 | Tebipenem Pivoxil | e6 | L1300-07 |
| 517 | S2167 | Bazedoxifene Acetate | f6 | L1300-07 |
| 518 | S2169 | Rosuvastatin Calcium | g6 | L1300-07 |
| 519 | S2173 | Telotristat Etiprate (LX 1606 Hippurate) | h6 | L1300-07 |
| 520 | S2177 | Dichlorphenamide (Diclofenamide) | a7 | L1300-07 |
| 521 | S2180 | Ixazomib (MLN2238) | b7 | L1300-07 |
| 522 | S2181 | Ixazomib Citrate (MLN9708) | c7 | L1300-07 |
| 523 | S2199 | Aliskiren Hemifumarate | d7 | L1300-07 |
| 524 | S2208 | Formestane | e7 | L1300-07 |
| 525 | S2217 | Irinotecan HCl Trihydrate | f7 | L1300-07 |
| 526 | S2226 | Idelalisib (CAL-101, GS-1101) | h7 | L1300-07 |
| 527 | S2229 | Eltrombopag Olamine | a8 | L1300-07 |
| 528 | S2233 | Esomeprazole sodium | b8 | L1300-07 |
| 529 | S2240 | Fesoterodine Fumarate | c8 | L1300-07 |
| 530 | S2256 | 4-Methylumbelliferone (4-MU) | d8 | L1300-07 |
| 531 | S2258 | Esculin | e8 | L1300-07 |
| 532 | S2260 | Amygdalin | f8 | L1300-07 |
| 533 | S2261 | Andrographolide | g8 | L1300-07 |
| 534 | S2262 | Apigenin | h8 | L1300-07 |
| 535 | S2263 | Arbutin | a9 | L1300-07 |
| 536 | S2264 | Artemether | b9 | L1300-07 |
| 537 | S2265 | Artesunate | c9 | L1300-07 |
| 538 | S2268 | Baicalein | d9 | L1300-07 |
| 539 | S2269 | Baicalin | e9 | L1300-07 |
| 540 | S2270 | Bergenin | f9 | L1300-07 |
| 541 | S2271 | Berberine chloride | g9 | L1300-07 |
| 542 | S2277 | Caffeic Acid | h9 | L1300-07 |
| 543 | S2280 | Chlorogenic Acid | a10 | L1300-07 |
| 544 | S2282 | Cinchonidine | b10 | L1300-07 |
| 545 | S2283 | Cinchonine(LA40221) | c10 | L1300-07 |
| 546 | S2284 | Colchicine | d10 | L1300-07 |
| 547 | S2287 | Cytisine | e10 | L1300-07 |
| 548 | S2289 | Daidzin | f10 | L1300-07 |
| 549 | S2290 | Dihydroartemisinin (DHA) | g10 | L1300-07 |
| 550 | S2293 | DL-Carnitine HCl | h10 | L1300-07 |
| 551 | S2295 | Emodin | a11 | L1300-07 |
| 552 | S2296 | Enoxolone | b11 | L1300-07 |
| 553 | S2299 | Formononetin | c11 | L1300-07 |
| 554 | S2300 | Ferulic Acid | d11 | L1300-07 |
| 555 | S2302 | Glycyrrhizin (Glycyrrhizic Acid) | e11 | L1300-07 |
| 556 | S2304 | Gramine | f11 | L1300-07 |
| 557 | S2309 | Hesperidin | g11 | L1300-07 |
| 558 | S2310 | Honokiol | h11 | L1300-07 |
| 559 | S2311 | Hyodeoxycholic acid (HDCA) | a2 | L1300-08 |
| 560 | S2314 | Kaempferol | b2 | L1300-08 |
| 561 | S2316 | Kinetin | c2 | L1300-08 |
| 562 | S2317 | L-(+)-Rhamnose Monohydrate | d2 | L1300-08 |
| 563 | S2318 | Lappaconitine | e2 | L1300-08 |
| 564 | S2320 | Luteolin | f2 | L1300-08 |
| 565 | S2321 | Magnolol | g2 | L1300-08 |
| 566 | S2325 | Morin Hydrate | h2 | L1300-08 |
| 567 | S2328 | Nalidixic acid | a3 | L1300-08 |
| 568 | S2329 | Naringin | b3 | L1300-08 |
| 569 | S2331 | Neohesperidin Dihydrochalcone (Nhdc) | c3 | L1300-08 |
| 570 | S2332 | Neohesperidin | d3 | L1300-08 |
| 571 | S2334 | Oleanolic Acid | e3 | L1300-08 |
| 572 | S2336 | Orotic acid (6-Carboxyuracil) | f3 | L1300-08 |
| 573 | S2337 | Osthole | g3 | L1300-08 |
| 574 | S2338 | Oxymatrine | h3 | L1300-08 |
| 575 | S2339 | Paeonol | a4 | L1300-08 |
| 576 | S2341 | (-)-Parthenolide | b4 | L1300-08 |
| 577 | S2344 | Piperine | c4 | L1300-08 |
| 578 | S2346 | Puerarin | d4 | L1300-08 |
| 579 | S2347 | Quercetin Dihydrate | e4 | L1300-08 |
| 580 | S2348 | Rotenone (Barbasco) | f4 | L1300-08 |
| 581 | S2350 | Rutin | g4 | L1300-08 |
| 582 | S2351 | Salicin | h4 | L1300-08 |
| 583 | S2354 | Sclareol | a5 | L1300-08 |
| 584 | S2357 | Silibinin | b5 | L1300-08 |
| 585 | S2358 | Silymarin | c5 | L1300-08 |
| 586 | S2359 | Sinomenine | d5 | L1300-08 |
| 587 | S2362 | Synephrine | e5 | L1300-08 |
| 588 | S2364 | Tanshinone I | f5 | L1300-08 |
| 589 | S2365 | Tanshinone IIA | g5 | L1300-08 |
| 590 | S2366 | Taxifolin (Dihydroquercetin) | h5 | L1300-08 |
| 591 | S2369 | Troxerutin | a6 | L1300-08 |
| 592 | S2370 | Ursolic Acid | b6 | L1300-08 |
| 593 | S2373 | Yohimbine HCl | c6 | L1300-08 |
| 594 | S2374 | 5-hydroxytryptophan (5-HTP) | d6 | L1300-08 |
| 595 | S2375 | Aloin | e6 | L1300-08 |
| 596 | S2376 | Ammonium Glycyrrhizinate | f6 | L1300-08 |
| 597 | S2378 | Butylscopolamine Bromide | g6 | L1300-08 |
| 598 | S2380 | Diosmetin | h6 | L1300-08 |
| 599 | S2381 | D-Mannitol | a7 | L1300-08 |
| 600 | S2383 | Gastrodin | b7 | L1300-08 |
| 601 | S2384 | Hematoxylin | c7 | L1300-08 |
| 602 | S2386 | Indirubin | d7 | L1300-08 |
| 603 | S2388 | L-carnitine | e7 | L1300-08 |
| 604 | S2389 | Naringin Dihydrochalcone | f7 | L1300-08 |
| 605 | S2390 | Polydatin | g7 | L1300-08 |
| 606 | S2391 | Quercetin | h7 | L1300-08 |
| 607 | S2393 | Sorbitol | a8 | L1300-08 |
| 608 | S2396 | Salidroside | b8 | L1300-08 |
| 609 | S2397 | Palmatine chloride | c8 | L1300-08 |
| 610 | S2401 | Sodium Danshensu | d8 | L1300-08 |
| 611 | S2410 | Paeoniflorin | e8 | L1300-08 |
| 612 | S2411 | Geniposide | f8 | L1300-08 |
| 613 | S2422 | Ipriflavone (Osteofix) | g8 | L1300-08 |
| 614 | S2423 | (S)-10-Hydroxycamptothecin | h8 | L1300-08 |
| 615 | S2437 | Rotundine | a9 | L1300-08 |
| 616 | S2438 | Synephrine HCl | b9 | L1300-08 |
| 617 | S2439 | Guanosine | c9 | L1300-08 |
| 618 | S2442 | Inosine | d9 | L1300-08 |
| 619 | S2443 | Tolbutamide | e9 | L1300-08 |
| 620 | S2446 | Levosimendan | f9 | L1300-08 |
| 621 | S2450 | Equol | g9 | L1300-08 |
| 622 | S2451 | Amantadine HCl | h9 | L1300-08 |
| 623 | S2452 | Amfebutamone (Bupropion) HCl | a10 | L1300-08 |
| 624 | S2453 | Benserazide HCl | b10 | L1300-08 |
| 625 | S2454 | Bupivacaine HCl | c10 | L1300-08 |
| 626 | S2455 | Bethanechol chloride | d10 | L1300-08 |
| 627 | S2456 | Chlorpromazine HCl | e10 | L1300-08 |
| 628 | S2457 | Clindamycin HCl | f10 | L1300-08 |
| 629 | S2458 | Clonidine HCl | g10 | L1300-08 |
| 630 | S2459 | Clozapine | h10 | L1300-08 |
| 631 | S2460 | Pramipexole | a11 | L1300-08 |
| 632 | S2461 | Domperidone | b11 | L1300-08 |
| 633 | S2466 | Estriol | c11 | L1300-08 |
| 634 | S2467 | Famciclovir | d11 | L1300-08 |
| 635 | S2468 | Fenbendazole | e11 | L1300-08 |
| 636 | S2470 | Fluocinolone Acetonide | f11 | L1300-08 |
| 637 | S2471 | Gallamine Triethiodide | g11 | L1300-08 |
| 638 | S2473 | Hexestrol | h11 | L1300-08 |
| 639 | S2475 | Imatinib (STI571) | a2 | L1300-09 |
| 640 | S2479 | Lincomycin HCl | b2 | L1300-09 |
| 641 | S2480 | Loperamide HCl | c2 | L1300-09 |
| 642 | S2481 | Manidipine | d2 | L1300-09 |
| 643 | S2482 | Manidipine 2HCl | e2 | L1300-09 |
| 644 | S2484 | Milrinone | f2 | L1300-09 |
| 645 | S2485 | Mitoxantrone 2HCl | g2 | L1300-09 |
| 646 | S2486 | Moroxydine HCl | h2 | L1300-09 |
| 647 | S2487 | Mycophenolic acid | a3 | L1300-09 |
| 648 | S2489 | Nateglinide | b3 | L1300-09 |
| 649 | S2491 | Nitrendipine | c3 | L1300-09 |
| 650 | S2492 | Novobiocin Sodium | d3 | L1300-09 |
| 651 | S2493 | Olanzapine | e3 | L1300-09 |
| 652 | S2494 | Olopatadine HCl | f3 | L1300-09 |
| 653 | S2496 | Ozagrel | g3 | L1300-09 |
| 654 | S2497 | Pancuronium dibromide | h3 | L1300-09 |
| 655 | S2499 | Phenoxybenzamine HCl | a4 | L1300-09 |
| 656 | S2500 | Propafenone HCl | b4 | L1300-09 |
| 657 | S2501 | Pyrantel Pamoate | c4 | L1300-09 |
| 658 | S2502 | Quinine HCl Dihydrate | d4 | L1300-09 |
| 659 | S2503 | Racecadotril | e4 | L1300-09 |
| 660 | S2504 | Ribavirin | f4 | L1300-09 |
| 661 | S2505 | Rosiglitazone maleate | g4 | L1300-09 |
| 662 | S2506 | Roxithromycin | h4 | L1300-09 |
| 663 | S2508 | Scopolamine HBr | a5 | L1300-09 |
| 664 | S2509 | Sotalol HCl | b5 | L1300-09 |
| 665 | S2510 | Spectinomycin 2HCl | c5 | L1300-09 |
| 666 | S2511 | Sulfadoxine | d5 | L1300-09 |
| 667 | S2512 | Tenoxicam | e5 | L1300-09 |
| 668 | S2515 | Vardenafil HCl Trihydrate | f5 | L1300-09 |
| 669 | S2516 | Xylazine HCl | g5 | L1300-09 |
| 670 | S2517 | Maprotiline HCl | h5 | L1300-09 |
| 671 | S2519 | Naphazoline HCl | a6 | L1300-09 |
| 672 | S2521 | Epinephrine bitartrate | b6 | L1300-09 |
| 673 | S2522 | L-Adrenaline | c6 | L1300-09 |
| 674 | S2524 | Phenytoin Sodium | d6 | L1300-09 |
| 675 | S2525 | Phenytoin | e6 | L1300-09 |
| 676 | S2528 | Ciclopirox | f6 | L1300-09 |
| 677 | S2529 | Dopamine HCl | g6 | L1300-09 |
| 678 | S2533 | Ritodrine HCl | h6 | L1300-09 |
| 679 | S2534 | Isoconazole nitrate | a7 | L1300-09 |
| 680 | S2535 | Econazole nitrate | b7 | L1300-09 |
| 681 | S2536 | Miconazole | c7 | L1300-09 |
| 682 | S2537 | Secnidazole | d7 | L1300-09 |
| 683 | S2538 | Acetanilide | e7 | L1300-09 |
| 684 | S2541 | Clomipramine HCl | f7 | L1300-09 |
| 685 | S2542 | Phenformin HCl | g7 | L1300-09 |
| 686 | S2543 | Ceftiofur HCl | h7 | L1300-09 |
| 687 | S2547 | Tiotropium Bromide hydrate | a8 | L1300-09 |
| 688 | S2549 | Trospium chloride | b8 | L1300-09 |
| 689 | S2550 | Tolterodine tartrate | c8 | L1300-09 |
| 690 | S2551 | Sulbactam sodium | d8 | L1300-09 |
| 691 | S2552 | Azelastine HCl | e8 | L1300-09 |
| 692 | S2553 | 5-Aminolevulinic acid HCl | f8 | L1300-09 |
| 693 | S2554 | Daphnetin | g8 | L1300-09 |
| 694 | S2555 | Clarithromycin | h8 | L1300-09 |
| 695 | S2556 | Rosiglitazone | a9 | L1300-09 |
| 696 | S2557 | Terbinafine HCl | b9 | L1300-09 |
| 697 | S2559 | Cortisone acetate | c9 | L1300-09 |
| 698 | S2561 | Clomifene citrate | d9 | L1300-09 |
| 699 | S2564 | Cloxacillin Sodium | e9 | L1300-09 |
| 700 | S2565 | Amoxicillin Sodium | f9 | L1300-09 |
| 701 | S2566 | Isoprenaline HCl | g9 | L1300-09 |
| 702 | S2567 | Medroxyprogesterone acetate | h9 | L1300-09 |
| 703 | S2569 | Phenylephrine HCl | a10 | L1300-09 |
| 704 | S2570 | Prednisolone Acetate | b10 | L1300-09 |
| 705 | S2573 | Tetracaine HCl | c10 | L1300-09 |
| 706 | S2574 | Tetracycline HCl | d10 | L1300-09 |
| 707 | S2576 | Xylometazoline HCl | e10 | L1300-09 |
| 708 | S2577 | Phenacetin | f10 | L1300-09 |
| 709 | S2579 | Zidovudine | g10 | L1300-09 |
| 710 | S2581 | Quinapril HCl | h10 | L1300-09 |
| 711 | S2583 | Thiamphenicol | a11 | L1300-09 |
| 712 | S2584 | Clobetasol propionate | b11 | L1300-09 |
| 713 | S2585 | Brompheniramine hydrogen maleate | c11 | L1300-09 |
| 714 | S2586 | Dimethyl Fumarate | d11 | L1300-09 |
| 715 | S2589 | Miglitol | e11 | L1300-09 |
| 716 | S2590 | Pioglitazone | f11 | L1300-09 |
| 717 | S2593 | Tolvaptan | g11 | L1300-09 |
| 718 | S2594 | Pramiracetam | h11 | L1300-09 |
| 719 | S2596 | Clindamycin palmitate HCl | a2 | L1300-10 |
| 720 | S2599 | L-Thyroxine | b2 | L1300-10 |
| 721 | S2601 | Gliclazide | c2 | L1300-10 |
| 722 | S2602 | Acemetacin | d2 | L1300-10 |
| 723 | S2603 | Tioxolone | e2 | L1300-10 |
| 724 | S2604 | Dehydroepiandrosterone (DHEA) | f2 | L1300-10 |
| 725 | S2605 | Idebenone | g2 | L1300-10 |
| 726 | S2606 | Mifepristone | h2 | L1300-10 |
| 727 | S2607 | Buflomedil HCl | a3 | L1300-10 |
| 728 | S2608 | Fluocinonide | b3 | L1300-10 |
| 729 | S2609 | Inulin | c3 | L1300-10 |
| 730 | S2610 | Lonidamine | d3 | L1300-10 |
| 731 | S2613 | Clorsulon | e3 | L1300-10 |
| 732 | S2614 | Arecoline HBr | f3 | L1300-10 |
| 733 | S2615 | Noradrenaline bitartrate monohydrate | g3 | L1300-10 |
| 734 | S2625 | Fostamatinib (R788) | h3 | L1300-10 |
| 735 | S2664 | Clinofibrate | a4 | L1300-10 |
| 736 | S2665 | Ciprofibrate | b4 | L1300-10 |
| 737 | S2667 | Dolutegravir (GSK1349572) | c4 | L1300-10 |
| 738 | S2673 | Trametinib (GSK1120212) | d4 | L1300-10 |
| 739 | S2680 | Ibrutinib (PCI-32765) | e4 | L1300-10 |
| 740 | S2721 | Nilvadipine | f4 | L1300-10 |
| 741 | S2727 | Dacomitinib (PF299804, PF299) | g4 | L1300-10 |
| 742 | S2741 | Niraparib (MK-4827) | h4 | L1300-10 |
| 743 | S2760 | Canagliflozin | a5 | L1300-10 |
| 744 | S2762 | Alectinib (CH5424802) | b5 | L1300-10 |
| 745 | S2765 | MK-2048 | c5 | L1300-10 |
| 746 | S2787 | Laquinimod | d5 | L1300-10 |
| 747 | S2789 | Tofacitinib (CP-690550,Tasocitinib) | e5 | L1300-10 |
| 748 | S2790 | Istradefylline | f5 | L1300-10 |
| 749 | S2792 | Torcetrapib | g5 | L1300-10 |
| 750 | S2794 | Sofosbuvir (PSI-7977, GS-7977) | h5 | L1300-10 |
| 751 | S2807 | Dabrafenib (GSK2118436) | a6 | L1300-10 |
| 752 | S2809 | MPEP | b6 | L1300-10 |
| 753 | S2814 | Alpelisib (BYL719) | c6 | L1300-10 |
| 754 | S2830 | Clindamycin | d6 | L1300-10 |
| 755 | S2832 | Epiandrosterone | e6 | L1300-10 |
| 756 | S2840 | Apalutamide?(ARN-509) | f6 | L1300-10 |
| 757 | S2851 | Baricitinib (LY3009104, INCB028050) | g6 | L1300-10 |
| 758 | S2853 | Carfilzomib (PR-171) | h6 | L1300-10 |
| 759 | S2868 | Alogliptin（SYK-322）benzoate | a7 | L1300-10 |
| 760 | S2874 | Camostat Mesilate | b7 | L1300-10 |
| 761 | S2875 | Prucalopride | c7 | L1300-10 |
| 762 | S2884 | Acesulfame Potassium | d7 | L1300-10 |
| 763 | S2900 | Cobicistat (GS-9350) | e7 | L1300-10 |
| 764 | S2902 | S-Ruxolitinib (INCB018424) | f7 | L1300-10 |
| 765 | S2903 | Lumiracoxib | g7 | L1300-10 |
| 766 | S2907 | Pirfenidone | h7 | L1300-10 |
| 767 | S2922 | Icotinib | a8 | L1300-10 |
| 768 | S3000 | Carbazochrome sodium sulfonate (AC-17) | b8 | L1300-10 |
| 769 | S3001 | Clevudine | c8 | L1300-10 |
| 770 | S3002 | Rivaroxaban | d8 | L1300-10 |
| 771 | S3003 | Prostaglandin E2 (PGE2) | e8 | L1300-10 |
| 772 | S3005 | Paroxetine HCl | f8 | L1300-10 |
| 773 | S3008 | Zaltoprofen | g8 | L1300-10 |
| 774 | S3012 | Pazopanib | h8 | L1300-10 |
| 775 | S3015 | Amoxicillin | a9 | L1300-10 |
| 776 | S3017 | Aspirin | b9 | L1300-10 |
| 777 | S3018 | Niflumic acid | c9 | L1300-10 |
| 778 | S3019 | Ciclopirox ethanolamine | d9 | L1300-10 |
| 779 | S3021 | Rimonabant | e9 | L1300-10 |
| 780 | S3022 | Cabazitaxel | f9 | L1300-10 |
| 781 | S3023 | Bufexamac | g9 | L1300-10 |
| 782 | S3024 | Lamotrigine | h9 | L1300-10 |
| 783 | S3027 | Fenoprofen calcium hydrate | a10 | L1300-10 |
| 784 | S3031 | Linagliptin | b10 | L1300-10 |
| 785 | S3033 | Vildagliptin (LAF-237) | c10 | L1300-10 |
| 786 | S3035 | Daunorubicin HCl | d10 | L1300-10 |
| 787 | S3036 | Pravastatin sodium | e10 | L1300-10 |
| 788 | S3037 | Bepotastine Besilate | f10 | L1300-10 |
| 789 | S3038 | Fosaprepitant dimeglumine salt | g10 | L1300-10 |
| 790 | S3043 | Rofecoxib | h10 | L1300-10 |
| 791 | S3045 | Cinepazide maleate | a11 | L1300-10 |
| 792 | S3047 | Otilonium Bromide | b11 | L1300-10 |
| 793 | S3051 | Bosentan Hydrate | c11 | L1300-10 |
| 794 | S3052 | Rupatadine Fumarate | d11 | L1300-10 |
| 795 | S3053 | Azelnidipine | e11 | L1300-10 |
| 796 | S3054 | Alverine Citrate | f11 | L1300-10 |
| 797 | S3057 | Azilsartan Medoxomil | g11 | L1300-10 |
| 798 | S3060 | Medetomidine HCl | h11 | L1300-10 |
| 799 | S1214 | Bleomycin sulfate | a2 | L1300-11 |
| 800 | S1218 | Clofarabine | b2 | L1300-11 |
| 801 | S1221 | Dacarbazine | c2 | L1300-11 |
| 802 | S1222 | Dexrazoxane HCl (ICRF-187, ADR-529) | d2 | L1300-11 |
| 803 | S3061 | Epinephrine HCl | e2 | L1300-11 |
| 804 | S3062 | Diclofenac Potassium | f2 | L1300-11 |
| 805 | S3063 | Diclofenac Diethylamine | g2 | L1300-11 |
| 806 | S3066 | Naloxone HCl | h2 | L1300-11 |
| 807 | S3067 | Chlorhexidine?2HCl | a3 | L1300-11 |
| 808 | S3070 | Piracetam | b3 | L1300-11 |
| 809 | S3071 | Vanillin | c3 | L1300-11 |
| 810 | S3074 | Chlorthalidone | d3 | L1300-11 |
| 811 | S3075 | Dexmedetomidine | e3 | L1300-11 |
| 812 | S3077 | Tazobactam | g3 | L1300-11 |
| 813 | S3078 | Beclomethasone dipropionate | h3 | L1300-11 |
| 814 | S3079 | Atovaquone | a4 | L1300-11 |
| 815 | S3080 | Etravirine (TMC125) | b4 | L1300-11 |
| 816 | S3081 | Ulipristal | c4 | L1300-11 |
| 817 | S3083 | Indacaterol Maleate | d4 | L1300-11 |
| 818 | S3100 | 2-Thiouracil | e4 | L1300-11 |
| 819 | S3104 | Moguisteine | f4 | L1300-11 |
| 820 | S3105 | Nadifloxacin | g4 | L1300-11 |
| 821 | S3106 | Pidotimod | h4 | L1300-11 |
| 822 | S3113 | Pyridoxine HCl | a5 | L1300-11 |
| 823 | S3114 | Vitamin C | b5 | L1300-11 |
| 824 | S3116 | Sulfathiazole | c5 | L1300-11 |
| 825 | S3117 | Oxybutynin chloride | d5 | L1300-11 |
| 826 | S3120 | Doxepin HCl | e5 | L1300-11 |
| 827 | S3121 | Ornidazole | f5 | L1300-11 |
| 828 | S3124 | Dexamethasone Acetate | g5 | L1300-11 |
| 829 | S3129 | Trimethoprim | h5 | L1300-11 |
| 830 | S3130 | Biotin (Vitamin B7) | a6 | L1300-11 |
| 831 | S3132 | Sulfamerazine | b6 | L1300-11 |
| 832 | S3133 | Sulfamethazine | c6 | L1300-11 |
| 833 | S3137 | Sodium salicylate | d6 | L1300-11 |
| 834 | S3138 | Methylthiouracil | e6 | L1300-11 |
| 835 | S3140 | Milnacipran HCl | g6 | L1300-11 |
| 836 | S3144 | Darifenacin HBr | h6 | L1300-11 |
| 837 | S3147 | Entacapone | a7 | L1300-11 |
| 838 | S3149 | Estradiol valerate | b7 | L1300-11 |
| 839 | S3150 | Articaine HCl | c7 | L1300-11 |
| 840 | S3151 | Gliquidone | d7 | L1300-11 |
| 841 | S3154 | Butenafine HCl | e7 | L1300-11 |
| 842 | S3155 | Mepivacaine HCl | f7 | L1300-11 |
| 843 | S3160 | Ethynodiol diacetate | g7 | L1300-11 |
| 844 | S3161 | Sertaconazole nitrate | h7 | L1300-11 |
| 845 | S3162 | Tylosin tartrate | a8 | L1300-11 |
| 846 | S3163 | Benztropine mesylate | b8 | L1300-11 |
| 847 | S3167 | Altrenogest | c8 | L1300-11 |
| 848 | S3170 | Ampicillin sodium | d8 | L1300-11 |
| 849 | S3172 | Anagrelide HCl | e8 | L1300-11 |
| 850 | S3173 | Antipyrine | f8 | L1300-11 |
| 851 | S3175 | Atomoxetine HCl | g8 | L1300-11 |
| 852 | S3176 | Betahistine 2HCl | h8 | L1300-11 |
| 853 | S3178 | Brinzolamide | a9 | L1300-11 |
| 854 | S3179 | Carbenicillin disodium | b9 | L1300-11 |
| 855 | S3180 | Eletriptan HBr | c9 | L1300-11 |
| 856 | S3181 | Flumequine | d9 | L1300-11 |
| 857 | S3183 | Amitriptyline HCl | e9 | L1300-11 |
| 858 | S3185 | Adrenalone HCl | f9 | L1300-11 |
| 859 | S3186 | Azatadine dimaleate | g9 | L1300-11 |
| 860 | S3188 | (+,-)-Octopamine HCl | h9 | L1300-11 |
| 861 | S3189 | Ropinirole HCl | a10 | L1300-11 |
| 862 | S3193 | Ticarcillin sodium | b10 | L1300-11 |
| 863 | S3195 | Azlocillin sodium salt | c10 | L1300-11 |
| 864 | S3199 | Reboxetine mesylate | d10 | L1300-11 |
| 865 | S3200 | Triflusal | e10 | L1300-11 |
| 866 | S3201 | Trifluoperazine 2HCl | f10 | L1300-11 |
| 867 | S3202 | Catharanthine | g10 | L1300-11 |
| 868 | S3204 | Meptazinol HCl | h10 | L1300-11 |
| 869 | S3207 | Iopromide | a11 | L1300-11 |
| 870 | S3208 | Fexofenadine HCl | b11 | L1300-11 |
| 871 | S3212 | Moclobemide (Ro 111163) | c11 | L1300-11 |
| 872 | S3604 | Triptolide (PG490) | d11 | L1300-11 |
| 873 | S3605 | Borneol | e11 | L1300-11 |
| 874 | S3606 | Fangchinoline | f11 | L1300-11 |
| 875 | S3609 | Berbamine (dihydrochloride) | g11 | L1300-11 |
| 876 | S3611 | (+)-Fangchinoline | h11 | L1300-11 |
| 877 | S3612 | Rosmarinic acid | a2 | L1300-12 |
| 878 | S3615 | Dehydrocostus Lactone | b2 | L1300-12 |
| 879 | S3616 | Asiaticoside | c2 | L1300-12 |
| 880 | S3618 | Acetylspiramycin (ASPM) | d2 | L1300-12 |
| 881 | S3621 | Pazufloxacin mesylate | e2 | L1300-12 |
| 882 | S3622 | Diammonium Glycyrrhizinate | f2 | L1300-12 |
| 883 | S3623 | Ceftibuten dihydrate | g2 | L1300-12 |
| 884 | S3625 | Tyramine | h2 | L1300-12 |
| 885 | S3635 | Medroxyprogesterone | a3 | L1300-12 |
| 886 | S3637 | Cefpirome sulfate | c3 | L1300-12 |
| 887 | S3638 | Cefamandole nafate | d3 | L1300-12 |
| 888 | S3639 | Tacrine hydrochloride hydrate | e3 | L1300-12 |
| 889 | S3640 | Methoxyphenamine Hydrochloride | f3 | L1300-12 |
| 890 | S3641 | Osalmid | g3 | L1300-12 |
| 891 | S3643 | Amitraz | h3 | L1300-12 |
| 892 | S3644 | Sulfamonomethoxine | a4 | L1300-12 |
| 893 | S3645 | Kitasamycin | b4 | L1300-12 |
| 894 | S3646 | Thimerosal | c4 | L1300-12 |
| 895 | S3647 | Mafenide Acetate | d4 | L1300-12 |
| 896 | S3648 | Amlexanox | e4 | L1300-12 |
| 897 | S3654 | Tauroursodeoxycholic Acid (TUDCA) | f4 | L1300-12 |
| 898 | S3655 | Cefepime Dihydrochloride Monohydrate | g4 | L1300-12 |
| 899 | S3656 | Piribedil | h4 | L1300-12 |
| 900 | S3657 | Promestriene | a5 | L1300-12 |
| 901 | S3659 | Fludrocortisone acetate | b5 | L1300-12 |
| 902 | S3663 | Afloqualone | c5 | L1300-12 |
| 903 | S3664 | Flupenthixol dihydrochloride | d5 | L1300-12 |
| 904 | S3666 | Ilaprazole | e5 | L1300-12 |
| 905 | S3668 | Thymopentin | f5 | L1300-12 |
| 906 | S3669 | Carmustine | g5 | L1300-12 |
| 907 | S3670 | Cefsulodin sodium | h5 | L1300-12 |
| 908 | S3671 | Quinestrol | a6 | L1300-12 |
| 909 | S3672 | Cefonicid sodium | b6 | L1300-12 |
| 910 | S3673 | Sulfaphenazole | c6 | L1300-12 |
| 911 | S3674 | Levamlodipine | d6 | L1300-12 |
| 912 | S3675 | Umbelliferone | e6 | L1300-12 |
| 913 | S3677 | Cinnamic acid | f6 | L1300-12 |
| 914 | S3681 | Vitamin E Acetate | g6 | L1300-12 |
| 915 | S3684 | Methacholine chloride | h6 | L1300-12 |
| 916 | S3685 | 4-Biphenylacetic acid | a7 | L1300-12 |
| 917 | S3689 | Ethidium bromide | b7 | L1300-12 |
| 918 | S3690 | Pargyline hydrochloride | c7 | L1300-12 |
| 919 | S3692 | N-Ethylmaleimide (NEM) | d7 | L1300-12 |
| 920 | S3694 | Glucosamine hydrochloride | e7 | L1300-12 |
| 921 | S3697 | Mafenide hydrochloride | f7 | L1300-12 |
| 922 | S3698 | Nortriptyline hydrochloride | g7 | L1300-12 |
| 923 | S3701 | Benactyzine hydrochloride | h7 | L1300-12 |
| 924 | S3703 | Phenethyl alcohol | a8 | L1300-12 |
| 925 | S3704 | 4-Methylbenzylidene camphor | b8 | L1300-12 |
| 926 | S3705 | Chlorobutanol | c8 | L1300-12 |
| 927 | S3706 | Sarpogrelate hydrochloride | d8 | L1300-12 |
| 928 | S3707 | Ethopabate | e8 | L1300-12 |
| 929 | S3708 | Sulfachloropyridazine | f8 | L1300-12 |
| 930 | S3711 | Carbasalate Calcium | g8 | L1300-12 |
| 931 | S3713 | Moxidectin | h8 | L1300-12 |
| 932 | S3714 | Lifitegrast | a9 | L1300-12 |
| 933 | S3716 | Flibanserin | b9 | L1300-12 |
| 934 | S3717 | Testosterone Enanthate | c9 | L1300-12 |
| 935 | S3718 | Leuprolide Acetate | d9 | L1300-12 |
| 936 | S3719 | Topiroxostat | e9 | L1300-12 |
| 937 | S3722 | Isavuconazole | f9 | L1300-12 |
| 938 | S3723 | Ramosetron Hydrochloride | g9 | L1300-12 |
| 939 | S3724 | Velpatasvir | h9 | L1300-12 |
| 940 | S3726 | Selexipag | a10 | L1300-12 |
| 941 | S3727 | Vilanterol Trifenate | b10 | L1300-12 |
| 942 | S3728 | Grazoprevir | c10 | L1300-12 |
| 943 | S3729 | Iron sucrose | d10 | L1300-12 |
| 944 | S3730 | Metaxalone | e10 | L1300-12 |
| 945 | S3731 | Tipiracil hydrochloride | f10 | L1300-12 |
| 946 | S3732 | Avibactam sodium | g10 | L1300-12 |
| 947 | S3733 | Boceprevir | h10 | L1300-12 |
| 948 | S3735 | Umeclidinium bromide | a11 | L1300-12 |
| 949 | S3738 | Travoprost | b11 | L1300-12 |
| 950 | S3739 | Calcipotriene | c11 | L1300-12 |
| 951 | S3741 | Benznidazole | d11 | L1300-12 |
| 952 | S3742 | Cholic acid | e11 | L1300-12 |
| 953 | S3745 | Balsalazide disodium | g11 | L1300-12 |
| 954 | S3746 | Lumefantrine | h11 | L1300-12 |
| 955 | S3747 | Levothyroxine sodium | a2 | L1300-13 |
| 956 | S3750 | Sodium benzoate | b2 | L1300-13 |
| 957 | S3751 | Quinidine sulfate | c2 | L1300-13 |
| 958 | S3754 | 4-Hydroxybenzoic acid | d2 | L1300-13 |
| 959 | S3755 | Betaine | e2 | L1300-13 |
| 960 | S3756 | Methyl salicylate | f2 | L1300-13 |
| 961 | S3758 | Sinomenine hydrochloride | g2 | L1300-13 |
| 962 | S3761 | Eucalyptol | h2 | L1300-13 |
| 963 | S3763 | Cinnamaldehyde | a3 | L1300-13 |
| 964 | S3766 | Tanshinone IIA sulfonate (sodium) | b3 | L1300-13 |
| 965 | S3769 | Palmatine | c3 | L1300-13 |
| 966 | S3772 | 5-Hydroxymethylfurfural | d3 | L1300-13 |
| 967 | S3773 | Tyrosol | e3 | L1300-13 |
| 968 | S3775 | Ligustrazine hydrochloride | f3 | L1300-13 |
| 969 | S3779 | cis-Anethole | g3 | L1300-13 |
| 970 | S3781 | Ginkgolide C | h3 | L1300-13 |
| 971 | S3783 | Echinacoside | a4 | L1300-13 |
| 972 | S3785 | Notoginsenoside R1 | b4 | L1300-13 |
| 973 | S3788 | Carvacrol | c4 | L1300-13 |
| 974 | S3791 | Succinic acid | d4 | L1300-13 |
| 975 | S3794 | Palmitic acid | e4 | L1300-13 |
| 976 | S3802 | Trigonelline Hydrochloride | f4 | L1300-13 |
| 977 | S3805 | Stevioside | g4 | L1300-13 |
| 978 | S3807 | Dehydroandrographolide | h4 | L1300-13 |
| 979 | S3809 | Imperatorin | a5 | L1300-13 |
| 980 | S3810 | Scutellarin | b5 | L1300-13 |
| 981 | S3811 | Ginsenoside Re | c5 | L1300-13 |
| 982 | S3817 | Harmine hydrochloride | d5 | L1300-13 |
| 983 | S3824 | Quercitrin | e5 | L1300-13 |
| 984 | S3835 | Loganin | f5 | L1300-13 |
| 985 | S3842 | Isoquercitrin | g5 | L1300-13 |
| 986 | S3843 | Madecassoside | h5 | L1300-13 |
| 987 | S3847 | Panaxatriol | a6 | L1300-13 |
| 988 | S3849 | D-Galactose | b6 | L1300-13 |
| 989 | S3850 | Glucosamine sulfate | c6 | L1300-13 |
| 990 | S3851 | Camphor | d6 | L1300-13 |
| 991 | S3854 | Tetrahydropalmatine hydrochloride | e6 | L1300-13 |
| 992 | S3856 | Allantoin | f6 | L1300-13 |
| 993 | S3858 | Lawsone | g6 | L1300-13 |
| 994 | S3866 | Galanthamine | h6 | L1300-13 |
| 995 | S3868 | Harmine | a7 | L1300-13 |
| 996 | S3872 | Guaiacol | b7 | L1300-13 |
| 997 | S3876 | Indigo | c7 | L1300-13 |
| 998 | S3881 | Scopoletin | d7 | L1300-13 |
| 999 | S3883 | Protopine | e7 | L1300-13 |
| 1000 | S3885 | Pyrogallol | f7 | L1300-13 |
| 1001 | S3887 | L-Rhamnose monohydrate | g7 | L1300-13 |
| 1002 | S3889 | Arteether | h7 | L1300-13 |
| 1003 | S3892 | Isopsoralen | a8 | L1300-13 |
| 1004 | S3893 | Bornyl acetate | b8 | L1300-13 |
| 1005 | S3895 | Sophoridine | c8 | L1300-13 |
| 1006 | S3898 | Hydroxy Camptothecine | d8 | L1300-13 |
| 1007 | S3899 | Hederagenin | e8 | L1300-13 |
| 1008 | S3901 | Astragaloside IV | f8 | L1300-13 |
| 1009 | S3909 | Catalpol | g8 | L1300-13 |
| 1010 | S3914 | α-Hederin | h8 | L1300-13 |
| 1011 | S3919 | Hederacoside C | a9 | L1300-13 |
| 1012 | S3921 | Lathyrol | b9 | L1300-13 |
| 1013 | S3923 | Ginsenoside Rg1 | c9 | L1300-13 |
| 1014 | S3924 | Ginsenoside Rb1 | d9 | L1300-13 |
| 1015 | S3925 | (-)-Epicatechin gallate | e9 | L1300-13 |
| 1016 | S3926 | Forsythin | f9 | L1300-13 |
| 1017 | S3927 | Swertiamarin | g9 | L1300-13 |
| 1018 | S3930 | Liquiritin | h9 | L1300-13 |
| 1019 | S3935 | Nonivamide | a10 | L1300-13 |
| 1020 | S3944 | Valproic acid | b10 | L1300-13 |
| 1021 | S3945 | L-Cycloserine | c10 | L1300-13 |
| 1022 | S3946 | Mesterolone | d10 | L1300-13 |
| 1023 | S3950 | Maltitol | f10 | L1300-13 |
| 1024 | S3951 | Tannic acid | g10 | L1300-13 |
| 1025 | S3957 | Gamma-Oryzanol | h10 | L1300-13 |
| 1026 | S3959 | (+)-Borneol | a11 | L1300-13 |
| 1027 | S3965 | Vanillyl Butyl Ether | b11 | L1300-13 |
| 1028 | S3966 | Nifuratel | c11 | L1300-13 |
| 1029 | S3967 | Flavone | d11 | L1300-13 |
| 1030 | S3968 | Histamine | e11 | L1300-13 |
| 1031 | S3969 | Veratric acid | f11 | L1300-13 |
| 1032 | S3970 | Vindoline | g11 | L1300-13 |
| 1033 | S3971 | Fusidine | h11 | L1300-13 |
| 1034 | S3972 | Lobeline hydrochloride | a2 | L1300-14 |
| 1035 | S3974 | (+)-Catechin hydrate | b2 | L1300-14 |
| 1036 | S3975 | Protocatechuic acid | c2 | L1300-14 |
| 1037 | S3977 | (-)-Borneol | d2 | L1300-14 |
| 1038 | S3979 | Zinc Undecylenate | e2 | L1300-14 |
| 1039 | S3980 | Pyridoxine | f2 | L1300-14 |
| 1040 | S3982 | Batyl alcohol | g2 | L1300-14 |
| 1041 | S3983 | Caryophyllene oxide | h2 | L1300-14 |
| 1042 | S3984 | Nordihydroguaiaretic acid (NDGA) | a3 | L1300-14 |
| 1043 | S3985 | Methyl 4-hydroxybenzoate | b3 | L1300-14 |
| 1044 | S3986 | L(+)-Arabinose | c3 | L1300-14 |
| 1045 | S3987 | L-Tryptophan | d3 | L1300-14 |
| 1046 | S3992 | D-(+)-Trehalose dihydrate | e3 | L1300-14 |
| 1047 | S3995 | Guaiazulene | f3 | L1300-14 |
| 1048 | S3996 | Thioctic acid | g3 | L1300-14 |
| 1049 | S3997 | Oxaceprol | h3 | L1300-14 |
| 1050 | S3998 | (+)-α-Lipoic acid | a4 | L1300-14 |
| 1051 | S4000 | Pergolide Mesylate | b4 | L1300-14 |
| 1052 | S4001 | Cabozantinib malate (XL184) | c4 | L1300-14 |
| 1053 | S4002 | Sitagliptin phosphate monohydrate | d4 | L1300-14 |
| 1054 | S4003 | Lithocholic acid | e4 | L1300-14 |
| 1055 | S4004 | Ethambutol 2HCl | f4 | L1300-14 |
| 1056 | S4007 | Pentamidine isethionate | g4 | L1300-14 |
| 1057 | S4009 | Mirabegron | h4 | L1300-14 |
| 1058 | S4010 | Acebutolol HCl | a5 | L1300-14 |
| 1059 | S4011 | Ampiroxicam | b5 | L1300-14 |
| 1060 | S4012 | Desloratadine | c5 | L1300-14 |
| 1061 | S4014 | Hyoscyamine | d5 | L1300-14 |
| 1062 | S4016 | Ouabain | e5 | L1300-14 |
| 1063 | S4017 | Allylthiourea | f5 | L1300-14 |
| 1064 | S4018 | Sennoside B | g5 | L1300-14 |
| 1065 | S4019 | Avanafil | h5 | L1300-14 |
| 1066 | S4020 | Sodium Picosulfate | a6 | L1300-14 |
| 1067 | S4021 | Tolcapone | b6 | L1300-14 |
| 1068 | S4022 | Probenecid | c6 | L1300-14 |
| 1069 | S4023 | Procaine HCl | d6 | L1300-14 |
| 1070 | S4024 | Homatropine Methylbromide | e6 | L1300-14 |
| 1071 | S4025 | Homatropine Bromide | f6 | L1300-14 |
| 1072 | S4026 | Hydroxyzine 2HCl | g6 | L1300-14 |
| 1073 | S4031 | Aclidinium Bromide | h6 | L1300-14 |
| 1074 | S4034 | Diphemanil Methylsulfate | b7 | L1300-14 |
| 1075 | S4035 | Vitamin D2 | c7 | L1300-14 |
| 1076 | S4037 | Doxapram HCl | d7 | L1300-14 |
| 1077 | S4038 | Dibucaine HCl | e7 | L1300-14 |
| 1078 | S4039 | Methazolamide | f7 | L1300-14 |
| 1079 | S4040 | Norethindrone | g7 | L1300-14 |
| 1080 | S4041 | Olsalazine Sodium | h7 | L1300-14 |
| 1081 | S4042 | Nafcillin Sodium | a8 | L1300-14 |
| 1082 | S4043 | Tetrahydrozoline HCl | b8 | L1300-14 |
| 1083 | S4044 | Toltrazuril | c8 | L1300-14 |
| 1084 | S4045 | Pheniramine Maleate | d8 | L1300-14 |
| 1085 | S4046 | Estradiol Cypionate | e8 | L1300-14 |
| 1086 | S4047 | Bisacodyl | f8 | L1300-14 |
| 1087 | S4048 | Carbimazole | g8 | L1300-14 |
| 1088 | S4049 | Valdecoxib | h8 | L1300-14 |
| 1089 | S4050 | Valganciclovir HCl | a9 | L1300-14 |
| 1090 | S4051 | Nabumetone | b9 | L1300-14 |
| 1091 | S4053 | Sertraline HCl | c9 | L1300-14 |
| 1092 | S4054 | Spironolactone | d9 | L1300-14 |
| 1093 | S4056 | Retapamulin | e9 | L1300-14 |
| 1094 | S4057 | Methyclothiazide | f9 | L1300-14 |
| 1095 | S4058 | Ropivacaine HCl | g9 | L1300-14 |
| 1096 | S4059 | Sodium Nitroprusside Dihydrate | h9 | L1300-14 |
| 1097 | S4060 | Erythromycin Ethylsuccinate | a10 | L1300-14 |
| 1098 | S4061 | Levobupivacaine HCl | b10 | L1300-14 |
| 1099 | S4062 | Ronidazole | c10 | L1300-14 |
| 1100 | S4063 | cholecalciferol (Vitamin D3) | d10 | L1300-14 |
| 1101 | S4064 | Escitalopram Oxalate | e10 | L1300-14 |
| 1102 | S4065 | Guanabenz Acetate | f10 | L1300-14 |
| 1103 | S4068 | Tinidazole | g10 | L1300-14 |
| 1104 | S4070 | Guanidine HCl | h10 | L1300-14 |
| 1105 | S4071 | Griseofulvin | a11 | L1300-14 |
| 1106 | S4072 | Decamethonium Bromide | b11 | L1300-14 |
| 1107 | S4074 | Sodium Nitrite | c11 | L1300-14 |
| 1108 | S4075 | Zinc Pyrithione | d11 | L1300-14 |
| 1109 | S4076 | Propranolol HCl | e11 | L1300-14 |
| 1110 | S4077 | Mequinol | f11 | L1300-14 |
| 1111 | S4078 | Mefenamic Acid | g11 | L1300-14 |
| 1112 | S4079 | Ticagrelor | h11 | L1300-14 |
| 1113 | S4080 | Triamterene | a2 | L1300-15 |
| 1114 | S4081 | Sulfacetamide Sodium | b2 | L1300-15 |
| 1115 | S4082 | Spiramycin | c2 | L1300-15 |
| 1116 | S4083 | Vitamin A Acetate | d2 | L1300-15 |
| 1117 | S4084 | Lomerizine 2HCl | e2 | L1300-15 |
| 1118 | S4085 | Levobetaxolol HCl | f2 | L1300-15 |
| 1119 | S4086 | Loxapine Succinate | g2 | L1300-15 |
| 1120 | S4088 | Flumethasone | h2 | L1300-15 |
| 1121 | S4089 | Halobetasol Propionate | a3 | L1300-15 |
| 1122 | S4090 | Fenspiride HCl | b3 | L1300-15 |
| 1123 | S4091 | Ifenprodil Tartrate | c3 | L1300-15 |
| 1124 | S4092 | Pramoxine HCl | d3 | L1300-15 |
| 1125 | S4095 | Difluprednate | e3 | L1300-15 |
| 1126 | S4096 | Droperidol | f3 | L1300-15 |
| 1127 | S4098 | Halcinonide | g3 | L1300-15 |
| 1128 | S4099 | Dexlansoprazole | h3 | L1300-15 |
| 1129 | S4100 | Esmolol HCl | a4 | L1300-15 |
| 1130 | S4101 | Voglibose | b4 | L1300-15 |
| 1131 | S4102 | Eprosartan Mesylate | c4 | L1300-15 |
| 1132 | S4104 | Diminazene Aceturate | d4 | L1300-15 |
| 1133 | S4105 | Closantel Sodium | e4 | L1300-15 |
| 1134 | S4106 | Closantel | f4 | L1300-15 |
| 1135 | S4107 | Clofazimine | g4 | L1300-15 |
| 1136 | S4110 | Estradiol Benzoate | h4 | L1300-15 |
| 1137 | S4111 | Dicloxacillin Sodium | a5 | L1300-15 |
| 1138 | S4112 | Desvenlafaxine Succinate | b5 | L1300-15 |
| 1139 | S4113 | Desvenlafaxine | c5 | L1300-15 |
| 1140 | S4114 | Triclabendazole | d5 | L1300-15 |
| 1141 | S4118 | Histamine 2HCl | e5 | L1300-15 |
| 1142 | S4119 | Pefloxacin Mesylate Dihydrate | f5 | L1300-15 |
| 1143 | S4120 | Sulconazole Nitrate | g5 | L1300-15 |
| 1144 | S4122 | Tilmicosin | h5 | L1300-15 |
| 1145 | S4123 | Timolol Maleate | a6 | L1300-15 |
| 1146 | S4124 | Tolazoline HCl | b6 | L1300-15 |
| 1147 | S4125 | Sodium Phenylbutyrate | c6 | L1300-15 |
| 1148 | S4128 | Troxipide | d6 | L1300-15 |
| 1149 | S4131 | Levodropropizine | e6 | L1300-15 |
| 1150 | S4135 | Clorprenaline HCl | f6 | L1300-15 |
| 1151 | S4136 | Carprofen | g6 | L1300-15 |
| 1152 | S4138 | Dropropizine | h6 | L1300-15 |
| 1153 | S4139 | Cyclizine 2HCl | a7 | L1300-15 |
| 1154 | S4141 | Dinitolmide | b7 | L1300-15 |
| 1155 | S4143 | Pentoxyverine Citrate | c7 | L1300-15 |
| 1156 | S4147 | Azithromycin Dihydrate | d7 | L1300-15 |
| 1157 | S4148 | Ampicillin Trihydrate | e7 | L1300-15 |
| 1158 | S4149 | Amfenac Sodium Monohydrate | f7 | L1300-15 |
| 1159 | S4151 | Penfluridol | g7 | L1300-15 |
| 1160 | S4152 | Ethamsylate | h7 | L1300-15 |
| 1161 | S4155 | Chlorzoxazone | a8 | L1300-15 |
| 1162 | S4159 | Bezafibrate | b8 | L1300-15 |
| 1163 | S4160 | Penicillin G Sodium | c8 | L1300-15 |
| 1164 | S4161 | Benzoic Acid | d8 | L1300-15 |
| 1165 | S4162 | Benzethonium Chloride | e8 | L1300-15 |
| 1166 | S4163 | Doxycycline Hyclate | f8 | L1300-15 |
| 1167 | S4164 | Doxofylline | g8 | L1300-15 |
| 1168 | S4165 | Benzydamine HCl | h8 | L1300-15 |
| 1169 | S4166 | Chlorpropamide | a9 | L1300-15 |
| 1170 | S4167 | Cyromazine | b9 | L1300-15 |
| 1171 | S4169 | Teriflunomide | c9 | L1300-15 |
| 1172 | S4170 | Coumarin | d9 | L1300-15 |
| 1173 | S4171 | Choline Chloride | e9 | L1300-15 |
| 1174 | S4172 | Cetylpyridinium Chloride | f9 | L1300-15 |
| 1175 | S4173 | 1-Hexadecanol | g9 | L1300-15 |
| 1176 | S4175 | Sulfaguanidine | h9 | L1300-15 |
| 1177 | S4176 | Trometamol | a10 | L1300-15 |
| 1178 | S4177 | Uracil | b10 | L1300-15 |
| 1179 | S4178 | Climbazole | c10 | L1300-15 |
| 1180 | S4179 | Mezlocillin Sodium | d10 | L1300-15 |
| 1181 | S4181 | Nicardipine HCl | e10 | L1300-15 |
| 1182 | S4182 | Nifuroxazide | f10 | L1300-15 |
| 1183 | S4184 | Penciclovir | g10 | L1300-15 |
| 1184 | S4185 | Tiratricol | h10 | L1300-15 |
| 1185 | S4186 | Domiphen Bromide | a11 | L1300-15 |
| 1186 | S4187 | Salicylanilide | b11 | L1300-15 |
| 1187 | S4188 | Sasapyrine | c11 | L1300-15 |
| 1188 | S4189 | Cyclandelate | d11 | L1300-15 |
| 1189 | S4191 | Betamipron | e11 | L1300-15 |
| 1190 | S4192 | Chlorquinaldol | f11 | L1300-15 |
| 1191 | S4195 | Broxyquinoline | g11 | L1300-15 |
| 1192 | S4196 | Ethacridine lactate monohydrate | h11 | L1300-15 |
| 1193 | S4197 | Bemegride | a2 | L1300-16 |
| 1194 | S4200 | Tolperisone HCl | b2 | L1300-16 |
| 1195 | S4201 | Florfenicol | c2 | L1300-16 |
| 1196 | S4202 | Verapamil HCl | d2 | L1300-16 |
| 1197 | S4203 | Furaltadone HCl | e2 | L1300-16 |
| 1198 | S4204 | Isosorbide | f2 | L1300-16 |
| 1199 | S4206 | Cysteamine HCl | g2 | L1300-16 |
| 1200 | S4207 | Clofibric Acid | h2 | L1300-16 |
| 1201 | S4208 | Chromocarb | a3 | L1300-16 |
| 1202 | S4209 | Chlorocresol | b3 | L1300-16 |
| 1203 | S4210 | Benzocaine | c3 | L1300-16 |
| 1204 | S4211 | Montelukast Sodium | d3 | L1300-16 |
| 1205 | S4213 | Dirithromycin | e3 | L1300-16 |
| 1206 | S4214 | Sucralose | f3 | L1300-16 |
| 1207 | S4216 | Valnemulin HCl | g3 | L1300-16 |
| 1208 | S4217 | Liothyronine Sodium | h3 | L1300-16 |
| 1209 | S4219 | Azaperone | a4 | L1300-16 |
| 1210 | S4221 | Benzbromarone | b4 | L1300-16 |
| 1211 | S4222 | Piperacillin Sodium | c4 | L1300-16 |
| 1212 | S4223 | Mevastatin | d4 | L1300-16 |
| 1213 | S4224 | Erythritol | e4 | L1300-16 |
| 1214 | S4225 | Mexiletine HCl | f4 | L1300-16 |
| 1215 | S4227 | Fidaxomicin | g4 | L1300-16 |
| 1216 | S4228 | Fluorometholone Acetate | h4 | L1300-16 |
| 1217 | S4229 | Oxybuprocaine HCl | a5 | L1300-16 |
| 1218 | S4230 | Oxaprozin | b5 | L1300-16 |
| 1219 | S4231 | Pilocarpine HCl | c5 | L1300-16 |
| 1220 | S4235 | Phenazopyridine HCl | d5 | L1300-16 |
| 1221 | S4237 | Primaquine Diphosphate | e5 | L1300-16 |
| 1222 | S4238 | Cepharanthine | f5 | L1300-16 |
| 1223 | S4239 | Bergapten | g5 | L1300-16 |
| 1224 | S4240 | Doxylamine Succinate | h5 | L1300-16 |
| 1225 | S4242 | Cetrimonium Bromide (CTAB) | a6 | L1300-16 |
| 1226 | S4243 | Deoxycorticosterone acetate | b6 | L1300-16 |
| 1227 | S4244 | Serotonin HCl | c6 | L1300-16 |
| 1228 | S4246 | Tranylcypromine (2-PCPA) HCl | d6 | L1300-16 |
| 1229 | S4247 | Prucalopride Succinate | e6 | L1300-16 |
| 1230 | S4248 | Bromfenac Sodium | f6 | L1300-16 |
| 1231 | S4249 | Flopropione | g6 | L1300-16 |
| 1232 | S4250 | Sulfamethoxypyridazine | h6 | L1300-16 |
| 1233 | S4252 | Mechlorethamine HCl | a7 | L1300-16 |
| 1234 | S4253 | Epinastine HCl | b7 | L1300-16 |
| 1235 | S4255 | Quinacrine 2HCl | c7 | L1300-16 |
| 1236 | S4256 | Buspirone HCl | d7 | L1300-16 |
| 1237 | S4257 | Alizapride HCl | e7 | L1300-16 |
| 1238 | S4258 | Luliconazole | f7 | L1300-16 |
| 1239 | S4259 | Vilazodone HCl | g7 | L1300-16 |
| 1240 | S4260 | Tamibarotene | h7 | L1300-16 |
| 1241 | S4263 | Efaproxiral Sodium | a8 | L1300-16 |
| 1242 | S4264 | Etofibrate | b8 | L1300-16 |
| 1243 | S4265 | Nicaraven | c8 | L1300-16 |
| 1244 | S4266 | Brimonidine Tartrate | d8 | L1300-16 |
| 1245 | S4267 | Diacerein | e8 | L1300-16 |
| 1246 | S4268 | Flufenamic acid | f8 | L1300-16 |
| 1247 | S4269 | Vinorelbine Tartrate | g8 | L1300-16 |
| 1248 | S4270 | Oxiracetam | h8 | L1300-16 |
| 1249 | S4274 | Rotigotine | a9 | L1300-16 |
| 1250 | S4277 | Bambuterol HCl | b9 | L1300-16 |
| 1251 | S4278 | Carteolol HCl | c9 | L1300-16 |
| 1252 | S4279 | Demeclocycline HCl | d9 | L1300-16 |
| 1253 | S4280 | Meclofenoxate (Centrophenoxine) HCl | e9 | L1300-16 |
| 1254 | S4281 | Tasimelteon | f9 | L1300-16 |
| 1255 | S4282 | Nelfinavir Mesylate | g9 | L1300-16 |
| 1256 | S4283 | Cyclobenzaprine HCl | h9 | L1300-16 |
| 1257 | S4284 | Chloroprocaine HCl | a10 | L1300-16 |
| 1258 | S4285 | Ospemifene | b10 | L1300-16 |
| 1259 | S4286 | Anidulafungin (LY303366) | c10 | L1300-16 |
| 1260 | S4288 | Chloroambucil | d10 | L1300-16 |
| 1261 | S4289 | Metoclopramide HCl | e10 | L1300-16 |
| 1262 | S4290 | Digoxin | f10 | L1300-16 |
| 1263 | S4291 | Labetalol HCl | g10 | L1300-16 |
| 1264 | S4292 | Diphenidol HCl | h10 | L1300-16 |
| 1265 | S4293 | Promethazine HCl | a11 | L1300-16 |
| 1266 | S4294 | Procainamide HCl | b11 | L1300-16 |
| 1267 | S4295 | Meclofenamate Sodium | c11 | L1300-16 |
| 1268 | S4296 | Salmeterol Xinafoate | d11 | L1300-16 |
| 1269 | S4297 | Mupirocin | e11 | L1300-16 |
| 1270 | S4299 | Dicoumarol | f11 | L1300-16 |
| 1271 | S4301 | (R)-(+)-Atenolol HCl | g11 | L1300-16 |
| 1272 | S4304 | Anisindione | h11 | L1300-16 |
| 1273 | S4305 | Anisotropine Methylbromide | a2 | L1300-17 |
| 1274 | S4307 | Auranofin | b2 | L1300-17 |
| 1275 | S4308 | Benzthiazide | c2 | L1300-17 |
| 1276 | S4309 | Bromocriptine Mesylate | d2 | L1300-17 |
| 1277 | S4312 | Carbadox | e2 | L1300-17 |
| 1278 | S4317 | Clorgyline HCl | f2 | L1300-17 |
| 1279 | S4322 | Disopyramide Phosphate | g2 | L1300-17 |
| 1280 | S4326 | Ethoxzolamide | h2 | L1300-17 |
| 1281 | S4330 | Isoetharine Mesylate | a3 | L1300-17 |
| 1282 | S4331 | Meclocycline Sulfosalicylate | b3 | L1300-17 |
| 1283 | S4332 | Medrysone | c3 | L1300-17 |
| 1284 | S4334 | Mesoridazine Besylate | d3 | L1300-17 |
| 1285 | S4335 | Metaproterenol Sulfate | e3 | L1300-17 |
| 1286 | S4336 | Metaraminol Bitartrate | f3 | L1300-17 |
| 1287 | S4338 | Methoxamine HCl | g3 | L1300-17 |
| 1288 | S4339 | Meticrane | h3 | L1300-17 |
| 1289 | S4343 | Oxethazaine | b4 | L1300-17 |
| 1290 | S4344 | Oxprenolol HCl | c4 | L1300-17 |
| 1291 | S4345 | Pentoxifylline | d4 | L1300-17 |
| 1292 | S4348 | Piromidic Acid | e4 | L1300-17 |
| 1293 | S4349 | Procyclidine HCl | f4 | L1300-17 |
| 1294 | S4351 | Ractopamine HCl | g4 | L1300-17 |
| 1295 | S4353 | Terfenadine | h4 | L1300-17 |
| 1296 | S4354 | Thiostrepton | a5 | L1300-17 |
| 1297 | S4357 | Tacrine HCl | b5 | L1300-17 |
| 1298 | S4358 | Pimozide | c5 | L1300-17 |
| 1299 | S4359 | Carbachol | d5 | L1300-17 |
| 1300 | S4361 | Cinoxacin | e5 | L1300-17 |
| 1301 | S4362 | Glafenine HCl | f5 | L1300-17 |
| 1302 | S4365 | Phthalylsulfacetamide | g5 | L1300-17 |
| 1303 | S4366 | Pinacidil | h5 | L1300-17 |
| 1304 | S4367 | Suxibuzone | a6 | L1300-17 |
| 1305 | S4368 | Carbenoxolone Sodium | b6 | L1300-17 |
| 1306 | S4373 | Dicyclomine HCl | d6 | L1300-17 |
| 1307 | S4375 | Mepenzolate Bromide | e6 | L1300-17 |
| 1308 | S4376 | Aceclidine HCl | f6 | L1300-17 |
| 1309 | S4377 | Imipramine HCl | g6 | L1300-17 |
| 1310 | S4381 | Proadifen HCl | h6 | L1300-17 |
| 1311 | S4382 | Pyrilamine Maleate | a7 | L1300-17 |
| 1312 | S4385 | Fosfomycin Tromethamine | b7 | L1300-17 |
| 1313 | S4387 | Bendroflumethiazide | c7 | L1300-17 |
| 1314 | S4388 | Bentiromide | d7 | L1300-17 |
| 1315 | S4389 | Bephenium Hydroxynaphthoate | e7 | L1300-17 |
| 1316 | S4390 | Brucine sulfate salt hydrate | f7 | L1300-17 |
| 1317 | S4391 | Camylofin Chlorhydrate | g7 | L1300-17 |
| 1318 | S4393 | Cephapirin Sodium | h7 | L1300-17 |
| 1319 | S4394 | Clofoctol | a8 | L1300-17 |
| 1320 | S4396 | Digoxigenin | b8 | L1300-17 |
| 1321 | S4397 | Diperodon HCl | c8 | L1300-17 |
| 1322 | S4402 | Oxeladin Citrate | d8 | L1300-17 |
| 1323 | S4404 | Pasiniazid | e8 | L1300-17 |
| 1324 | S4405 | Picrotoxinin | f8 | L1300-17 |
| 1325 | S4406 | Pindolol | g8 | L1300-17 |
| 1326 | S4415 | Misoprostol | h8 | L1300-17 |
| 1327 | S4416 | Trimipramine Maleate | a9 | L1300-17 |
| 1328 | S4420 | Mefloquine HCl | b9 | L1300-17 |
| 1329 | S4502 | Eltrombopag | c9 | L1300-17 |
| 1330 | S4504 | 6-Mercaptopurine (6-MP) Monohydrate | d9 | L1300-17 |
| 1331 | S4505 | Vinblastine sulfate | e9 | L1300-17 |
| 1332 | S4506 | Acetazolamide | f9 | L1300-17 |
| 1333 | S4509 | 4-Aminoantipyrine | g9 | L1300-17 |
| 1334 | S4510 | 4-Aminobenzoic acid | h9 | L1300-17 |
| 1335 | S4512 | Aceglutamide | a10 | L1300-17 |
| 1336 | S4514 | Acetylleucine | b10 | L1300-17 |
| 1337 | S4515 | Ademetionine disulfate tosylate | c10 | L1300-17 |
| 1338 | S4516 | (+)-Camphor | d10 | L1300-17 |
| 1339 | S4517 | Cefotaxime sodium | e10 | L1300-17 |
| 1340 | S4518 | Chloroxylenol | f10 | L1300-17 |
| 1341 | S4520 | 2-Aminoheptane | h10 | L1300-17 |
| 1342 | S4525 | Ethylparaben | a11 | L1300-17 |
| 1343 | S4526 | Fenbufen | b11 | L1300-17 |
| 1344 | S4527 | Fenofibric acid | c11 | L1300-17 |
| 1345 | S4528 | Furazolidone | d11 | L1300-17 |
| 1346 | S4532 | Iopamidol | e11 | L1300-17 |
| 1347 | S4535 | Methylene Blue | f11 | L1300-17 |
| 1348 | S4536 | Nitrofurantoin | g11 | L1300-17 |
| 1349 | S4538 | Pantoprazole sodium | h11 | L1300-17 |
| 1350 | S4539 | Salicylic acid | a2 | L1300-18 |
| 1351 | S4541 | Triclosan | b2 | L1300-18 |
| 1352 | S4542 | Trihexyphenidyl hydrochloride | c2 | L1300-18 |
| 1353 | S4543 | Trimetazidine dihydrochloride | d2 | L1300-18 |
| 1354 | S4544 | Urethane | e2 | L1300-18 |
| 1355 | S4546 | Xylitol | f2 | L1300-18 |
| 1356 | S4547 | 8-Hydroxyquinoline | g2 | L1300-18 |
| 1357 | S4548 | Aminoguanidine hydrochloride | h2 | L1300-18 |
| 1358 | S4550 | Azelaic acid | a3 | L1300-18 |
| 1359 | S4552 | Bithionol | b3 | L1300-18 |
| 1360 | S4553 | Bronopol | c3 | L1300-18 |
| 1361 | S4556 | Carzenide | d3 | L1300-18 |
| 1362 | S4558 | Citiolone | e3 | L1300-18 |
| 1363 | S4559 | Cloxiquine | f3 | L1300-18 |
| 1364 | S4561 | Danthron | g3 | L1300-18 |
| 1365 | S4562 | Dehydrocholic acid | h3 | L1300-18 |
| 1366 | S4563 | Diatrizoic acid | a4 | L1300-18 |
| 1367 | S4564 | Diethylcarbamazine citrate | b4 | L1300-18 |
| 1368 | S4565 | Diiodohydroxyquinoline | c4 | L1300-18 |
| 1369 | S4566 | DL-Panthenol | d4 | L1300-18 |
| 1370 | S4569 | Fluphenazine dihydrochloride | e4 | L1300-18 |
| 1371 | S4570 | Halothane | f4 | L1300-18 |
| 1372 | S4571 | Hexylresorcinol | g4 | L1300-18 |
| 1373 | S4574 | Piperazine | h4 | L1300-18 |
| 1374 | S4576 | Sulfabenzamide | a5 | L1300-18 |
| 1375 | S4577 | Terpin hydrate | b5 | L1300-18 |
| 1376 | S4578 | Tyloxapol | c5 | L1300-18 |
| 1377 | S4579 | Resorcinol | d5 | L1300-18 |
| 1378 | S4580 | Hydroquinone | e5 | L1300-18 |
| 1379 | S4581 | Triacetin | f5 | L1300-18 |
| 1380 | S4583 | Butamben | g5 | L1300-18 |
| 1381 | S4584 | Butylparaben | h5 | L1300-18 |
| 1382 | S4585 | Succinylsulfathiazole | a6 | L1300-18 |
| 1383 | S4588 | Docusate Sodium | b6 | L1300-18 |
| 1384 | S4589 | Amodiaquine dihydrochloride dihydrate | c6 | L1300-18 |
| 1385 | S4590 | Dithranol | d6 | L1300-18 |
| 1386 | S4591 | Nitroxoline | e6 | L1300-18 |
| 1387 | S4593 | Chlormadinone acetate | f6 | L1300-18 |
| 1388 | S4594 | Cephalothin | g6 | L1300-18 |
| 1389 | S4595 | Cefazolin Sodium | h6 | L1300-18 |
| 1390 | S4596 | Cefixime | a7 | L1300-18 |
| 1391 | S4597 | Lercanidipine hydrochloride | b7 | L1300-18 |
| 1392 | S4599 | Benzyl benzoate | c7 | L1300-18 |
| 1393 | S4600 | Benzyl alcohol | d7 | L1300-18 |
| 1394 | S4601 | Clioquinol | e7 | L1300-18 |
| 1395 | S4602 | Acetohydroxamic acid | f7 | L1300-18 |
| 1396 | S4603 | Gallic acid | g7 | L1300-18 |
| 1397 | S4604 | Levofloxacin hydrate | h7 | L1300-18 |
| 1398 | S4609 | Diflunisal | a8 | L1300-18 |
| 1399 | S4610 | Mebendazole | b8 | L1300-18 |
| 1400 | S4612 | Dapson | c8 | L1300-18 |
| 1401 | S4617 | Dextromethorphan hydrobromide hydrate | d8 | L1300-18 |
| 1402 | S4618 | Fenoldopam mesylate | e8 | L1300-18 |
| 1403 | S4619 | Itopride hydrochloride | f8 | L1300-18 |
| 1404 | S4620 | Cefuroxime sodium | g8 | L1300-18 |
| 1405 | S4623 | Methylbenactyzine Bromide | h8 | L1300-18 |
| 1406 | S4625 | Alcaftadine | a9 | L1300-18 |
| 1407 | S4626 | Ethosuximide | b9 | L1300-18 |
| 1408 | S4628 | (+/-)-Sulfinpyrazone | c9 | L1300-18 |
| 1409 | S4629 | Chlorotrianisene | d9 | L1300-18 |
| 1410 | S4630 | Diazoxide | e9 | L1300-18 |
| 1411 | S4631 | Prochlorperazine dimaleate salt | f9 | L1300-18 |
| 1412 | S4632 | Hexachlorophene | g9 | L1300-18 |
| 1413 | S4633 | Isosorbide Mononitrate | h9 | L1300-18 |
| 1414 | S4634 | Sodium sulfadiazine | a10 | L1300-18 |
| 1415 | S4635 | Cyproheptadine hydrochloride | b10 | L1300-18 |
| 1416 | S4636 | Teneligliptin hydrobromide | c10 | L1300-18 |
| 1417 | S4637 | Prasugrel Hydrochloride | d10 | L1300-18 |
| 1418 | S4638 | Desogestrel | e10 | L1300-18 |
| 1419 | S4639 | Brexpiprazole | f10 | L1300-18 |
| 1420 | S4640 | Lesinurad | g10 | L1300-18 |
| 1421 | S4641 | Tedizolid Phosphate | h10 | L1300-18 |
| 1422 | S4646 | Ciclesonide | a11 | L1300-18 |
| 1423 | S4647 | Cefmenoxime hydrochloride | b11 | L1300-18 |
| 1424 | S4648 | Dantrolene sodium hemiheptahydrate | c11 | L1300-18 |
| 1425 | S4649 | Atipamezole hydrochloride | d11 | L1300-18 |
| 1426 | S4650 | Atipamezole | e11 | L1300-18 |
| 1427 | S4651 | Etoricoxib | f11 | L1300-18 |
| 1428 | S4652 | Sulisobenzone | g11 | L1300-18 |
| 1429 | S4655 | Sulpiride | h11 | L1300-18 |
| 1430 | S4656 | Parecoxib | a2 | L1300-19 |
| 1431 | S4657 | Eslicarbazepine Acetate | b2 | L1300-19 |
| 1432 | S4658 | Hydroquinidine | c2 | L1300-19 |
| 1433 | S4660 | Glycopyrrolate | d2 | L1300-19 |
| 1434 | S4661 | Tiagabine hydrochloride | e2 | L1300-19 |
| 1435 | S4662 | Atazanavir | f2 | L1300-19 |
| 1436 | S4663 | Fusidate Sodium | g2 | L1300-19 |
| 1437 | S4664 | Molsidomine | h2 | L1300-19 |
| 1438 | S4665 | Rebeprazole sodium | a3 | L1300-19 |
| 1439 | S4666 | Sivelestat sodium tetrahydrate | b3 | L1300-19 |
| 1440 | S4667 | Lidocaine hydrochloride | c3 | L1300-19 |
| 1441 | S4668 | Procaine | d3 | L1300-19 |
| 1442 | S4669 | Benzocaine hydrochloride | e3 | L1300-19 |
| 1443 | S4673 | Etonogestrel | f3 | L1300-19 |
| 1444 | S4674 | Hydroxyprogesterone caproate | g3 | L1300-19 |
| 1445 | S4675 | Tiagabine | h3 | L1300-19 |
| 1446 | S4676 | Gluconolactone | a4 | L1300-19 |
| 1447 | S4678 | Povidone iodine | b4 | L1300-19 |
| 1448 | S4679 | Terazosin HCl | c4 | L1300-19 |
| 1449 | S4680 | Protirelin | d4 | L1300-19 |
| 1450 | S4682 | Loxoprofen | e4 | L1300-19 |
| 1451 | S4683 | Sildenafil Mesylate | f4 | L1300-19 |
| 1452 | S4685 | Efavirenz | g4 | L1300-19 |
| 1453 | S4686 | Vitamin E | h4 | L1300-19 |
| 1454 | S4687 | Rivastigmine | a5 | L1300-19 |
| 1455 | S4689 | Deoxycholic acid | b5 | L1300-19 |
| 1456 | S4690 | Escin | c5 | L1300-19 |
| 1457 | S4691 | Oxybenzone | d5 | L1300-19 |
| 1458 | S4693 | Guanfacine Hydrochloride | e5 | L1300-19 |
| 1459 | S4695 | D panthenol | f5 | L1300-19 |
| 1460 | S4696 | CarbinoxaMine Maleate | g5 | L1300-19 |
| 1461 | S4697 | Saxagliptin hydrate | h5 | L1300-19 |
| 1462 | S4698 | Vitamin K1 | a6 | L1300-19 |
| 1463 | S4699 | Etretinate | b6 | L1300-19 |
| 1464 | S4701 | 2-Deoxy-D-glucose | c6 | L1300-19 |
| 1465 | S4706 | Eugenol | d6 | L1300-19 |
| 1466 | S4707 | Oleic Acid | e6 | L1300-19 |
| 1467 | S4709 | Latanoprost | f6 | L1300-19 |
| 1468 | S4711 | Esculetin | g6 | L1300-19 |
| 1469 | S4714 | (-)-Menthol | h6 | L1300-19 |
| 1470 | S4716 | Evans Blue | a7 | L1300-19 |
| 1471 | S4717 | Isatin | b7 | L1300-19 |
| 1472 | S4718 | Acetylcholine iodide | c7 | L1300-19 |
| 1473 | S4722 | (+)-Catechin | d7 | L1300-19 |
| 1474 | S4723 | (-)Epicatechin | e7 | L1300-19 |
| 1475 | S4725 | Benzenesulfonamide | f7 | L1300-19 |
| 1476 | S4726 | Lauric Acid | g7 | L1300-19 |
| 1477 | S4727 | Cinnarizine | h7 | L1300-19 |
| 1478 | S4731 | Perphenazine | a8 | L1300-19 |
| 1479 | S4733 | Retigabine | b8 | L1300-19 |
| 1480 | S4734 | Retigabine 2HCl | c8 | L1300-19 |
| 1481 | S4735 | Salvianolic acid B | d8 | L1300-19 |
| 1482 | S4736 | Trapidil | e8 | L1300-19 |
| 1483 | S4737 | Psoralen | f8 | L1300-19 |
| 1484 | S4748 | Ondansetron Hydrochloride Dihydrate | g8 | L1300-19 |
| 1485 | S4749 | Citalopram HBr | h8 | L1300-19 |
| 1486 | S4750 | Sulfacetamide sodium salt hydrate | a9 | L1300-19 |
| 1487 | S4751 | Cisapride hydrate | b9 | L1300-19 |
| 1488 | S4752 | Corticosterone | c9 | L1300-19 |
| 1489 | S4754 | Betulin | d9 | L1300-19 |
| 1490 | S4757 | Dihydrotestosterone(DHT) | e9 | L1300-19 |
| 1491 | S4759 | p-Coumaric Acid | f9 | L1300-19 |
| 1492 | S4768 | Melibiose | g9 | L1300-19 |
| 1493 | S4769 | L-5-Hydroxytryptophan | h9 | L1300-19 |
| 1494 | S4776 | Harmaline | a10 | L1300-19 |
| 1495 | S4779 | Menadiol Diacetate | b10 | L1300-19 |
| 1496 | S4783 | Benzyl isothiocyanate | c10 | L1300-19 |
| 1497 | S4792 | N-Acetylneuraminic acid | d10 | L1300-19 |
| 1498 | S4794 | Drostanolone Propionate | e10 | L1300-19 |
| 1499 | S4795 | Trenbolone acetate | f10 | L1300-19 |
| 1500 | S4796 | Methandrostenolone | g10 | L1300-19 |
| 1501 | S4797 | Nicergoline | h10 | L1300-19 |
| 1502 | S4800 | Daminozide | a11 | L1300-19 |
| 1503 | S4803 | Thymidine | b11 | L1300-19 |
| 1504 | S4812 | Ceftizoxime | c11 | L1300-19 |
| 1505 | S4813 | Cefuroxime axetil | d11 | L1300-19 |
| 1506 | S4815 | L-Cysteine HCl | e11 | L1300-19 |
| 1507 | S4816 | Diatrizoate sodium | f11 | L1300-19 |
| 1508 | S4817 | Atenolol | g11 | L1300-19 |
| 1509 | S4819 | Saccharin | h11 | L1300-19 |
| 1510 | S4820 | Diastase | a2 | L1300-20 |
| 1511 | S4830 | Maltose | b2 | L1300-20 |
| 1512 | S4831 | Piperonyl butoxide | c2 | L1300-20 |
| 1513 | S4832 | Tolmetin | d2 | L1300-20 |
| 1514 | S4833 | Cefoxitin sodium | e2 | L1300-20 |
| 1515 | S4834 | Propantheline bromide | f2 | L1300-20 |
| 1516 | S4835 | Aceclofenac | g2 | L1300-20 |
| 1517 | S4836 | Nilutamide | h2 | L1300-20 |
| 1518 | S4837 | Ibudilast | a3 | L1300-20 |
| 1519 | S4838 | Acotiamide hydrochloride | b3 | L1300-20 |
| 1520 | S4839 | Mosapride | c3 | L1300-20 |
| 1521 | S4841 | Laurocapram | d3 | L1300-20 |
| 1522 | S4843 | Potassium acetate | e3 | L1300-20 |
| 1523 | S4844 | Cefcapene Pivoxil Hydrochloride | f3 | L1300-20 |
| 1524 | S4845 | Rabeprazole | g3 | L1300-20 |
| 1525 | S4846 | Meropenem Trihydrate | h3 | L1300-20 |
| 1526 | S4847 | Faropenem Sodium | a4 | L1300-20 |
| 1527 | S4848 | Dalbavancin | b4 | L1300-20 |
| 1528 | S4849 | Levocetirizine Dihydrochloride | c4 | L1300-20 |
| 1529 | S4850 | Flucloxacillin sodium | d4 | L1300-20 |
| 1530 | S4851 | Tafluprost | e4 | L1300-20 |
| 1531 | S4852 | Gadopentetate Dimeglumine | f4 | L1300-20 |
| 1532 | S4853 | Ecabet sodium | g4 | L1300-20 |
| 1533 | S4854 | Bedaquiline fumarate | h4 | L1300-20 |
| 1534 | S4856 | Iproniazid | a5 | L1300-20 |
| 1535 | S4859 | TriacetonaMine | b5 | L1300-20 |
| 1536 | S4860 | Indole-3-carboxylic acid | c5 | L1300-20 |
| 1537 | S4862 | Squalene | d5 | L1300-20 |
| 1538 | S4865 | Cefetamet pivoxil hydrochloride | e5 | L1300-20 |
| 1539 | S4866 | Nicarbazin | f5 | L1300-20 |
| 1540 | S4867 | Propacetamol hydrochloride | g5 | L1300-20 |
| 1541 | S4868 | Xanthinol Nicotinate | h5 | L1300-20 |
| 1542 | S4869 | Sulfamethoxazole sodium | a6 | L1300-20 |
| 1543 | S4870 | Cefodizime Sodium | b6 | L1300-20 |
| 1544 | S4871 | Pyridoxal 5-phosphate monohydrate | c6 | L1300-20 |
| 1545 | S4874 | Cefazedone | d6 | L1300-20 |
| 1546 | S4875 | Cephapirin Benzathine | e6 | L1300-20 |
| 1547 | S4876 | Robenidine Hydrochoride | f6 | L1300-20 |
| 1548 | S4877 | Eperisone hydrochloride | g6 | L1300-20 |
| 1549 | S4878 | Neticonazole Hydrochloride | h6 | L1300-20 |
| 1550 | S4880 | Cefathiamidine | a7 | L1300-20 |
| 1551 | S4881 | Calcium Dobesilate | b7 | L1300-20 |
| 1552 | S4883 | Lynestrenol | c7 | L1300-20 |
| 1553 | S4885 | Taurolidine | d7 | L1300-20 |
| 1554 | S4886 | Menbutone | e7 | L1300-20 |
| 1555 | S4887 | Nikethamide | f7 | L1300-20 |
| 1556 | S4889 | Perospirone hydrochloride | g7 | L1300-20 |
| 1557 | S4890 | Bifendate | h7 | L1300-20 |
| 1558 | S4893 | Cytosine | a8 | L1300-20 |
| 1559 | S4896 | Elagolix Sodium | b8 | L1300-20 |
| 1560 | S4899 | Sulfogaiacol | c8 | L1300-20 |
| 1561 | S4931 | Propiverine hydrochloride | d8 | L1300-20 |
| 1562 | S4932 | Proxyphylline | e8 | L1300-20 |
| 1563 | S4935 | Asunaprevir | g8 | L1300-20 |
| 1564 | S4939 | cis-Aconitic acid | h8 | L1300-20 |
| 1565 | S4940 | Maltol | a9 | L1300-20 |
| 1566 | S4949 | Nonanoic acid | b9 | L1300-20 |
| 1567 | S4952 | Fumaric acid | c9 | L1300-20 |
| 1568 | S4953 | Usnic acid | d9 | L1300-20 |
| 1569 | S4957 | Linalool | e9 | L1300-20 |
| 1570 | S4958 | Glycocholic acid | f9 | L1300-20 |
| 1571 | S4964 | Lactobionic acid | g9 | L1300-20 |
| 1572 | S4971 | Buparvaquone | h9 | L1300-20 |
| 1573 | S4973 | Iminostilbene | a10 | L1300-20 |
| 1574 | S4975 | Fimasartan | b10 | L1300-20 |
| 1575 | S4976 | Sulfalene(SMPZ) | c10 | L1300-20 |
| 1576 | S4977 | Efonidipine | d10 | L1300-20 |
| 1577 | S4978 | Azathramycin | e10 | L1300-20 |
| 1578 | S4980 | Anamorelin | f10 | L1300-20 |
| 1579 | S4983 | Sorbic acid | g10 | L1300-20 |
| 1580 | S4988 | Tiamulin fumarate | a11 | L1300-20 |
| 1581 | S4991 | Valpromide | b11 | L1300-20 |
| 1582 | S4994 | Methylcobalamin | c11 | L1300-20 |
| 1583 | S4996 | Tavaborole (AN-2690) | d11 | L1300-20 |
| 1584 | S4999 | Avermectin B1(Abamectin) | e11 | L1300-20 |
| 1585 | S5001 | Tofacitinib (CP-690550) Citrate | f11 | L1300-20 |
| 1586 | S5002 | Fingolimod (FTY720) HCl | g11 | L1300-20 |
| 1587 | S5003 | Tacrolimus (FK506) | h11 | L1300-20 |
| 1588 | S5004 | Pimecrolimus | a2 | L1300-21 |
| 1589 | S5005 | Cefotiam hydrochloride | b2 | L1300-21 |
| 1590 | S5006 | Teprenone | c2 | L1300-21 |
| 1591 | S5007 | Delamanid | d2 | L1300-21 |
| 1592 | S5009 | Brivudine | e2 | L1300-21 |
| 1593 | S5010 | Indometacin Sodium | f2 | L1300-21 |
| 1594 | S5012 | Octenidine Dihydrochloride | h2 | L1300-21 |
| 1595 | S5013 | Ioversol | a3 | L1300-21 |
| 1596 | S5014 | Crisaborole (AN2728) | b3 | L1300-21 |
| 1597 | S5015 | Simeprevir | c3 | L1300-21 |
| 1598 | S5016 | Isoprinosine | d3 | L1300-21 |
| 1599 | S5017 | Oxyclozanide | e3 | L1300-21 |
| 1600 | S5019 | Indobufen | f3 | L1300-21 |
| 1601 | S5020 | Tilorone dihydrochloride | g3 | L1300-21 |
| 1602 | S5023 | Nadolol | h3 | L1300-21 |
| 1603 | S5025 | Efinaconazole | a4 | L1300-21 |
| 1604 | S5027 | Mebeverine Hydrochloride | b4 | L1300-21 |
| 1605 | S5028 | 4-Aminopyridine | c4 | L1300-21 |
| 1606 | S5029 | Etofylline | d4 | L1300-21 |
| 1607 | S5030 | Dihydralazine sulphate | e4 | L1300-21 |
| 1608 | S5032 | Mephenesin | f4 | L1300-21 |
| 1609 | S5033 | Terconazole | g4 | L1300-21 |
| 1610 | S5034 | Melitracen hydrochloride | h4 | L1300-21 |
| 1611 | S5037 | Phenazine methosulfate | a5 | L1300-21 |
| 1612 | S5038 | Valethamate Bromide | b5 | L1300-21 |
| 1613 | S5039 | Actarit | c5 | L1300-21 |
| 1614 | S5040 | Tiamulin | d5 | L1300-21 |
| 1615 | S5041 | Difloxacin hydrochloride | e5 | L1300-21 |
| 1616 | S5042 | Bevantolol hydrochloride | f5 | L1300-21 |
| 1617 | S5043 | Benorylate | g5 | L1300-21 |
| 1618 | S5046 | Clonixin | h5 | L1300-21 |
| 1619 | S5049 | Thiocolchicoside | a6 | L1300-21 |
| 1620 | S5052 | Granisetron | b6 | L1300-21 |
| 1621 | S5054 | Rifamycin sodium salt | c6 | L1300-21 |
| 1622 | S5055 | Milbemycin Oxime | d6 | L1300-21 |
| 1623 | S5056 | Dinoprost tromethamine | e6 | L1300-21 |
| 1624 | S5058 | Revaprazan Hydrochloride | f6 | L1300-21 |
| 1625 | S5059 | Pixantrone Maleate | g6 | L1300-21 |
| 1626 | S5060 | Metadoxine | h6 | L1300-21 |
| 1627 | S5062 | Daclatasvir Digydrochloride | a7 | L1300-21 |
| 1628 | S5063 | Trelagliptin succinate | b7 | L1300-21 |
| 1629 | S5065 | Ganciclovir sodium | c7 | L1300-21 |
| 1630 | S5066 | Pramipexole dihydrochloride | d7 | L1300-21 |
| 1631 | S5067 | Losartan | e7 | L1300-21 |
| 1632 | S5069 | Dabrafenib Mesylate | f7 | L1300-21 |
| 1633 | S5070 | Mupirocin calcium | g7 | L1300-21 |
| 1634 | S5071 | Duloxetine | h7 | L1300-21 |
| 1635 | S5073 | Donepezil | b8 | L1300-21 |
| 1636 | S5074 | Argatroban Monohydrate | c8 | L1300-21 |
| 1637 | S5075 | Acotiamide | d8 | L1300-21 |
| 1638 | S5076 | Xipamide | e8 | L1300-21 |
| 1639 | S5077 | Regorafenib Monohydrate | f8 | L1300-21 |
| 1640 | S5078 | Osimertinib mesylate | g8 | L1300-21 |
| 1641 | S5079 | Sitagliptin | h8 | L1300-21 |
| 1642 | S5081 | Ceforanide | a9 | L1300-21 |
| 1643 | S5082 | Vitamin K2 | b9 | L1300-21 |
| 1644 | S5083 | Lentinan | c9 | L1300-21 |
| 1645 | S5084 | Carbazochrome | d9 | L1300-21 |
| 1646 | S5085 | Azamethiphos | e9 | L1300-21 |
| 1647 | S5086 | p-Anisaldehyde | f9 | L1300-21 |
| 1648 | S5087 | Tianeptine | g9 | L1300-21 |
| 1649 | S5091 | Geranyl acetate | h9 | L1300-21 |
| 1650 | S5092 | Febantel | a10 | L1300-21 |
| 1651 | S5093 | Rafoxanide | b10 | L1300-21 |
| 1652 | S5096 | SulfadiMethoxine sodium | c10 | L1300-21 |
| 1653 | S5100 | Pralidoxime Iodide | e10 | L1300-21 |
| 1654 | S5102 | Stachyose | f10 | L1300-21 |
| 1655 | S5103 | lutein | g10 | L1300-21 |
| 1656 | S5105 | Proanthocyanidins | h10 | L1300-21 |
| 1657 | S5106 | Lanolin | a11 | L1300-21 |
| 1658 | S5108 | Tylosin | b11 | L1300-21 |
| 1659 | S5109 | Ademetionine | c11 | L1300-21 |
| 1660 | S5112 | alpha-Arbutin | d11 | L1300-21 |
| 1661 | S5113 | Propyl gallate | e11 | L1300-21 |
| 1662 | S5114 | Hydroquinine | f11 | L1300-21 |
| 1663 | S5117 | Doramectin | g11 | L1300-21 |
| 1664 | S5119 | Olivetol | h11 | L1300-21 |
| 1665 | S5121 | Phytol | a2 | L1300-22 |
| 1666 | S5126 | Aleuritic Acid | b2 | L1300-22 |
| 1667 | S5132 | Casanthranol | c2 | L1300-22 |
| 1668 | S5134 | D(-)-Arabinose | d2 | L1300-22 |
| 1669 | S5138 | Citral | e2 | L1300-22 |
| 1670 | S5141 | Pyrithioxin | f2 | L1300-22 |
| 1671 | S5143 | Citropten | g2 | L1300-22 |
| 1672 | S5145 | Protoporphyrin IX | h2 | L1300-22 |
| 1673 | S5155 | Raffinose | a3 | L1300-22 |
| 1674 | S5157 | Thymol | b3 | L1300-22 |
| 1675 | S5159 | Doxycycline | c3 | L1300-22 |
| 1676 | S5163 | 7-Methoxycoumarin | d3 | L1300-22 |
| 1677 | S5167 | Benzyl cinnamate | e3 | L1300-22 |
| 1678 | S5174 | Kojic acid | f3 | L1300-22 |
| 1679 | S5176 | Fructose | g3 | L1300-22 |
| 1680 | S5177 | Naproxen | h3 | L1300-22 |
| 1681 | S5179 | Ropivacaine Mesilate | a4 | L1300-22 |
| 1682 | S5200 | Diaveridine | b4 | L1300-22 |
| 1683 | S5203 | Sulbutiamine | c4 | L1300-22 |
| 1684 | S5205 | Nilotinib hydrochloride | d4 | L1300-22 |
| 1685 | S5206 | Benzylpenicillin potassium | e4 | L1300-22 |
| 1686 | S5208 | Ciprofloxacin hydrochloride hydrate | f4 | L1300-22 |
| 1687 | S5209 | Enoxacin Sesquihydrate | g4 | L1300-22 |
| 1688 | S5210 | Sulfamethazine Sodium Salt | h4 | L1300-22 |
| 1689 | S5211 | 4-Aminosalicylic acid | a5 | L1300-22 |
| 1690 | S5213 | Piroctone Olamine | b5 | L1300-22 |
| 1691 | S5214 | Imidocarb dipropionate | c5 | L1300-22 |
| 1692 | S5215 | Abacavir | d5 | L1300-22 |
| 1693 | S5217 | Arformoterol Tartrate | e5 | L1300-22 |
| 1694 | S5223 | Phenylpiracetam | g5 | L1300-22 |
| 1695 | S5224 | Adrafinil | h5 | L1300-22 |
| 1696 | S5225 | Boldenone | a6 | L1300-22 |
| 1697 | S5227 | Erythromycin thiocyanate | b6 | L1300-22 |
| 1698 | S5228 | Minoxidil sulphate | c6 | L1300-22 |
| 1699 | S5229 | Isosorbide dinitrate | d6 | L1300-22 |
| 1700 | S5233 | Fenretinide | e6 | L1300-22 |
| 1701 | S5234 | Nintedanib Ethanesulfonate Salt | f6 | L1300-22 |
| 1702 | S5238 | Solifenacin | g6 | L1300-22 |
| 1703 | S5239 | Paroxetine mesylate | h6 | L1300-22 |
| 1704 | S5240 | lenvatinib Mesylate | a7 | L1300-22 |
| 1705 | S5241 | Lapatinib ditosylate monohydrate | b7 | L1300-22 |
| 1706 | S5243 | Ruxolitinib Phosphate | c7 | L1300-22 |
| 1707 | S5245 | Raltegravir potassium | d7 | L1300-22 |
| 1708 | S5246 | Entecavir | e7 | L1300-22 |
| 1709 | S5247 | Selamectin | f7 | L1300-22 |
| 1710 | S5250 | Darunavir | h7 | L1300-22 |
| 1711 | S5253 | Cisapride | a8 | L1300-22 |
| 1712 | S5254 | Dasatinib hydrochloride | b8 | L1300-22 |
| 1713 | S5255 | Nicardipine | c8 | L1300-22 |
| 1714 | S5256 | Benproperine phosphate | d8 | L1300-22 |
| 1715 | S5257 | Doxapram | e8 | L1300-22 |
| 1716 | S5258 | Revefenacin | f8 | L1300-22 |
| 1717 | S5261 | Ufenamate | g8 | L1300-22 |
| 1718 | S5266 | Stiripentol | h8 | L1300-22 |
| 1719 | S5273 | Canrenone | a9 | L1300-22 |
| 1720 | S5278 | Tedizolid | b9 | L1300-22 |
| 1721 | S5279 | Fendiline hydrochloride | c9 | L1300-22 |
| 1722 | S5281 | Dapiprazole Hydrochloride | d9 | L1300-22 |
| 1723 | S5282 | Lactitol monohydrate | e9 | L1300-22 |
| 1724 | S5284 | Adenosine 5'-monophosphate monohydrate | f9 | L1300-22 |
| 1725 | S5286 | Ramatroban | g9 | L1300-22 |
| 1726 | S5287 | Tiletamine Hydrochloride | h9 | L1300-22 |
| 1727 | S5288 | Estropipate | a10 | L1300-22 |
| 1728 | S5289 | Ceftezole | b10 | L1300-22 |
| 1729 | S5291 | sulfaisodimidine | c10 | L1300-22 |
| 1730 | S5292 | Diazolidinyl urea | d10 | L1300-22 |
| 1731 | S5293 | Nimustine Hydrochloride | e10 | L1300-22 |
| 1732 | S5295 | Lincomycin Hydrochloride Monohydrate | f10 | L1300-22 |
| 1733 | S5297 | Vidarabine monohydrate | g10 | L1300-22 |
| 1734 | S5298 | Amoxicillin trihydrate | h10 | L1300-22 |
| 1735 | S5299 | Tosufloxacin p-Toluenesulfonate Hydrate | a11 | L1300-22 |
| 1736 | S5301 | Carglumic Acid | b11 | L1300-22 |
| 1737 | S5302 | 6-Methoxy-2-naphthoic acid | c11 | L1300-22 |
| 1738 | S5305 | Bromisoval | d11 | L1300-22 |
| 1739 | S5309 | Propyphenazone (4-Isopropylantipyrine) | e11 | L1300-22 |
| 1740 | S5311 | Pyridoxal phosphate | f11 | L1300-22 |
| 1741 | S5320 | Octinoxate | g11 | L1300-22 |
| 1742 | S5322 | Sodium gualenate | h11 | L1300-22 |
| 1743 | S5325 | Nitisinone | a2 | L1300-23 |
| 1744 | S5326 | Dolasetron | b2 | L1300-23 |
| 1745 | S5327 | Meisoindigo | c2 | L1300-23 |
| 1746 | S5328 | Gamithromycin | d2 | L1300-23 |
| 1747 | S5331 | Ceftezole sodium | e2 | L1300-23 |
| 1748 | S5333 | Sulbenicillin Sodium | f2 | L1300-23 |
| 1749 | S5341 | Metroprolol succinate | g2 | L1300-23 |
| 1750 | S5343 | Vanillic acid | h2 | L1300-23 |
| 1751 | S5345 | Nerolidol | a3 | L1300-23 |
| 1752 | S5350 | Cefpodoxime proxetil | b3 | L1300-23 |
| 1753 | S5351 | Cefmetazole sodium | c3 | L1300-23 |
| 1754 | S5352 | Cefminox Sodium | d3 | L1300-23 |
| 1755 | S5353 | Cefpiramide sodium | e3 | L1300-23 |
| 1756 | S5356 | Ceftiofur | f3 | L1300-23 |
| 1757 | S5357 | Safinamide | g3 | L1300-23 |
| 1758 | S5358 | Regadenoson | h3 | L1300-23 |
| 1759 | S5359 | Butoconazole | a4 | L1300-23 |
| 1760 | S5360 | Diflorasone | b4 | L1300-23 |
| 1761 | S5361 | Bendazac | c4 | L1300-23 |
| 1762 | S5363 | Pikamilone | d4 | L1300-23 |
| 1763 | S5365 | Alogliptin | e4 | L1300-23 |
| 1764 | S5366 | Fipronil | f4 | L1300-23 |
| 1765 | S5367 | Ethyl Oleate | g4 | L1300-23 |
| 1766 | S5368 | Lactitol | h4 | L1300-23 |
| 1767 | S5369 | Ethoxyquin | a5 | L1300-23 |
| 1768 | S5371 | Ajmaline | b5 | L1300-23 |
| 1769 | S5372 | Methyl Aminolevulinate Hydrochloride | c5 | L1300-23 |
| 1770 | S5377 | Dibutyl phthalate | d5 | L1300-23 |
| 1771 | S5378 | Dimethyl phthalate | e5 | L1300-23 |
| 1772 | S5382 | Formate | f5 | L1300-23 |
| 1773 | S5385 | Imidafenacin | g5 | L1300-23 |
| 1774 | S5388 | Betrixaban | h5 | L1300-23 |
| 1775 | S5389 | Betrixaban maleate | a6 | L1300-23 |
| 1776 | S5392 | Mepivacaine | b6 | L1300-23 |
| 1777 | S5393 | Cyclofenil | c6 | L1300-23 |
| 1778 | S5395 | Phenolphthalein | d6 | L1300-23 |
| 1779 | S5397 | Chlorhexidine | e6 | L1300-23 |
| 1780 | S5398 | Nefazodone hydrochloride | f6 | L1300-23 |
| 1781 | S5399 | Chlorprothixene hydrochloride | g6 | L1300-23 |
| 1782 | S5401 | Tegaserod Maleate | h6 | L1300-23 |
| 1783 | S5402 | Dasabuvir(ABT-333) | a7 | L1300-23 |
| 1784 | S5403 | Ombitasvir (ABT-267) | b7 | L1300-23 |
| 1785 | S5404 | Paritaprevir (ABT-450) | c7 | L1300-23 |
| 1786 | S5405 | Propylparaben | d7 | L1300-23 |
| 1787 | S5406 | Sultamicillin Tosylate | e7 | L1300-23 |
| 1788 | S5407 | Squalane | f7 | L1300-23 |
| 1789 | S5408 | Isoprene | g7 | L1300-23 |
| 1790 | S5410 | Chloramphenicol sodium succinate | h7 | L1300-23 |
| 1791 | S5411 | Sultamicillin | a8 | L1300-23 |
| 1792 | S5413 | Ertugliflozin | b8 | L1300-23 |
| 1793 | S5414 | Diflucortolone valerate | c8 | L1300-23 |
| 1794 | S5416 | Metyrapone | e8 | L1300-23 |
| 1795 | S5418 | Parecoxib Sodium | f8 | L1300-23 |
| 1796 | S5419 | 1,4-Cineole | g8 | L1300-23 |
| 1797 | S5420 | Clindamycin alcoholate | h8 | L1300-23 |
| 1798 | S5424 | Carbaryl | a9 | L1300-23 |
| 1799 | S5428 | Promazine hydrochloride | b9 | L1300-23 |
| 1800 | S5430 | Metoprolol | c9 | L1300-23 |
| 1801 | S5435 | Quinacrine Dihydrochloride Dihydrate | d9 | L1300-23 |
| 1802 | S5440 | Berberine Sulfate | e9 | L1300-23 |
| 1803 | S5447 | Triprolidine Hydrochloride | f9 | L1300-23 |
| 1804 | S5450 | Sofalcone | g9 | L1300-23 |
| 1805 | S5452 | Sanguinarine chloride | h9 | L1300-23 |
| 1806 | S5453 | Hyperoside | a10 | L1300-23 |
| 1807 | S5454 | Saikosaponin D | b10 | L1300-23 |
| 1808 | S5457 | Curculigoside | c10 | L1300-23 |
| 1809 | S5459 | Aucubin | d10 | L1300-23 |
| 1810 | S5466 | Saikosaponin A | e10 | L1300-23 |
| 1811 | S5473 | Pivmecillinam hydrochloride | f10 | L1300-23 |
| 1812 | S5476 | Rolapitant | g10 | L1300-23 |
| 1813 | S5477 | Gefarnate | h10 | L1300-23 |
| 1814 | S5478 | Dantrolene sodium | a11 | L1300-23 |
| 1815 | S5479 | Cloperastine hydrochloride | b11 | L1300-23 |
| 1816 | S5480 | Clidinium Bromide | c11 | L1300-23 |
| 1817 | S5481 | Molindone hydrochloride | d11 | L1300-23 |
| 1818 | S5482 | Prilocaine hydrochloride | e11 | L1300-23 |
| 1819 | S5483 | Tribenzagan Hydrochloride | f11 | L1300-23 |
| 1820 | S5484 | Rimantadine Hydrochloride | g11 | L1300-23 |
| 1821 | S5485 | Desipramine Hydrochloride | h11 | L1300-23 |
| 1822 | S5486 | Fluorometholone | a2 | L1300-24 |
| 1823 | S5487 | Cefoperazone sodium | b2 | L1300-24 |
| 1824 | S5488 | Fluorescein | c2 | L1300-24 |
| 1825 | S5490 | Disopyramide | d2 | L1300-24 |
| 1826 | S5491 | Lomefloxacin | e2 | L1300-24 |
| 1827 | S5492 | Econazole | f2 | L1300-24 |
| 1828 | S5493 | Atropine sulfate | g2 | L1300-24 |
| 1829 | S5494 | Salbutamol | h2 | L1300-24 |
| 1830 | S5497 | Iopanoic acid | a3 | L1300-24 |
| 1831 | S5498 | Betahistine mesylate | b3 | L1300-24 |
| 1832 | S5500 | Amodiaquine hydrochloride | c3 | L1300-24 |
| 1833 | S5501 | Hydrocortisone acetate | d3 | L1300-24 |
| 1834 | S5502 | Ilaprazole sodium | e3 | L1300-24 |
| 1835 | S5504 | Ropivacaine | f3 | L1300-24 |
| 1836 | S5505 | 2’-deoxyuridine | g3 | L1300-24 |
| 1837 | S5506 | Vortioxetine | h3 | L1300-24 |
| 1838 | S5507 | Trimebutine maleate | a4 | L1300-24 |
| 1839 | S5508 | Dehydroepiandrosterone acetate | b4 | L1300-24 |
| 1840 | S5509 | 4-Aminophenol | c4 | L1300-24 |
| 1841 | S5515 | Penicillin G Procaine | d4 | L1300-24 |
| 1842 | S5527 | Salmeterol | e4 | L1300-24 |
| 1843 | S5528 | Acetophenone | f4 | L1300-24 |
| 1844 | S5530 | Geraniol | g4 | L1300-24 |
| 1845 | S5531 | Doripenem | h4 | L1300-24 |
| 1846 | S5532 | Erythromycin estolate | a5 | L1300-24 |
| 1847 | S5534 | Cinchocaine | b5 | L1300-24 |
| 1848 | S5535 | Moxifloxacin | c5 | L1300-24 |
| 1849 | S5537 | Tizanidine | d5 | L1300-24 |
| 1850 | S5538 | Tropisetron | e5 | L1300-24 |
| 1851 | S5539 | Olprinone | f5 | L1300-24 |
| 1852 | S5540 | Landiolol hydrochloride | g5 | L1300-24 |
| 1853 | S5541 | Dimetridazole | h5 | L1300-24 |
| 1854 | S5543 | 1, 10-Phenanthroline monohydrate | a6 | L1300-24 |
| 1855 | S5544 | D-Ribose | b6 | L1300-24 |
| 1856 | S5546 | Sulfacetamide | c6 | L1300-24 |
| 1857 | S5547 | Hydroxylammonium chloride | d6 | L1300-24 |
| 1858 | S5550 | Ethyl gallate | e6 | L1300-24 |
| 1859 | S5552 | Amenamevir | f6 | L1300-24 |
| 1860 | S5553 | Kasugamycin hydrochloride | g6 | L1300-24 |
| 1861 | S5554 | Lanatoside C | h6 | L1300-24 |
| 1862 | S5555 | Pamabrom | a7 | L1300-24 |
| 1863 | S5556 | (-)-Sparteine Sulfate | b7 | L1300-24 |
| 1864 | S5558 | D-Pantothenate Sodium | c7 | L1300-24 |
| 1865 | S5559 | Tetrahydropalmatine | d7 | L1300-24 |
| 1866 | S5560 | Midecamycin | e7 | L1300-24 |
| 1867 | S5561 | Ethacrynic Acid | f7 | L1300-24 |
| 1868 | S5562 | 2-Hydroxybenzyl alcohol | g7 | L1300-24 |
| 1869 | S5563 | Thioridazine hydrochloride | h7 | L1300-24 |
| 1870 | S5564 | Xylazine | a8 | L1300-24 |
| 1871 | S5565 | Triflupromazine hydrochloride | b8 | L1300-24 |
| 1872 | S5566 | Dapagliflozin propanediol monohydrate | c8 | L1300-24 |
| 1873 | S5567 | Trimethadione | d8 | L1300-24 |
| 1874 | S5568 | Tocofersolan | e8 | L1300-24 |
| 1875 | S5569 | Anisole | f8 | L1300-24 |
| 1876 | S5573 | Tilmicosin phosphate | g8 | L1300-24 |
| 1877 | S5580 | Arabic gum | h8 | L1300-24 |
| 1878 | S5581 | Olmesartan | a9 | L1300-24 |
| 1879 | S5582 | Cytarabine hydrochloride | b9 | L1300-24 |
| 1880 | S5583 | Cinnamyl acetate | c9 | L1300-24 |
| 1881 | S5585 | Citronellal | d9 | L1300-24 |
| 1882 | S5590 | Camphene | e9 | L1300-24 |
| 1883 | S5592 | Vitamin A | f9 | L1300-24 |
| 1884 | S5594 | α-Terpineol | g9 | L1300-24 |
| 1885 | S5596 | (1S)-(-)-α-Pinene | h9 | L1300-24 |
| 1886 | S5597 | (1R)-(-)-Menthyl acetate | a10 | L1300-24 |
| 1887 | S5598 | p-Cymene | b10 | L1300-24 |
| 1888 | S5599 | Sodium cholate | c10 | L1300-24 |
| 1889 | S5604 | Diphenylamine Hydrochloride | d10 | L1300-24 |
| 1890 | S5614 | (+)-Longifolene | e10 | L1300-24 |
| 1891 | S5618 | Hippuric acid | f10 | L1300-24 |
| 1892 | S5619 | Betahistine | g10 | L1300-24 |
| 1893 | S5621 | Cilastatin | h10 | L1300-24 |
| 1894 | S5623 | Bedaquiline | a11 | L1300-24 |
| 1895 | S5625 | Ammonium lactate | b11 | L1300-24 |
| 1896 | S5626 | Benzalkonium chloride | c11 | L1300-24 |
| 1897 | S5627 | Amsacrine hydrochloride | d11 | L1300-24 |
| 1898 | S5637 | Cefotiam Hexetil Hydrochloride | e11 | L1300-24 |
| 1899 | S5638 | Cefozopran hydrochloride | f11 | L1300-24 |
| 1900 | S5647 | Emedastine Difumarate | h11 | L1300-24 |
| 1901 | S5648 | Iguratimod | a2 | L1300-25 |
| 1902 | S5649 | Hydroxyzine pamoate | b2 | L1300-25 |
| 1903 | S5651 | Dexrazoxane | d2 | L1300-25 |
| 1904 | S5652 | Elbasvir | e2 | L1300-25 |
| 1905 | S5653 | Indigo carmine | f2 | L1300-25 |
| 1906 | S5654 | Indacaterol | g2 | L1300-25 |
| 1907 | S5655 | Venlafaxine | h2 | L1300-25 |
| 1908 | S5656 | Diphenylpyraline hydrochloride | a3 | L1300-25 |
| 1909 | S5657 | Ertapenem sodium | b3 | L1300-25 |
| 1910 | S5658 | Omeprazole Sodium | c3 | L1300-25 |
| 1911 | S5659 | Emedastine | d3 | L1300-25 |
| 1912 | S5661 | Tiaprofenic acid | e3 | L1300-25 |
| 1913 | S5662 | Ranitidine | f3 | L1300-25 |
| 1914 | S5663 | Minaprine dihydrochloride | g3 | L1300-25 |
| 1915 | S5664 | Orphenadrine Hydrochloride | h3 | L1300-25 |
| 1916 | S5665 | Drofenine Hydrochloride | a4 | L1300-25 |
| 1917 | S5666 | Moxisylyte hydrochloride | b4 | L1300-25 |
| 1918 | S5667 | Fruquintinib | c4 | L1300-25 |
| 1919 | S5669 | Isoxsuprine hydrochloride | d4 | L1300-25 |
| 1920 | S5670 | Chloropyramine hydrochloride | e4 | L1300-25 |
| 1921 | S5673 | Mivacurium chloride | f4 | L1300-25 |
| 1922 | S5679 | Dolasetron Mesylate | g4 | L1300-25 |
| 1923 | S5680 | (-)-Verbenone | h4 | L1300-25 |
| 1924 | S5682 | Methyl linolenate | a5 | L1300-25 |
| 1925 | S5683 | Isoproterenol sulfate dihydrate | b5 | L1300-25 |
| 1926 | S5685 | Desoximetasone | c5 | L1300-25 |
| 1927 | S5689 | Tropic acid | d5 | L1300-25 |
| 1928 | S5692 | Mexenone | e5 | L1300-25 |
| 1929 | S5693 | Levomilnacipran Hydrochloride | f5 | L1300-25 |
| 1930 | S5697 | Isopropamide Iodide | g5 | L1300-25 |
| 1931 | S5698 | Ketorolac tromethamine salt | h5 | L1300-25 |
| 1932 | S5700 | Phthalylsulfathiazole | a6 | L1300-25 |
| 1933 | S5701 | Alvimopan dihydrate | b6 | L1300-25 |
| 1934 | S5702 | Fenipentol | c6 | L1300-25 |
| 1935 | S5703 | Carvedilol Phosphate | d6 | L1300-25 |
| 1936 | S5705 | Raceanisodamine | e6 | L1300-25 |
| 1937 | S5709 | Norgestrel | f6 | L1300-25 |
| 1938 | S5710 | Ambroxol | g6 | L1300-25 |
| 1939 | S5713 | Pravastatin | a7 | L1300-25 |
| 1940 | S5714 | lurasidone | b7 | L1300-25 |
| 1941 | S5715 | atorvastatin | c7 | L1300-25 |
| 1942 | S5716 | Abemaciclib | d7 | L1300-25 |
| 1943 | S5717 | Acetohexamide | e7 | L1300-25 |
| 1944 | S5718 | Acrivastine | f7 | L1300-25 |
| 1945 | S5719 | Ceftizoxime sodium | g7 | L1300-25 |
| 1946 | S5720 | Glecaprevir | h7 | L1300-25 |
| 1947 | S5722 | Oxantel Pamoate | a8 | L1300-25 |
| 1948 | S5724 | Dichlorophene | b8 | L1300-25 |
| 1949 | S5726 | Triiodothyronine | c8 | L1300-25 |
| 1950 | S5727 | Tetryzoline | d8 | L1300-25 |
| 1951 | S5728 | Delapril Hydrochloride | e8 | L1300-25 |
| 1952 | S5729 | Fosfosal | f8 | L1300-25 |
| 1953 | S5730 | Alimemazine Tartrate | g8 | L1300-25 |
| 1954 | S5732 | Sebacic acid | h8 | L1300-25 |
| 1955 | S5733 | Stearic acid | a9 | L1300-25 |
| 1956 | S5734 | Midodrine hydrochloride | b9 | L1300-25 |
| 1957 | S5735 | Midodrine | c9 | L1300-25 |
| 1958 | S5736 | Benzathine penicilline | d9 | L1300-25 |
| 1959 | S5737 | Diclofenac Epolamine | e9 | L1300-25 |
| 1960 | S5739 | Nebivolol | f9 | L1300-25 |
| 1961 | S5740 | Palonosetron | g9 | L1300-25 |
| 1962 | S5741 | Quetiapine | h9 | L1300-25 |
| 1963 | S5742 | Deferoxamine mesylate | a10 | L1300-25 |
| 1964 | S5747 | Morantel tartrate | b10 | L1300-25 |
| 1965 | S5749 | Chlorpromazine | c10 | L1300-25 |
| 1966 | S5751 | Dihydroergotamine Mesylate | d10 | L1300-25 |
| 1967 | S5754 | Baricitinib phosphate | e10 | L1300-25 |
| 1968 | S5756 | Methyl Stearate | f10 | L1300-25 |
| 1969 | S5757 | Isoeugenol | g10 | L1300-25 |
| 1970 | S5759 | Methyl linoleate | h10 | L1300-25 |
| 1971 | S5761 | Citric acid | a11 | L1300-25 |
| 1972 | S5762 | Methyl Oleate | b11 | L1300-25 |
| 1973 | S5763 | D-Mannose | c11 | L1300-25 |
| 1974 | S5765 | Sodium Dehydrocholate | d11 | L1300-25 |
| 1975 | S5766 | Alfuzosin | e11 | L1300-25 |
| 1976 | S5767 | Aliskiren | f11 | L1300-25 |
| 1977 | S5768 | Fenoterol hydrobromide | g11 | L1300-25 |
| 1978 | S5769 | Fenoterol | h11 | L1300-25 |
| 1979 | S5776 | Proflavine | a2 | L1300-26 |
| 1980 | S5778 | Esmolol | b2 | L1300-26 |
| 1981 | S5779 | Trimetazidine | c2 | L1300-26 |
| 1982 | S5780 | Prazosin | d2 | L1300-26 |
| 1983 | S5781 | Raloxifene | e2 | L1300-26 |
| 1984 | S5782 | Doxazosin | f2 | L1300-26 |
| 1985 | S5783 | Montelukast | g2 | L1300-26 |
| 1986 | S5784 | Vancomycin | h2 | L1300-26 |
| 1987 | S5787 | Dronedarone | a3 | L1300-26 |
| 1988 | S5795 | Rasagiline | b3 | L1300-26 |
| 1989 | S5802 | Alprenolol hydrochloride | c3 | L1300-26 |
| 1990 | S5805 | Allopregnanolone | d3 | L1300-26 |
| 1991 | S5808 | Relugolix | e3 | L1300-26 |
| 1992 | S5812 | Choline Fenofibrate | f3 | L1300-26 |
| 1993 | S5816 | pyrvinium | g3 | L1300-26 |
| 1994 | S5821 | Linoleic acid | h3 | L1300-26 |
| 1995 | S5827 | Citronellyl acetate | a4 | L1300-26 |
| 1996 | S5842 | Cabergoline | b4 | L1300-26 |
| 1997 | S5843 | Cinitapride Hydrogen Tartrate | c4 | L1300-26 |
| 1998 | S5858 | Vilazodone | d4 | L1300-26 |
| 1999 | S5861 | Lercanidipine | e4 | L1300-26 |
| 2000 | S5862 | Metoclopramide | f4 | L1300-26 |
| 2001 | S5863 | Metronidazole Benzoate | g4 | L1300-26 |
| 2002 | S5869 | Cortisone | a5 | L1300-26 |
| 2003 | S5871 | Trans-Tranilast | b5 | L1300-26 |
| 2004 | S5872 | Estradiol dipropionate(17-Beta-Estradiol-3,17-Dipropionate) | c5 | L1300-26 |
| 2005 | S5873 | Scopolamine HBr trihydrate | d5 | L1300-26 |
| 2006 | S5874 | L-Carnitine hydrochloride | e5 | L1300-26 |
| 2007 | S5900 | Edrophonium chloride | f5 | L1300-26 |
| 2008 | S5901 | Canagliflozin hemihydrate | g5 | L1300-26 |
| 2009 | S5909 | Anagliptin | h5 | L1300-26 |
| 2010 | S5911 | Bictegravir | a6 | L1300-26 |
| 2011 | S5923 | Celiprolol hydrochloride | b6 | L1300-26 |
| 2012 | S5924 | Olanexidine Hydrochloride semihydrate | c6 | L1300-26 |
| 2013 | S5925 | Olodaterol hydrochloride | d6 | L1300-26 |
| 2014 | S5926 | Pitolisant hydrochloride | e6 | L1300-26 |
| 2015 | S5927 | Proguanil | f6 | L1300-26 |
| 2016 | S5935 | Alvimopan | g6 | L1300-26 |
| 2017 | S5950 | Fingolimod | h6 | L1300-26 |
| 2018 | S6003 | Ataluren (PTC124) | a7 | L1300-26 |
| 2019 | S6006 | Imidazole | b7 | L1300-26 |
| 2020 | S6007 | Bisphenol A | c7 | L1300-26 |
| 2021 | S6010 | Sodium L-lactate | d7 | L1300-26 |
| 2022 | S6014 | Isonicotinic acid | e7 | L1300-26 |
| 2023 | S6017 | p-Cresol | f7 | L1300-26 |
| 2024 | S6034 | p-Benzoquinone | g7 | L1300-26 |
| 2025 | S6035 | 2-Naphthol | h7 | L1300-26 |
| 2026 | S6041 | Dibutyl sebacate | a8 | L1300-26 |
| 2027 | S6047 | Lactose | b8 | L1300-26 |
| 2028 | S6049 | (+)-(S)-Carvone | c8 | L1300-26 |
| 2029 | S6052 | Saccharin sodium salt hydrate | d8 | L1300-26 |
| 2030 | S6053 | Phenylglyoxylic acid | e8 | L1300-26 |
| 2031 | S6055 | (S)-(−)-Limonene | f8 | L1300-26 |
| 2032 | S6058 | β-Caryophyllene | g8 | L1300-26 |
| 2033 | S6067 | 2,4-dichlorobenzyl alcohol | h8 | L1300-26 |
| 2034 | S6068 | Doxycycline monohydrate | a9 | L1300-26 |
| 2035 | S6073 | Diclofenac acid | b9 | L1300-26 |
| 2036 | S6079 | 2-Methylhexanoic acid | c9 | L1300-26 |
| 2037 | S6104 | (±)-α-Tocopherol | d9 | L1300-26 |
| 2038 | S6118 | Terpinen-4-ol | e9 | L1300-26 |
| 2039 | S6121 | Maltotriose | f9 | L1300-26 |
| 2040 | S6172 | Brucine sulfate heptahydrate | g9 | L1300-26 |
| 2041 | S6173 | Amylmetacresol | h9 | L1300-26 |
| 2042 | S6188 | p-Toluenesulfonic acid monohydrate | a10 | L1300-26 |
| 2043 | S6196 | 4-Nitrophenol | b10 | L1300-26 |
| 2044 | S6197 | Dimethylamine hydrochloride | c10 | L1300-26 |
| 2045 | S6199 | o-Cresol | d10 | L1300-26 |
| 2046 | S6202 | Butylated hydroxytoluene | e10 | L1300-26 |
| 2047 | S6207 | Levulinic acid | f10 | L1300-26 |
| 2048 | S6210 | Ethanolamine hydrochloride | g10 | L1300-26 |
| 2049 | S6211 | Furfural | h10 | L1300-26 |
| 2050 | S6215 | Phthalic acid | a11 | L1300-26 |
| 2051 | S6217 | o-Toluic acid | b11 | L1300-26 |
| 2052 | S6218 | Sodium lauryl sulfate | c11 | L1300-26 |
| 2053 | S6221 | Methyl cinnamate | d11 | L1300-26 |
| 2054 | S6223 | Triethyl citrate | e11 | L1300-26 |
| 2055 | S6226 | 4-Methyl-2-pentanone | f11 | L1300-26 |
| 2056 | S6231 | Methyl nicotinate | g11 | L1300-26 |
| 2057 | S6233 | Tartaric acid | h11 | L1300-26 |
| 2058 | S6234 | Pentadecanoic acid | a2 | L1300-27 |
| 2059 | S6235 | Sodium dehydroacetate | b2 | L1300-27 |
| 2060 | S6246 | Pyrrolidine | c2 | L1300-27 |
| 2061 | S6250 | L-Lactic acid | d2 | L1300-27 |
| 2062 | S6251 | Terephthalic acid | e2 | L1300-27 |
| 2063 | S6257 | N-Acetylglucosamine | f2 | L1300-27 |
| 2064 | S6258 | 1,2-Propanediol | g2 | L1300-27 |
| 2065 | S6262 | Ammonium formate | h2 | L1300-27 |
| 2066 | S6264 | Rutin hydrate | a3 | L1300-27 |
| 2067 | S6272 | Glycolic acid | b3 | L1300-27 |
| 2068 | S6275 | (−)-β-Pinene | c3 | L1300-27 |
| 2069 | S6281 | Sodium Thiocyanate | d3 | L1300-27 |
| 2070 | S6284 | Tetraethylammonium bromide | e3 | L1300-27 |
| 2071 | S6288 | Chlorhexidine diacetate | f3 | L1300-27 |
| 2072 | S6296 | Octanoic acid | g3 | L1300-27 |
| 2073 | S6305 | Catechol | h3 | L1300-27 |
| 2074 | S6316 | N-Acetyl-L-tyrosine | a4 | L1300-27 |
| 2075 | S6324 | 5-Methoxytryptamine | b4 | L1300-27 |
| 2076 | S6331 | m-Cresol | c4 | L1300-27 |
| 2077 | S6343 | D-Glucuronic acid | e4 | L1300-27 |
| 2078 | S6346 | L-(+)-Arabinose | f4 | L1300-27 |
| 2079 | S6352 | Androsterone | g4 | L1300-27 |
| 2080 | S7007 | Binimetinib (MEK162, ARRY-162, ARRY-438162) | h4 | L1300-27 |
| 2081 | S7028 | Duvelisib (IPI-145, INK1197) | a5 | L1300-27 |
| 2082 | S7059 | Tezacaftor?(VX-661) | b5 | L1300-27 |
| 2083 | S7077 | Cilengitide?trifluoroacetate | c5 | L1300-27 |
| 2084 | S7083 | Ceritinib (LDK378) | d5 | L1300-27 |
| 2085 | S7091 | Zotarolimus(ABT-578) | e5 | L1300-27 |
| 2086 | S7156 | Marimastat (BB-2516) | f5 | L1300-27 |
| 2087 | S7158 | abemaciclib (LY2835219) | g5 | L1300-27 |
| 2088 | S7160 | Glasdegib (PF-04449913) | h5 | L1300-27 |
| 2089 | S7179 | BAF312 (Siponimod) | a6 | L1300-27 |
| 2090 | S7280 | Edoxaban | b6 | L1300-27 |
| 2091 | S7297 | Osimertinib (AZD9291) | c6 | L1300-27 |
| 2092 | S7303 | Rilpivirine | d6 | L1300-27 |
| 2093 | S7397 | Sorafenib | e6 | L1300-27 |
| 2094 | S7411 | Ascomycin (FK520) | f6 | L1300-27 |
| 2095 | S7417 | Puromycin 2HCl | g6 | L1300-27 |
| 2096 | S7440 | Ribociclib (LEE011) | h6 | L1300-27 |
| 2097 | S7470 | Triapine | a7 | L1300-27 |
| 2098 | S7505 | (S)-crizotinib | b7 | L1300-27 |
| 2099 | S7513 | Trelagliptin | c7 | L1300-27 |
| 2100 | S7536 | Lorlatinib?(PF-6463922) | d7 | L1300-27 |
| 2101 | S7550 | Erythromycin Cyclocarbonate | e7 | L1300-27 |
| 2102 | S7559 | Darolutamide (ODM-201) | f7 | L1300-27 |
| 2103 | S7579 | Ledipasvir (GS5885) | g7 | L1300-27 |
| 2104 | S7594 | Abscisic Acid (Dormin） | h7 | L1300-27 |
| 2105 | S7625 | Niraparib (MK-4827) tosylate | a8 | L1300-27 |
| 2106 | S7633 | Lomitapide Mesylate | b8 | L1300-27 |
| 2107 | S7635 | Lomitapide | c8 | L1300-27 |
| 2108 | S7650 | Peficitinib (ASP015K, JNJ-54781532) | d8 | L1300-27 |
| 2109 | S7660 | Obeticholic Acid | e8 | L1300-27 |
| 2110 | S7663 | Ruboxistaurin (LY333531 HCl) | f8 | L1300-27 |
| 2111 | S7668 | Picropodophyllin (PPP) | g8 | L1300-27 |
| 2112 | S7678 | Sacubitril/valsartan (LCZ696) | h8 | L1300-27 |
| 2113 | S7721 | Monomethyl auristatin E (MMAE) | a9 | L1300-27 |
| 2114 | S7754 | Gilteritinib (ASP2215) | b9 | L1300-27 |
| 2115 | S7781 | Sunitinib | c9 | L1300-27 |
| 2116 | S7782 | Dasatinib Monohydrate | d9 | L1300-27 |
| 2117 | S7783 | Combretastatin A4 | e9 | L1300-27 |
| 2118 | S7784 | Fumagillin | f9 | L1300-27 |
| 2119 | S7786 | Erlotinib | g9 | L1300-27 |
| 2120 | S7787 | Docetaxel Trihydrate | h9 | L1300-27 |
| 2121 | S7810 | Afatinib (BIBW2992) Dimaleate | a10 | L1300-27 |
| 2122 | S7834 | Cyclo (-RGDfK) | b10 | L1300-27 |
| 2123 | S7844 | Cyclo(RGDyK) | c10 | L1300-27 |
| 2124 | S7852 | Eliglustat | d10 | L1300-27 |
| 2125 | S7856 | Tenofovir Alafenamide (GS-7340) | e10 | L1300-27 |
| 2126 | S7858 | Dibutyryl-cAMP (Bucladesine) | f10 | L1300-27 |
| 2127 | S7867 | Oleuropein | g10 | L1300-27 |
| 2128 | S7935 | Y-39983 HCl | h10 | L1300-27 |
| 2129 | S7954 | CP21R7 (CP21) | a11 | L1300-27 |
| 2130 | S7960 | Larotrectinib (LOXO-101) sulfate | b11 | L1300-27 |
| 2131 | S7975 | Favipiravir (T-705) | c11 | L1300-27 |
| 2132 | S7995 | Ripasudil (K-115) hydrochloride dihydrate | d11 | L1300-27 |
| 2133 | S8016 | Vonoprazan Fumarate (TAK-438) | e11 | L1300-27 |
| 2134 | S8021 | Vortioxetine (Lu AA21004) HBr | f11 | L1300-27 |
| 2135 | S8022 | Empagliflozin (BI 10773) | g11 | L1300-27 |
| 2136 | S8034 | Apremilast (CC-10004) | h11 | L1300-27 |
| 2137 | S8041 | Cobimetinib (GDC-0973, RG7420) | a2 | L1300-28 |
| 2138 | S8048 | Venetoclax (ABT-199, GDC-0199) | b2 | L1300-28 |
| 2139 | S8051 | Macitentan | c2 | L1300-28 |
| 2140 | S8067 | Vorapaxar | d2 | L1300-28 |
| 2141 | S8101 | CB-5083 | e2 | L1300-28 |
| 2142 | S8116 | Acalabrutinib (ACP-196) | f2 | L1300-28 |
| 2143 | S8133 | Resiquimod | g2 | L1300-28 |
| 2144 | S8134 | Radotinib | h2 | L1300-28 |
| 2145 | S8135 | Riociguat (BAY 63-2521) | a3 | L1300-28 |
| 2146 | S8136 | Sivelestat (ONO-5046) | b3 | L1300-28 |
| 2147 | S8144 | Halofuginone | c3 | L1300-28 |
| 2148 | S8146 | Mitomycin C | d3 | L1300-28 |
| 2149 | S8183 | Pimavanserin | e3 | L1300-28 |
| 2150 | S8195 | Oclacitinib?maleate | f3 | L1300-28 |
| 2151 | S8205 | Enasidenib (AG-221) | g3 | L1300-28 |
| 2152 | S8206 | Ivosidenib (AG-120) | h3 | L1300-28 |
| 2153 | S8266 | Melphalan | a4 | L1300-28 |
| 2154 | S8294 | Olmutinib (HM61713, BI 1482694) | b4 | L1300-28 |
| 2155 | S8401 | Erdafitinib (JNJ-42756493) | c4 | L1300-28 |
| 2156 | S8432 | Troglitazone (CS-045) | d4 | L1300-28 |
| 2157 | S8539 | TAS-102 | e4 | L1300-28 |
| 2158 | S8558 | Tofogliflozin(CSG 452) | f4 | L1300-28 |
| 2159 | S8565 | Omarigliptin (MK-3102) | g4 | L1300-28 |
| 2160 | S8567 | Tucidinostat (Chidamide) | h4 | L1300-28 |
| 2161 | S8594 | Tirofiban Hydrochloride | a5 | L1300-28 |
| 2162 | S8615 | Sodium dichloroacetate (DCA) | b5 | L1300-28 |
| 2163 | S8637 | Ipragliflozin (ASP1941) | c5 | L1300-28 |
| 2164 | S8726 | Anlotinib (AL3818) dihydrochloride | d5 | L1300-28 |
| 2165 | S9001 | Malic acid | e5 | L1300-28 |
| 2166 | S9002 | L-Fucose | f5 | L1300-28 |
| 2167 | S9003 | (R)-(-)-Mandelic acid | g5 | L1300-28 |
| 2168 | S9007 | 2'-Deoxyguanosine monohydrate | h5 | L1300-28 |
| 2169 | S9032 | Sanguinarine | a6 | L1300-28 |
| 2170 | S9042 | Wedelolactone | b6 | L1300-28 |
| 2171 | S9046 | Berberine | c6 | L1300-28 |
| 2172 | S9063 | Harringtonine | d6 | L1300-28 |
| 2173 | S9140 | Pulegone | e6 | L1300-28 |
| 2174 | S9141 | Berbamine | f6 | L1300-28 |
| 2175 | S9142 | Sparteine | g6 | L1300-28 |
| 2176 | S9143 | Ammonium Glycyrrhizate | h6 | L1300-28 |
| 2177 | S9193 | Aristolochic acid A | a7 | L1300-28 |
| 2178 | S9212 | Melamine | b7 | L1300-28 |
| 2179 | S9222 | Dipotassium glycyrrhizinate | c7 | L1300-28 |
| 2180 | S9227 | Sinensetin | d7 | L1300-28 |
| 2181 | S9240 | Isofraxidin | e7 | L1300-28 |
| 2182 | S9249 | Securinine | f7 | L1300-28 |
| 2183 | S9263 | 3-n-Butylphathlide | g7 | L1300-28 |
| 2184 | S9311 | Germacrone | h7 | L1300-28 |
| 2185 | S9321 | Topotecan | a8 | L1300-28 |
| 2186 | S9326 | Scopolamine | b8 | L1300-28 |
| 2187 | S9327 | Carboprost | c8 | L1300-28 |
| 2188 | S9329 | Orcinol | d8 | L1300-28 |
| 2189 | S9338 | Octyl gallate | e8 | L1300-28 |
| 2190 | S9346 | 1-Indanone | f8 | L1300-28 |
| 2191 | S9349 | D-(+)-Raffinose pentahydrate | g8 | L1300-28 |
| 2192 | S9351 | 2'-Deoxyadenosine monohydrate | h8 | L1300-28 |
| 2193 | S9354 | Oxalic acid | a9 | L1300-28 |
| 2194 | S9368 | ADP | b9 | L1300-28 |
| 2195 | S9373 | Neryl acetate | c9 | L1300-28 |
| 2196 | S9380 | 5,7-Dihydroxy-4-methylcoumarin | d9 | L1300-28 |
| 2197 | S9382 | Thymine | e9 | L1300-28 |
| 2198 | S9383 | Methyl palmitate | f9 | L1300-28 |
| 2199 | S9385 | Ligustilide | g9 | L1300-28 |
| 2200 | S9400 | Rhynchophylline | h9 | L1300-28 |
| 2201 | S9413 | Yangonin | a10 | L1300-28 |
| 2202 | S9451 | Uridine 5'-monophosphate | b10 | L1300-28 |
| 2203 | S9454 | Undecanoic acid | c10 | L1300-28 |
| 2204 | S9457 | Phensuximide | d10 | L1300-28 |
| 2205 | S9458 | Ergoloid Mesylates | e10 | L1300-28 |
| 2206 | S9459 | Mecamylamine Hydrochloride | f10 | L1300-28 |
| 2207 | S9460 | Ethotoin | g10 | L1300-28 |
| 2208 | S9461 | Benzonatate | h10 | L1300-28 |
| 2209 | S9463 | Demecarium Bromide | b11 | L1300-28 |
| 2210 | S9465 | Methysergide Maleate | c11 | L1300-28 |
| 2211 | S9466 | Methenamine Hippurate | d11 | L1300-28 |
| 2212 | S9467 | Thiothixene | e11 | L1300-28 |
| 2213 | S9469 | Haloperidol Decanoate | f11 | L1300-28 |
| 2214 | S9470 | Penbutolol Sulfate | g11 | L1300-28 |
| 2215 | S9472 | Oxtriphylline | h11 | L1300-28 |
| 2216 | S9500 | Valbenazine tosylate | a2 | L1300-29 |
| 2217 | S9502 | Madecassic acid | b2 | L1300-29 |
| 2218 | S1162 | PA-824 | c2 | L1300-29 |
| 2219 | S9506 | Danazol | d2 | L1300-29 |
| 2220 | S9507 | (−)-Norepinephrine | e2 | L1300-29 |
| 2221 | S9508 | Brimonidine | f2 | L1300-29 |
| 2222 | S9509 | Tiapride Hydrochloride | g2 | L1300-29 |
| 2223 | S9510 | Protriptyline hydrochloride | h2 | L1300-29 |
| 2224 | S9552 | Farrerol | a3 | L1300-29 |
| 2225 | S9560 | Anisodamine Hydrobromide | c3 | L1300-29 |
| 2226 | S9566 | Bepridil hydrochloride | d3 | L1300-29 |
| 2227 | S9567 | Indinavir Sulfate | e3 | L1300-29 |
| 2228 | S9568 | Sapropterin Dihydrochloride | f3 | L1300-29 |
| 2229 | S2736 | Fedratinib (SAR302503, TG101348) | g3 | L1300-29 |
| 2230 | S9015 | Homoharringtonine | h3 | L1300-29 |
| 2231 | S9019 | Quillaic acid | a4 | L1300-29 |
| 2232 | S9024 | AKBA | b4 | L1300-29 |
| 2233 | S9028 | Cimifugin | c4 | L1300-29 |
| 2234 | S9050 | Boldine | d4 | L1300-29 |
| 2235 | S9053 | Irisflorentin | e4 | L1300-29 |
| 2236 | S9086 | Dihydrocapsaicin | f4 | L1300-29 |
| 2237 | S9102 | Magnolin | g4 | L1300-29 |
| 2238 | S9110 | Morin | h4 | L1300-29 |
| 2239 | S9111 | Isorhamnetin | a5 | L1300-29 |
| 2240 | S9123 | Eriodictyol | b5 | L1300-29 |
| 2241 | S9171 | Harpagoside | c5 | L1300-29 |
| 2242 | S9183 | Ruscogenin | d5 | L1300-29 |
| 2243 | S9192 | Vitexin | e5 | L1300-29 |
| 2244 | S9200 | Pneumocandin B0 | f5 | L1300-29 |
| 2245 | S9224 | Dehydroandrographolide Succinate | g5 | L1300-29 |
| 2246 | S9248 | Homoorientin | h5 | L1300-29 |
| 2247 | S9270 | Dracohodin perochlorate | a6 | L1300-29 |
| 2248 | S9281 | Peimine | b6 | L1300-29 |
| 2249 | S9285 | Fargesin | c6 | L1300-29 |
| 2250 | S9288 | Casticin | d6 | L1300-29 |
| 2251 | S9294 | Gelsemine | e6 | L1300-29 |
| 2252 | S9416 | Triptonide | f6 | L1300-29 |
| 2253 | S1791 | Bacitracin Zinc | g6 | L1300-29 |
| 2254 | S1805 | Acetylcholine Chloride | h6 | L1300-29 |
| 2255 | S1820 | Clofibrate | a7 | L1300-29 |
| 2256 | S2072 | Seratrodast(AA-2414, ABT-001) | b7 | L1300-29 |
| 2257 | S2188 | Phenprocoumon | c7 | L1300-29 |
| 2258 | S2246 | Abiraterone Acetate | d7 | L1300-29 |
| 2259 | S2382 | Evodiamine | e7 | L1300-29 |
| 2260 | S2403 | Tetrandrine | f7 | L1300-29 |
| 2261 | S2611 | Ethisterone | g7 | L1300-29 |
| 2262 | S3030 | Niclosamide | h7 | L1300-29 |
| 2263 | S3153 | levalbuterol tartrate | a8 | L1300-29 |
| 2264 | S4199 | Antazoline HCl | b8 | L1300-29 |
| 2265 | S4316 | Clopamide | c8 | L1300-29 |
| 2266 | S4421 | Josamycin | d8 | L1300-29 |
| 2267 | S4672 | Dimenhydrinate | e8 | L1300-29 |
| 2268 | S4692 | Succimer | f8 | L1300-29 |
| 2269 | S4720 | Cefotaxime | g8 | L1300-29 |
| 2270 | S4744 | Anethole trithione | h8 | L1300-29 |
| 2271 | S4956 | Benzamidine HCl | a9 | L1300-29 |
| 2272 | S5847 | Ethacrynate Sodium | b9 | L1300-29 |
| 2273 | S5848 | Frovatriptan Succinate | c9 | L1300-29 |
| 2274 | S5849 | Cariprazine HCl | d9 | L1300-29 |
| 2275 | S5854 | Xylometazoline | e9 | L1300-29 |
| 2276 | S5856 | Trifluoperazine | f9 | L1300-29 |
| 2277 | S5857 | Trazodone | g9 | L1300-29 |
| 2278 | S5868 | DL-Menthol | h9 | L1300-29 |
| 2279 | S5887 | Khellin | a10 | L1300-29 |
| 2280 | S5921 | Cyclopentolate Hydrochloride | b10 | L1300-29 |
| 2281 | S5934 | (-)-Fenchone | c10 | L1300-29 |
| 2282 | S5936 | Cefazolin | d10 | L1300-29 |
| 2283 | S5938 | Benazepril | e10 | L1300-29 |
| 2284 | S5939 | Bendamustine | f10 | L1300-29 |
| 2285 | S5940 | Bepotastine | g10 | L1300-29 |
| 2286 | S5943 | Bromhexine | h10 | L1300-29 |
| 2287 | S5944 | Butenafine | a11 | L1300-29 |
| 2288 | S5948 | Amodiaquine | b11 | L1300-29 |
| 2289 | S5951 | Cefoxitin | c11 | L1300-29 |
| 2290 | S5952 | Baloxavir marboxil | d11 | L1300-29 |
| 2291 | S6400 | Glucosamine | e11 | L1300-29 |
| 2292 | S6402 | 2-Aminoethanethiol | f11 | L1300-29 |
| 2293 | S6403 | Chloramine-T | g11 | L1300-29 |
| 2294 | S6404 | Salicylamide | h11 | L1300-29 |
| 2295 | S6405 | Octisalate | a2 | L1300-30 |
| 2296 | S6407 | Tulobuterol hydrochloride | b2 | L1300-30 |
| 2297 | S6408 | Hexetidine | c2 | L1300-30 |
| 2298 | S6409 | Zucapsaicin | d2 | L1300-30 |
| 2299 | S6410 | Clemizole | e2 | L1300-30 |
| 2300 | S6411 | Nimorazole | f2 | L1300-30 |
| 2301 | S6432 | Cevimeline | g2 | L1300-30 |
| 2302 | S6433 | Udenafil | h2 | L1300-30 |
| 2303 | S6435 | Mequitazine | a3 | L1300-30 |
| 2304 | S6439 | Talniflumate | b3 | L1300-30 |
| 2305 | S6440 | Halazone | c3 | L1300-30 |
| 2306 | S6441 | Riboflavin Tetrabutyrate | d3 | L1300-30 |
| 2307 | S6442 | Chlorphenesin | e3 | L1300-30 |
| 2308 | S6443 | Chromium picolinate | f3 | L1300-30 |
| 2309 | S6444 | Triclocarban | g3 | L1300-30 |
| 2310 | S6447 | Carazolol | h3 | L1300-30 |
| 2311 | S6448 | Ibuprofen piconol | a4 | L1300-30 |
| 2312 | S6450 | Apronal | b4 | L1300-30 |
| 2313 | S6452 | Delavirdine (mesylate) | c4 | L1300-30 |
| 2314 | S6453 | Bicyclol | d4 | L1300-30 |
| 2315 | S6454 | Docosahexaenoic Acid | e4 | L1300-30 |
| 2316 | S6455 | Clebopride (malate) | f4 | L1300-30 |
| 2317 | S6456 | Glycyrrhetinic acid | g4 | L1300-30 |
| 2318 | S6457 | Flurbiprofen Axetil | h4 | L1300-30 |
| 2319 | S6459 | Nifurtimox | a5 | L1300-30 |
| 2320 | S6461 | Permethrin | b5 | L1300-30 |
| 2321 | S6462 | Amezinium (methylsulfate) | c5 | L1300-30 |
| 2322 | S6465 | Tafamidis | d5 | L1300-30 |
| 2323 | S6467 | Fosfluconazole | e5 | L1300-30 |
| 2324 | S6468 | Trandolapril | f5 | L1300-30 |
| 2325 | S6469 | Thonzylamine | g5 | L1300-30 |
| 2326 | S6470 | Fluralaner | h5 | L1300-30 |
| 2327 | S6472 | Tinoridine hydrochloride | a6 | L1300-30 |
| 2328 | S6473 | Pinaverium bromide | b6 | L1300-30 |
| 2329 | S6476 | Eicosapentaenoic Acid | c6 | L1300-30 |
| 2330 | S6477 | Octodrine (2-Amino-6-methylheptan) | d6 | L1300-30 |
| 2331 | S6478 | Fipexide hydrochloride | e6 | L1300-30 |
| 2332 | S6482 | Fosphenytoin (disodium) | f6 | L1300-30 |
| 2333 | S6484 | uridine triacetate | g6 | L1300-30 |
| 2334 | S6487 | fluticasone furoate | h6 | L1300-30 |
| 2335 | S6489 | Lasmiditan succinate | a7 | L1300-30 |
| 2336 | S6492 | Doravirine (MK-1439) | b7 | L1300-30 |
| 2337 | S6581 | Fosamprenavir calcium salt | c7 | L1300-30 |
| 2338 | S6582 | Ozenoxacin | d7 | L1300-30 |
| 2339 | S6583 | Apraclonidine HCI | e7 | L1300-30 |
| 2340 | S6587 | clobetasone butyrate | f7 | L1300-30 |
| 2341 | S6588 | Methylprednisolone hemisuccinate | g7 | L1300-30 |
| 2342 | S6589 | norethisterone enanthate | h7 | L1300-30 |
| 2343 | S6590 | Hydrocortisone butyrate | a8 | L1300-30 |
| 2344 | S6601 | (±)-Equol | b8 | L1300-30 |
| 2345 | S6611 | Cyclothiazide | c8 | L1300-30 |
| 2346 | S6614 | Fursultiamine | d8 | L1300-30 |
| 2347 | S6624 | Avatrombopag | e8 | L1300-30 |
| 2348 | S6626 | Brequinar | f8 | L1300-30 |
| 2349 | S6631 | Belotecan (CKD-602) hydrochloride | g8 | L1300-30 |
| 2350 | S6729 | Besifovir | h8 | L1300-30 |
| 2351 | S6742 | Etofenamate | a9 | L1300-30 |
| 2352 | S8871 | Omadacycline tosylate | b9 | L1300-30 |
| 2353 | S8873 | Letermovir(AIC246) | c9 | L1300-30 |
| 2354 | S9519 | Fadrozole | d9 | L1300-30 |
| 2355 | S2883 | 4-Aminohippuric Acid | e9 | L1300-30 |
| 2356 | S3046 | Azilsartan | f9 | L1300-30 |
| 2357 | S4073 | Sodium 4-Aminosalicylate | g9 | L1300-30 |
| 2358 | S4303 | 9-Aminoacridine | h9 | L1300-30 |
| 2359 | S4507 | 17-Hydroxyprogesterone | a10 | L1300-30 |
| 2360 | S4622 | 3,4-Diaminopyridine | b10 | L1300-30 |
| 2361 | S4624 | 5,5-Dimethyloxazolidine-2,4-dione | c10 | L1300-30 |
| 2362 | S5130 | Taurocholic acid sodium salt hydrate | d10 | L1300-30 |
| 2363 | S7025 | Embelin | e10 | L1300-30 |
| 2364 | S7818 | Pexidartinib (PLX3397) | f10 | L1300-30 |
| 2365 | S7998 | Entrectinib (RXDX-101) | g10 | L1300-30 |
| 2366 | S8540 | Voxelotor(GBT440, GTx011) | h10 | L1300-30 |
| 2367 | S2100 | Temocapril | a11 | L1300-30 |
| 2368 | S2307 | Roquinimex | b11 | L1300-30 |
| 2369 | S2426 | Tirapazamine | c11 | L1300-30 |
| 2370 | S2428 | Isopropyl myristate | d11 | L1300-30 |
| 2371 | S2464 | Sevoflurane | e11 | L1300-30 |
| 2372 | S6657 | 2-Aminoethyl Diphenylborinate (2-APB) | f11 | L1300-30 |
| 2373 | S6676 | Ebselen | g11 | L1300-30 |
| 2374 | S5617 | Myristic Acid | h11 | L1300-30 |
| 2375 | S0436 | Pleconaril | a2 | L1300-31 |
| 2376 | S2288 | Rubitecan | b2 | L1300-31 |
| 2377 | S2447 | Ansamitocin p-3 (Maytansinol isobutyrate, NSC292222) | c2 | L1300-31 |
| 2378 | S6829 | Dithiothreitol (DTT) | d2 | L1300-31 |
| 2379 | S6848 | 3'-Fluoro-3'-deoxythymidine (Alovudine) | e2 | L1300-31 |
| 2380 | S8964 | Actinomycin D (Dactinomycin) | f2 | L1300-31 |
| 2381 | S1008 | Selumetinib (AZD6244) | g2 | L1300-31 |
| 2382 | S1245 | Latrepirdine 2HCl | h2 | L1300-31 |
| 2383 | S1526 | Quizartinib (AC220) | a3 | L1300-31 |
| 2384 | S1591 | Bestatin | b3 | L1300-31 |
| 2385 | S1928 | Alibendol | c3 | L1300-31 |
| 2386 | S2082 | Adiphenine HCl | d3 | L1300-31 |
| 2387 | S2286 | Cyclosporin A | e3 | L1300-31 |
| 2388 | S3687 | Urea | f3 | L1300-31 |
| 2389 | S3797 | Helicide | g3 | L1300-31 |
| 2390 | S4521 | DEET | h3 | L1300-31 |
| 2391 | S4522 | Dehydroacetic acid | a4 | L1300-31 |
| 2392 | S4534 | 6-Acetamidohexanoic acid | b4 | L1300-31 |
| 2393 | S5641 | Rhodamine B | c4 | L1300-31 |
| 2394 | S6011 | Acetamide | d4 | L1300-31 |
| 2395 | S6065 | Congo Red | e4 | L1300-31 |
| 2396 | S6152 | Acid Red 27 | f4 | L1300-31 |
| 2397 | S7456 | Osilodrostat (LCI699) | g4 | L1300-31 |
| 2398 | S7952 | Ozanimod (RPC1063) | h4 | L1300-31 |
| 2399 | S7953 | ETC-1002 | a5 | L1300-31 |
| 2400 | S8103 | Sotagliflozin (LX4211) | b5 | L1300-31 |
| 2401 | S8791 | Zanubrutinib (BGB-3111) | c5 | L1300-31 |
| 2402 | S9452 | 10-Undecenoic acid | d5 | L1300-31 |
| 2403 | S6593 | Methylprednisolone sodium succinate | e5 | L1300-31 |
| 2404 | S8781 | Selpercatinib (LOXO-292, ARRY-192) | f5 | L1300-31 |
| 2405 | S6659 | Rimegepant (BMS-927711) | g5 | L1300-31 |
| 2406 | S6830 | o-Phenanthroline | h5 | L1300-31 |
| 2407 | S0074 | Relebactam | a6 | L1300-31 |
| 2408 | S0088 | Pemigatinib (INCB054828) | b6 | L1300-31 |
| 2409 | S0325 | Treprostinil sodium | c6 | L1300-31 |
| 2410 | S0398 | Astemizole | d6 | L1300-31 |
| 2411 | S0718 | AG-120 (racemic) | e6 | L1300-31 |
| 2412 | S0994 | Ipragliflozin L-Proline | f6 | L1300-31 |
| 2413 | S2440 | Vindesine sulfate | g6 | L1300-31 |
| 2414 | S3217 | Brilliant Blue G | h6 | L1300-31 |
| 2415 | S3220 | Trigonelline | a7 | L1300-31 |
| 2416 | S3233 | Emetine hydrochloride | b7 | L1300-31 |
| 2417 | S3239 | Emetine Dihydrochloride | c7 | L1300-31 |
| 2418 | S3273 | Hypericin | d7 | L1300-31 |
| 2419 | S3301 | Cynarin | e7 | L1300-31 |
| 2420 | S6852 | Gossypol | f7 | L1300-31 |
| 2421 | S6887 | Clozapine N-oxide | g7 | L1300-31 |
| 2422 | S6889 | Monomethyl Fumarate | h7 | L1300-31 |
| 2423 | S6896 | Clascoterone | a8 | L1300-31 |
| 2424 | S6906 | Capric acid | b8 | L1300-31 |
| 2425 | S9641 | Pibrentasvir (ABT-530) | c8 | L1300-31 |
| 2426 | S1037 | Perifosine (KRX-0401) | a2 | L1300-32 |
| 2427 | S1116 | Palbociclib (PD-0332991) HCl | b2 | L1300-32 |
| 2428 | S1135 | Pemetrexed | c2 | L1300-32 |
| 2429 | S1149 | Gemcitabine HCl | d2 | L1300-32 |
| 2430 | S1215 | Carboplatin | e2 | L1300-32 |
| 2431 | S1236 | Leucovorin Calcium Pentahydrate | f2 | L1300-32 |
| 2432 | S1311 | Pamidronate Disodium | g2 | L1300-32 |
| 2433 | S1338 | Gabapentin HCl | h2 | L1300-32 |
| 2434 | S1339 | Galanthamine HBr | a3 | L1300-32 |
| 2435 | S1345 | Granisetron HCl | b3 | L1300-32 |
| 2436 | S1370 | Biapenem | c3 | L1300-32 |
| 2437 | S1375 | Dorzolamide HCl | d3 | L1300-32 |
| 2438 | S1384 | Mizoribine | e3 | L1300-32 |
| 2439 | S1395 | Polymyxin B sulphate | f3 | L1300-32 |
| 2440 | S1399 | Teicoplanin | g3 | L1300-32 |
| 2441 | S1440 | Varenicline Tartrate | h3 | L1300-32 |
| 2442 | S1506 | Perindopril Erbumine | b4 | L1300-32 |
| 2443 | S1516 | Cidofovir | c4 | L1300-32 |
| 2444 | S1518 | Ibuprofen Lysine | d4 | L1300-32 |
| 2445 | S1579 | Palbociclib (PD0332991) Isethionate | e4 | L1300-32 |
| 2446 | S1624 | Alendronate sodium trihydrate | f4 | L1300-32 |
| 2447 | S1648 | Cytarabine | g4 | L1300-32 |
| 2448 | S1749 | L-Glutamine | h4 | L1300-32 |
| 2449 | S1751 | Gadodiamide Hydrate | a5 | L1300-32 |
| 2450 | S1826 | Nedaplatin | b5 | L1300-32 |
| 2451 | S1853 | Penicillamine | c5 | L1300-32 |
| 2452 | S1857 | Etidronate | d5 | L1300-32 |
| 2453 | S1875 | Tranexamic Acid | e5 | L1300-32 |
| 2454 | S1939 | Levamisole hydrochloride | f5 | L1300-32 |
| 2455 | S1984 | Ticlopidine HCl | g5 | L1300-32 |
| 2456 | S1985 | ATP | h5 | L1300-32 |
| 2457 | S1995 | Procarbazine HCl | a6 | L1300-32 |
| 2458 | S1998 | D-Cycloserine | b6 | L1300-32 |
| 2459 | S1999 | Sodium butyrate | c6 | L1300-32 |
| 2460 | S2008 | Taurine | d6 | L1300-32 |
| 2461 | S2048 | Clindamycin Phosphate | e6 | L1300-32 |
| 2462 | S2076 | Lisinopril dihydrate | f6 | L1300-32 |
| 2463 | S2095 | Fosinopril Sodium | g6 | L1300-32 |
| 2464 | S2129 | Fudosteine | h6 | L1300-32 |
| 2465 | S2133 | Gabapentin | a7 | L1300-32 |
| 2466 | S2315 | Kanamycin sulfate | b7 | L1300-32 |
| 2467 | S2416 | Chondroitin sulfate | c7 | L1300-32 |
| 2468 | S2462 | Donepezil HCl | d7 | L1300-32 |
| 2469 | S2490 | Neostigmine Bromide | e7 | L1300-32 |
| 2470 | S2507 | Salbutamol Sulfate | f7 | L1300-32 |
| 2471 | S2514 | Tobramycin | g7 | L1300-32 |
| 2472 | S2518 | NAD+ | h7 | L1300-32 |
| 2473 | S2527 | Methacycline HCl | a8 | L1300-32 |
| 2474 | S2539 | Lomefloxacin HCl | b8 | L1300-32 |
| 2475 | S2560 | Amiloride HCl dihydrate | c8 | L1300-32 |
| 2476 | S2563 | Oxacillin sodium monohydrate | d8 | L1300-32 |
| 2477 | S2568 | Neomycin sulfate | e8 | L1300-32 |
| 2478 | S2572 | Streptomycin sulfate | f8 | L1300-32 |
| 2479 | S2575 | Vancomycin HCl | g8 | L1300-32 |
| 2480 | S2908 | Hygromycin B | h8 | L1300-32 |
| 2481 | S3007 | Zanamivir | a9 | L1300-32 |
| 2482 | S3013 | Plerixafor 8HCl (AMD3100 8HCl) | b9 | L1300-32 |
| 2483 | S3028 | Geneticin (G418 Sulfate) | c9 | L1300-32 |
| 2484 | S3048 | Solifenacin succinate | d9 | L1300-32 |
| 2485 | S3050 | Palonosetron HCl | e9 | L1300-32 |
| 2486 | S3056 | Miltefosine | f9 | L1300-32 |
| 2487 | S3058 | Danofloxacin Mesylate | g9 | L1300-32 |
| 2488 | S3065 | Amikacin disulfate | h9 | L1300-32 |
| 2489 | S3072 | (R)-baclofen | a10 | L1300-32 |
| 2490 | S3073 | Caspofungin Acetate | b10 | L1300-32 |
| 2491 | S3102 | Creatinine | c10 | L1300-32 |
| 2492 | S3122 | Amikacin hydrate | d10 | L1300-32 |
| 2493 | S3146 | Tripelennamine HCl | e10 | L1300-32 |
| 2494 | S3148 | Ibandronate sodium | f10 | L1300-32 |
| 2495 | S3165 | Abacavir sulfate | g10 | L1300-32 |
| 2496 | S3174 | L-Arginine HCl (L-Arg) | h10 | L1300-32 |
| 2497 | S3211 | Thiamine HCl (Vitamin B1) | a11 | L1300-32 |
| 2498 | S3619 | Citicoline sodium | b11 | L1300-32 |
| 2499 | S3642 | Cesium chloride | c11 | L1300-32 |
| 2500 | S3649 | Ceftazidime | d11 | L1300-32 |
| 2501 | S3650 | Penicillin V potassium salt | e11 | L1300-32 |
| 2502 | S3662 | Pirenzepine dihydrochloride | f11 | L1300-32 |
| 2503 | S3667 | Imipenem | g11 | L1300-32 |
| 2504 | S3737 | Cangrelor Tetrasodium | a2 | L1300-33 |
| 2505 | S3748 | Acamprosate Calcium | b2 | L1300-33 |
| 2506 | S3752 | isoleucine | c2 | L1300-33 |
| 2507 | S3753 | L-Leucine | d2 | L1300-33 |
| 2508 | S3798 | L-Citrulline | e2 | L1300-33 |
| 2509 | S3852 | L-Theanine | f2 | L1300-33 |
| 2510 | S3861 | Sodium Demethylcantharidate | g2 | L1300-33 |
| 2511 | S3953 | L-Lysine hydrochloride | h2 | L1300-33 |
| 2512 | S3963 | DL-Glutamine | a3 | L1300-33 |
| 2513 | S3973 | L-SelenoMethionine | b3 | L1300-33 |
| 2514 | S4008 | Pemirolast potassium | c3 | L1300-33 |
| 2515 | S4013 | Sodium Monofluorophosphate | d3 | L1300-33 |
| 2516 | S4027 | Flavoxate HCl | e3 | L1300-33 |
| 2517 | S4028 | Dexamethasone Sodium Phosphate | f3 | L1300-33 |
| 2518 | S4029 | Colistin Sulfate | g3 | L1300-33 |
| 2519 | S4030 | Gentamicin Sulfate | h3 | L1300-33 |
| 2520 | S4052 | Netilmicin Sulfate | a4 | L1300-33 |
| 2521 | S4093 | Bismuth Subcitrate Potassium | b4 | L1300-33 |
| 2522 | S4094 | Tetramisole HCl | c4 | L1300-33 |
| 2523 | S4108 | Clodronate Disodium | d4 | L1300-33 |
| 2524 | S4117 | Histamine Phosphate | e4 | L1300-33 |
| 2525 | S4121 | Succinylcholine Chloride Dihydrate | f4 | L1300-33 |
| 2526 | S4127 | Terbutaline Sulfate | g4 | L1300-33 |
| 2527 | S4137 | Eprazinone 2HCl | h4 | L1300-33 |
| 2528 | S4144 | Amprolium HCl | a5 | L1300-33 |
| 2529 | S4146 | Bacitracin | b5 | L1300-33 |
| 2530 | S4157 | Chloroquine Phosphate | c5 | L1300-33 |
| 2531 | S4158 | Ceftriaxone Sodium Trihydrate | d5 | L1300-33 |
| 2532 | S4174 | Sodium Gluconate | e5 | L1300-33 |
| 2533 | S4180 | Nefopam HCl | f5 | L1300-33 |
| 2534 | S4183 | Paromomycin Sulfate | g5 | L1300-33 |
| 2535 | S4215 | Ribostamycin Sulfate | h5 | L1300-33 |
| 2536 | S4234 | Capreomycin Sulfate | a6 | L1300-33 |
| 2537 | S4236 | Proflavine Hemisulfate | b6 | L1300-33 |
| 2538 | S4245 | Sodium ascorbate | c6 | L1300-33 |
| 2539 | S4254 | Apramycin Sulfate | d6 | L1300-33 |
| 2540 | S4271 | Isepamicin Sulphate | e6 | L1300-33 |
| 2541 | S4287 | Micafungin Sodium | f6 | L1300-33 |
| 2542 | S4298 | Amifostine | g6 | L1300-33 |
| 2543 | S4311 | Calcium Gluceptate | h6 | L1300-33 |
| 2544 | S4314 | Ceftazidime Pentahydrate | a7 | L1300-33 |
| 2545 | S4328 | Guanethidine Sulfate | b7 | L1300-33 |
| 2546 | S4360 | Tolmetin Sodium | c7 | L1300-33 |
| 2547 | S4392 | Potassium Canrenoate | d7 | L1300-33 |
| 2548 | S4430 | Hydroxychloroquine Sulfate | e7 | L1300-33 |
| 2549 | S4523 | Dihydrostreptomycin sulfate | f7 | L1300-33 |
| 2550 | S4540 | Sisomicin sulfate | g7 | L1300-33 |
| 2551 | S4567 | Eprodisate disodium | h7 | L1300-33 |
| 2552 | S4575 | Pralidoxime chloride | a8 | L1300-33 |
| 2553 | S4582 | Eflornithine hydrochloride hydrate | b8 | L1300-33 |
| 2554 | S4592 | Captisol (SBE-β-CD) | c8 | L1300-33 |
| 2555 | S4606 | Glutathione | d8 | L1300-33 |
| 2556 | S4653 | L-Ornithine | e8 | L1300-33 |
| 2557 | S4671 | Cefradine | f8 | L1300-33 |
| 2558 | S4684 | Sildenafil | g8 | L1300-33 |
| 2559 | S4703 | Choline bitartrate | h8 | L1300-33 |
| 2560 | S4704 | D-(+)-Cellobiose | a9 | L1300-33 |
| 2561 | S4721 | L-Glutamic acid monosodium salt | b9 | L1300-33 |
| 2562 | S4740 | Sodium ferulate | c9 | L1300-33 |
| 2563 | S4741 | Danshensu | d9 | L1300-33 |
| 2564 | S4770 | Creatine phosphate disodium salt | e9 | L1300-33 |
| 2565 | S4810 | Glycylglycine | f9 | L1300-33 |
| 2566 | S4811 | VitaMin U | g9 | L1300-33 |
| 2567 | S4821 | Glycine | h9 | L1300-33 |
| 2568 | S4827 | Manganese chloride | a10 | L1300-33 |
| 2569 | S4828 | Sodium carbonate | b10 | L1300-33 |
| 2570 | S4950 | L(+)-Asparagine monohydrate | c10 | L1300-33 |
| 2571 | S4951 | L-Threonine | d10 | L1300-33 |
| 2572 | S4993 | Timonacic | e10 | L1300-33 |
| 2573 | S5048 | Fosfomycin Disodium | g10 | L1300-33 |
| 2574 | S5051 | Pipemidic acid | h10 | L1300-33 |
| 2575 | S5097 | Methotrexate disodium | a11 | L1300-33 |
| 2576 | S5131 | Homotaurine | b11 | L1300-33 |
| 2577 | S5136 | Calcium folinate | c11 | L1300-33 |
| 2578 | S5220 | D-Pantethine | d11 | L1300-33 |
| 2579 | S5252 | Ozagrel sodium | e11 | L1300-33 |
| 2580 | S5267 | Nylidrin Hydrochloride | f11 | L1300-33 |
| 2581 | S5280 | Dimemorfan phosphate | g11 | L1300-33 |
| 2582 | S5296 | Cephradine monohydrate | h11 | L1300-33 |
| 2583 | S5384 | UTP, Trisodium Salt | a2 | L1300-34 |
| 2584 | S5412 | Loxoprofen Sodium | b2 | L1300-34 |
| 2585 | S5433 | Sodium succinate | c2 | L1300-34 |
| 2586 | S5496 | Guanethidine Monosulfate | d2 | L1300-34 |
| 2587 | S5524 | Spermine Tetrahydrochloride | e2 | L1300-34 |
| 2588 | S5526 | β-Alanine | f2 | L1300-34 |
| 2589 | S5533 | Tobramycin sulfate | g2 | L1300-34 |
| 2590 | S5545 | DL-Serine | h2 | L1300-34 |
| 2591 | S5549 | DL-Arginine | a3 | L1300-34 |
| 2592 | S5571 | L-Asparagine | b3 | L1300-34 |
| 2593 | S5588 | Creatine | c3 | L1300-34 |
| 2594 | S5628 | L-Valine | e3 | L1300-34 |
| 2595 | S5629 | L-Proline | f3 | L1300-34 |
| 2596 | S5630 | L-lysine | g3 | L1300-34 |
| 2597 | S5631 | L-Alanine | h3 | L1300-34 |
| 2598 | S5632 | L-aspartic Acid | a4 | L1300-34 |
| 2599 | S5633 | L-methionine | b4 | L1300-34 |
| 2600 | S5634 | L-arginine | c4 | L1300-34 |
| 2601 | S5635 | L-cysteine | d4 | L1300-34 |
| 2602 | S5660 | Clodronate disodium tetrahydrate | e4 | L1300-34 |
| 2603 | S5674 | Lodoxamide Tromethamine | f4 | L1300-34 |
| 2604 | S5706 | Edetate Trisodium | g4 | L1300-34 |
| 2605 | S5721 | edetate calcium disodium | h4 | L1300-34 |
| 2606 | S5746 | pentetate calcium trisodium hydrate | a5 | L1300-34 |
| 2607 | S5820 | L-Hydroxyproline | b5 | L1300-34 |
| 2608 | S5866 | Gastrodenol | c5 | L1300-34 |
| 2609 | S5867 | Bendazac L-lysine | d5 | L1300-34 |
| 2610 | S6023 | Creatine monohydrate | e5 | L1300-34 |
| 2611 | S6201 | Malachite green | f5 | L1300-34 |
| 2612 | S6224 | (R)-Serine | g5 | L1300-34 |
| 2613 | S6266 | (S)-Glutamic acid | h5 | L1300-34 |
| 2614 | S6315 | Sodium phytate hydrate | a6 | L1300-34 |
| 2615 | S7377 | Aprotinin | b6 | L1300-34 |
| 2616 | S7548 | Rilmenidine Phosphate | c6 | L1300-34 |
| 2617 | S7785 | Pemetrexed Disodium Hydrate | d6 | L1300-34 |
| 2618 | S9344 | DL-Methionine | e6 | L1300-34 |
| 2619 | S9353 | L-serine | f6 | L1300-34 |
| 2620 | S9367 | Adenosine disodium triphosphate | g6 | L1300-34 |
| 2621 | S9471 | Gadoversetamide | a7 | L1300-34 |
| 2622 | P1004 | Bivalirudin Trifluoroacetate | b7 | L1300-34 |
| 2623 | P1011 | Eptifibatide Acetate | c7 | L1300-34 |
| 2624 | P1013 | Leuprorelin Acetate | d7 | L1300-34 |
| 2625 | P1015 | Lypressin Acetate | e7 | L1300-34 |
| 2626 | P1017 | Octreotide Acetate | f7 | L1300-34 |
| 2627 | P1023 | Alarelin Acetate | g7 | L1300-34 |
| 2628 | P1025 | Atosiban Acetate | h7 | L1300-34 |
| 2629 | P1026 | Gonadorelin Acetate | a8 | L1300-34 |
| 2630 | P1029 | Oxytocin (Syntocinon) | b8 | L1300-34 |
| 2631 | P1030 | Salmon Calcitonin Acetate | c8 | L1300-34 |
| 2632 | P1034 | Terlipressin Acetate | d8 | L1300-34 |
| 2633 | P1049 | GHRP-2 | e8 | L1300-34 |
| 2634 | P1061 | Nafarelin Acetate | f8 | L1300-34 |
| 2635 | S1671 | （6-）ε-Aminocaproic acid | g8 | L1300-34 |
| 2636 | P1084 | Desmopressin Acetate | h8 | L1300-34 |
| 2637 | P1085 | Angiotensin II human Acetate | a9 | L1300-34 |
| 2638 | P1087 | Carperitide Acetate | b9 | L1300-34 |
| 2639 | S5865 | Hyaluronic acid | c9 | L1300-34 |
| 2640 | S3076 | Foscarnet Sodium | d9 | L1300-34 |
| 2641 | S4519 | Citric acid trilithium salt tetrahydrate | e9 | L1300-34 |
| 2642 | P1039 | Somatostatin Acetate | f9 | L1300-34 |
| 2643 | P1089 | Goserelin Acetate | g9 | L1300-34 |
| 2644 | S6445 | Chlorophyllin (sodium copper salt) | h9 | L1300-34 |
| 2645 | S6460 | Sugammadex (sodium) | a10 | L1300-34 |
| 2646 | S6488 | Gadoxetate sodium | b10 | L1300-34 |
| 2647 | S4986 | Latamoxef sodium | c10 | L1300-34 |
| 2648 | S2441 | Estramustine phosphate sodium | d10 | L1300-34 |
| 2649 | S3948 | Spermine | e10 | L1300-34 |
| 2650 | S2435 | Sodium stibogluconate | f10 | L1300-34 |
| 2651 | S6831 | HEPES | g10 | L1300-34 |
| 2652 | S4933 | Lithium carbonate | h10 | L1300-34 |
| 2653 | S4972 | Choline Glycerophosphate | a11 | L1300-34 |
| 2654 | S6592 | Diquafosol Tetrasodium | b11 | L1300-34 |
| 2655 | S3203 | D-Alanine | c11 | L1300-34 |
| 2656 | S1023 | Erlotinib HCl (OSI-744) | a2 | L1300-35 |
| 2657 | S1046 | Vandetanib (ZD6474) | b2 | L1300-35 |
| 2658 | S1211 | Imiquimod | c2 | L1300-35 |
| 2659 | S1288 | Camptothecin | d2 | L1300-35 |
| 2660 | S1353 | Ketoconazole | e2 | L1300-35 |
| 2661 | S1371 | Cefoselis Sulfate | f2 | L1300-35 |
| 2662 | S1424 | Prazosin HCl | g2 | L1300-35 |
| 2663 | S1464 | Marbofloxacin | h2 | L1300-35 |
| 2664 | S1507 | Irbesartan | a3 | L1300-35 |
| 2665 | S1509 | Norfloxacin | b3 | L1300-35 |
| 2666 | S1615 | Risperidone | c3 | L1300-35 |
| 2667 | S1617 | Sulfapyridine | d3 | L1300-35 |
| 2668 | S1642 | Methyldopa | e3 | L1300-35 |
| 2669 | S1698 | Torsemide | f3 | L1300-35 |
| 2670 | S1707 | Eplerenone | g3 | L1300-35 |
| 2671 | S1724 | Paliperidone | h3 | L1300-35 |
| 2672 | S1767 | Beta Carotene | a4 | L1300-35 |
| 2673 | S1837 | Flubendazole | b4 | L1300-35 |
| 2674 | S1851 | Oxibendazole | c4 | L1300-35 |
| 2675 | S1929 | Irsogladine | d4 | L1300-35 |
| 2676 | S1977 | Sarafloxacin HCl | e4 | L1300-35 |
| 2677 | S1986 | Meclizine 2HCl | f4 | L1300-35 |
| 2678 | S2035 | Epalrestat | g4 | L1300-35 |
| 2679 | S2047 | Lornoxicam | h4 | L1300-35 |
| 2680 | S2110 | Vinpocetine | a5 | L1300-35 |
| 2681 | S2150 | Neratinib (HKI-272) | b5 | L1300-35 |
| 2682 | S2152 | Sitafloxacin Hydrate | c5 | L1300-35 |
| 2683 | S2206 | R788 (Fostamatinib) Disodium | d5 | L1300-35 |
| 2684 | S2232 | Ketanserin | e5 | L1300-35 |
| 2685 | S2251 | (-)-Huperzine A (HupA) | f5 | L1300-35 |
| 2686 | S2292 | Diosmin | g5 | L1300-35 |
| 2687 | S2424 | Hypoxanthine | h5 | L1300-35 |
| 2688 | S2476 | Itraconazole | a6 | L1300-35 |
| 2689 | S2530 | 7-Aminocephalosporanic acid | b6 | L1300-35 |
| 2690 | S2582 | Trazodone HCl | c6 | L1300-35 |
| 2691 | S2597 | Oseltamivir Phosphate | d6 | L1300-35 |
| 2692 | S2920 | Mozavaptan | e6 | L1300-35 |
| 2693 | S3059 | Enrofloxacin | f6 | L1300-35 |
| 2694 | S3064 | Ambroxol HCl | g6 | L1300-35 |
| 2695 | S3721 | Bilastine | h6 | L1300-35 |
| 2696 | S3770 | Sodium Aescinate | a7 | L1300-35 |
| 2697 | S3801 | Sodium Houttuyfonate | b7 | L1300-35 |
| 2698 | S3821 | Nuciferine | c7 | L1300-35 |
| 2699 | S3891 | Vincamine | d7 | L1300-35 |
| 2700 | S4126 | Retinyl (Vitamin A) Palmitate | e7 | L1300-35 |
| 2701 | S4142 | Clopidol | f7 | L1300-35 |
| 2702 | S4156 | Chlortetracycline HCl | g7 | L1300-35 |
| 2703 | S4218 | Amoxapine | h7 | L1300-35 |
| 2704 | S4262 | Ebastine | a8 | L1300-35 |
| 2705 | S4605 | Folic acid | b8 | L1300-35 |
| 2706 | S4654 | Netupitant | c8 | L1300-35 |
| 2707 | S4930 | Cetilistat | e8 | L1300-35 |
| 2708 | S5018 | Mebhydrolin napadisylate | f8 | L1300-35 |
| 2709 | S5035 | 6-Aminopenicillanic acid | g8 | L1300-35 |
| 2710 | S5232 | Alectinib hydrochloride | a9 | L1300-35 |
| 2711 | S5499 | Amantadine | b9 | L1300-35 |
| 2712 | S5601 | Balofloxacin Dihydrate | c9 | L1300-35 |
| 2713 | S5677 | Xanthopterin Hydrate | d9 | L1300-35 |
| 2714 | S5908 | Garenoxacin | e9 | L1300-35 |
| 2715 | S3636 | Cefadroxil hydrate | f9 | L1300-35 |
| 2716 | S3139 | Methenamine | g9 | L1300-35 |
| 2717 | S4033 | Sennoside A | h9 | L1300-35 |
| 2718 | S5712 | Zofenopril calcium | a10 | L1300-35 |
| 2719 | S1463 | Ofloxacin | b10 | L1300-35 |
| 2720 | S3041 | Droxidopa | c10 | L1300-35 |
| 2721 | S4642 | Dolutegravir Sodium | d10 | L1300-35 |
| 2722 | S6632 | Mirogabalin | e10 | L1300-35 |
| 2723 | S8229 | Brigatinib (AP26113) | f10 | L1300-35 |
| 2724 | S5098 | Gefitinib hydrochloride | g10 | L1300-35 |
| 2725 | S5031 | Sulfaquinoxaline sodium | h10 | L1300-35 |
| 2726 | S1502 | Cephalexin | a11 | L1300-35 |
| 2727 | S2788 | Capmatinib (INCB28060) | b11 | L1300-35 |
| 2728 | S7128 | Tazemetostat (EPZ-6438) | c11 | L1300-35 |
| 2729 | S7756 | Indoximod (NLG-8189） | d11 | L1300-35 |
| 2730 | S1224 | Oxaliplatin | a2 | L1300-36 |
| 2731 | S1346 | Heparin sodium | b2 | L1300-36 |
| 2732 | S1428 | Risedronate Sodium | c2 | L1300-36 |
| 2733 | S1911 | Disodium Cromoglycate | d2 | L1300-36 |
| 2734 | S1982 | Adenine sulfate | e2 | L1300-36 |
| 2735 | S2106 | Azasetron HCl | f2 | L1300-36 |
| 2736 | S2562 | Hydralazine HCl | g2 | L1300-36 |
| 2737 | S3055 | Besifloxacin HCl | h2 | L1300-36 |
| 2738 | S3749 | Calcium gluconate | a3 | L1300-36 |
| 2739 | S4067 | Deferiprone | b3 | L1300-36 |
| 2740 | S4153 | D-Phenylalanine | c3 | L1300-36 |
| 2741 | S4226 | Minocycline HCl | d3 | L1300-36 |
| 2742 | S4694 | Alosetron Hydrochloride | e3 | L1300-36 |
| 2743 | S5355 | Piperaquine phosphate | f3 | L1300-36 |
| 2744 | S5695 | Icatibant Acetate | g3 | L1300-36 |
| 2745 | S8030 | Plerixafor (AMD3100) | h3 | L1300-36 |
| 2746 | S5650 | Sodium Hyaluronate | a4 | L1300-36 |
| 2747 | S5036 | Nisin | b4 | L1300-36 |

Formulation:

In 10 mM DMSO: Plate layout L1300-01~31;

In 2 mM DMSO: Plate layout L1300-35;

In 10 mM Water: Plate layout L1300-32~34;

In 2 mM Water: Plate layout L1300-36.

**Table S2. Mass spectrometry of CeeNU-interacting protein.**

| Master | Accession | Gene Symbol | Coverage [%] | # Peptides | # Unique Peptides | MW [kDa] |
| --- | --- | --- | --- | --- | --- | --- |
| Master Protein | Q96P20 | NLRP3 | 29 | 29 | 29 | 118.1 |
| Master Protein | P09874 | PARP1 | 20 | 20 | 20 | 113 |
| Master Protein | P13645 | KRT10 | 20 | 12 | 12 | 58.8 |
| Master Protein | P04264 | KRT1 | 22 | 13 | 11 | 66 |
| Master Protein | P35527 | KRT9 | 21 | 11 | 11 | 62 |
| Master Protein | Q08211 | DHX9 | 10 | 11 | 11 | 140.9 |
| Master Protein | P35573 | AGL | 8 | 11 | 11 | 174.7 |
| Master Protein | P78347 | GTF2I | 10 | 10 | 10 | 112.3 |
| Master Protein | P35908 | KRT2 | 17 | 11 | 8 | 65.4 |
| Master Protein | P13647 | KRT5 | 16 | 10 | 7 | 62.3 |
| Master Protein | P43243 | MATR3 | 12 | 7 | 7 | 94.6 |
| Master Protein | P02533 | KRT14 | 13 | 7 | 7 | 51.5 |
| Master Protein | O14654 | IRS4 | 7 | 6 | 6 | 133.7 |
| Master Protein | Q14683 | SMC1A | 5 | 6 | 6 | 143.1 |
| Master Protein | P55060 | CSE1L | 6 | 6 | 6 | 110.3 |
| Master Protein | P02538 | KRT6A | 17 | 9 | 5 | 60 |
| Master Protein | O75533 | SF3B1 | 4 | 5 | 5 | 145.7 |
| Master Protein | P78527 | PRKDC | 1 | 5 | 5 | 468.8 |
| Master Protein | P60709 | ACTB | 14 | 4 | 4 | 41.7 |
| Master Protein | P42285 | MTREX | 4 | 4 | 4 | 117.7 |
| Master Protein | P08238 | HSP90AB1 | 9 | 5 | 4 | 83.2 |
| Master Protein | Q16643 | DBN1 | 10 | 4 | 4 | 71.4 |
| Master Protein | Q12906 | ILF3 | 5 | 4 | 4 | 95.3 |
| Master Protein | P07814 | EPRS1 | 3 | 4 | 4 | 170.5 |
| Master Protein | Q16531 | DDB1 | 4 | 4 | 4 | 126.9 |
| Master Protein | P19338 | NCL | 6 | 4 | 4 | 76.6 |
| Master Protein | Q92598 | HSPH1 | 6 | 4 | 4 | 96.8 |
| Master Protein | P02768 | ALB | 4 | 3 | 3 | 69.3 |
| Master Protein | Q9H307 | PNN | 5 | 3 | 3 | 81.6 |
| Master Protein | P53396 | ACLY | 3 | 3 | 3 | 120.8 |
| Master Protein | P46379 | BAG6 | 3 | 3 | 3 | 119.3 |
| Master Protein | Q00839 | HNRNPU | 4 | 3 | 3 | 90.5 |
| Master Protein | Q9P035 | HACD3 | 9 | 3 | 3 | 43.1 |
| Master Protein | O14980 | XPO1 | 3 | 3 | 3 | 123.3 |
| Master Protein | P35579 | MYH9 | 1 | 3 | 3 | 226.4 |
| Master Protein | Q05639 | EEF1A2 | 6 | 3 | 3 | 50.4 |
| Master Protein | P42704 | LRPPRC | 2 | 3 | 3 | 157.8 |
| Master Protein | P49588 | AARS1 | 3 | 3 | 3 | 106.7 |
| Master Protein | P12814 | ACTN1 | 8 | 6 | 2 | 103 |
| Master Protein | O43707 | ACTN4 | 8 | 6 | 2 | 104.8 |
| Master Protein | P14625 | HSP90B1 | 4 | 3 | 2 | 92.4 |
| Master Protein | Q9Y2W1 | THRAP3 | 2 | 2 | 2 | 108.6 |
| Master Protein | P62987 | UBA52 | 23 | 2 | 2 | 14.7 |
| Master Protein | P68871 | HBB | 17 | 2 | 2 | 16 |
| Master Protein | Q9Y5B9 | SUPT16H | 2 | 2 | 2 | 119.8 |
| Master Protein | Q92900 | UPF1 | 3 | 2 | 2 | 124.3 |
| Master Protein | Q15459 | SF3A1 | 4 | 2 | 2 | 88.8 |
| Master Protein | Q15393 | SF3B3 | 3 | 2 | 2 | 135.5 |
| Master Protein | Q15029 | EFTUD2 | 2 | 2 | 2 | 109.4 |
| Master Protein | Q96SB3 | PPP1R9B | 3 | 2 | 2 | 89.3 |
| Master Protein | P30876 | POLR2B | 2 | 2 | 2 | 133.8 |
| Master Protein | P23246 | SFPQ | 3 | 2 | 2 | 76.1 |
| Master Protein | Q15424 | SAFB | 2 | 2 | 2 | 102.6 |
| Master Protein | Q86VP6 | CAND1 | 2 | 2 | 2 | 136.3 |
| Master Protein | Q86UP2 | KTN1 | 2 | 2 | 2 | 156.2 |
| Master Protein | Q9Y4C2 | TCAF1 | 2 | 2 | 2 | 102.1 |
| Master Protein | Q14203 | DCTN1 | 2 | 2 | 2 | 141.6 |
| Master Protein | Q9NR30 | DDX21 | 3 | 2 | 2 | 87.3 |
| Master Protein | P11586 | MTHFD1 | 2 | 2 | 2 | 101.5 |
| Master Protein | P28340 | POLD1 | 2 | 2 | 2 | 123.6 |
| Master Protein | Q6PKG0 | LARP1 | 2 | 2 | 2 | 123.4 |
| Master Protein | Q7L2E3 | DHX30 | 2 | 2 | 2 | 133.9 |
| Master Protein | O94832 | MYO1D | 2 | 2 | 2 | 116.1 |
| Master Protein | Q99460 | PSMD1 | 2 | 2 | 2 | 105.8 |
| Master Protein | Q13435 | SF3B2 | 2 | 2 | 2 | 100.2 |
| Master Protein | P52948 | NUP98 | 1 | 2 | 2 | 197.5 |
| Master Protein | P46087 | NOP2 | 3 | 2 | 2 | 89.2 |
| Master Protein | Q13200 | PSMD2 | 2 | 2 | 2 | 100.1 |
| Master Protein | Q9UQE7 | SMC3 | 2 | 2 | 2 | 141.5 |
| Master Protein | P11388 | TOP2A | 2 | 2 | 2 | 174.3 |
| Master Protein | O95347 | SMC2 | 2 | 2 | 2 | 135.6 |
| Master Protein | Q6P2Q9 | PRPF8 | 1 | 2 | 2 | 273.4 |
| Master Protein | Q93100 | PHKB | 2 | 2 | 2 | 124.8 |
| Master Protein | P49327 | FASN | 1 | 2 | 2 | 273.3 |
| Master Protein | P49916 | LIG3 | 1 | 1 | 1 | 112.8 |
| Master Protein | Q86YZ3 | HRNR | 3 | 1 | 1 | 282.2 |
| Master Protein | Q14671 | PUM1 | 1 | 1 | 1 | 126.4 |
| Master Protein | Q00610 | CLTC | 1 | 1 | 1 | 191.5 |
| Master Protein | Q9NYF8 | BCLAF1 | 1 | 1 | 1 | 106.1 |
| Master Protein | O75390 | CS | 2 | 1 | 1 | 51.7 |
| Master Protein | Q9H0A0 | NAT10 | 1 | 1 | 1 | 115.7 |
| Master Protein | Q8TEX9 | IPO4 | 1 | 1 | 1 | 118.6 |
| Master Protein | P41252 | IARS1 | 1 | 1 | 1 | 144.4 |
| Master Protein | Q14527 | HLTF | 1 | 1 | 1 | 113.9 |
| Master Protein | Q9Y2H2 | INPP5F | 1 | 1 | 1 | 128.3 |
| Master Protein | Q14444 | CAPRIN1 | 2 | 1 | 1 | 78.3 |
| Master Protein | Q9UHB6 | LIMA1 | 1 | 1 | 1 | 85.2 |
| Master Protein | P36578 | RPL4 | 3 | 1 | 1 | 47.7 |
| Master Protein | A0A087WW87 | IGKV2-40 | 11 | 1 | 1 | 13.3 |
| Master Protein | P10809 | HSPD1 | 1 | 1 | 1 | 61 |
| Master Protein | Q13885 | TUBB2A | 2 | 1 | 1 | 49.9 |
| Master Protein | P49756 | RBM25 | 1 | 1 | 1 | 100.1 |
| Master Protein | P07205 | PGK2 | 4 | 1 | 1 | 44.8 |
| Master Protein | P14618 | PKM | 2 | 1 | 1 | 57.9 |
| Master Protein | P33991 | MCM4 | 1 | 1 | 1 | 96.5 |
| Master Protein | P53621 | COPA | 1 | 1 | 1 | 138.3 |
| Master Protein | O15042 | U2SURP | 1 | 1 | 1 | 118.2 |
| Master Protein | Q8N1N4 | KRT78 | 2 | 1 | 1 | 56.8 |
| Master Protein | Q96N67 | DOCK7 | 1 | 1 | 1 | 242.4 |
| Master Protein | P57088 | TMEM33 | 4 | 1 | 1 | 28 |
| Master Protein | P27708 | CAD | 0 | 1 | 1 | 242.8 |
| Master Protein | Q15269 | PWP2 | 1 | 1 | 1 | 102.4 |
| Master Protein | O00410 | IPO5 | 1 | 1 | 1 | 123.6 |
| Master Protein | P16402 | H1-3 | 4 | 1 | 1 | 22.3 |
| Master Protein | Q14978 | NOLC1 | 1 | 1 | 1 | 73.6 |
| Master Protein | Q9UNX4 | WDR3 | 1 | 1 | 1 | 106 |
| Master Protein | P11387 | TOP1 | 1 | 1 | 1 | 90.7 |
| Master Protein | Q9Y3T9 | NOC2L | 1 | 1 | 1 | 84.9 |
| Master Protein | Q02878 | RPL6 | 3 | 1 | 1 | 32.7 |
| Master Protein | Q9BTW9 | TBCD | 1 | 1 | 1 | 132.5 |
| Master Protein | P04843 | RPN1 | 2 | 1 | 1 | 68.5 |
| Master Protein | B5ME19 | EIF3CL | 1 | 1 | 1 | 105.4 |
| Master Protein | O75400 | PRPF40A | 1 | 1 | 1 | 108.7 |
| Master Protein | P02765 | AHSG | 2 | 1 | 1 | 39.3 |
| Master Protein | O95782 | AP2A1 | 1 | 1 | 1 | 107.5 |
| Master Protein | O00159 | MYO1C | 1 | 1 | 1 | 121.6 |
| Master Protein | O15027 | SEC16A | 0 | 1 | 1 | 251.7 |
| Master Protein | Q86VM9 | ZC3H18 | 1 | 1 | 1 | 106.3 |
| Master Protein | Q8N163 | CCAR2 | 1 | 1 | 1 | 102.8 |
| Master Protein | P62917 | RPL8 | 4 | 1 | 1 | 28 |
| Master Protein | P0DJD0 | RGPD1 | 0 | 1 | 1 | 196.5 |
| Master Protein | Q86XI2 | NCAPG2 | 1 | 1 | 1 | 130.9 |
| Master Protein | A6NMY6 | ANXA2P2 | 3 | 1 | 1 | 38.6 |
| Master Protein | Q9BPX3 | NCAPG | 1 | 1 | 1 | 114.3 |
| Master Protein | O94906 | PRPF6 | 1 | 1 | 1 | 106.9 |
| Master Protein | P62805 | H4C1 | 10 | 1 | 1 | 11.4 |
| Master Protein | P22314 | UBA1 | 1 | 1 | 1 | 117.8 |
| Master Protein | Q5JTH9 | RRP12 | 1 | 1 | 1 | 143.6 |
| Master Protein | Q5T8P6 | RBM26 | 1 | 1 | 1 | 113.5 |
| Master Protein | Q9NTJ3 | SMC4 | 1 | 1 | 1 | 147.1 |
| Master Protein | Q9P2R3 | ANKFY1 | 1 | 1 | 1 | 128.3 |
| Master Protein | Q14137 | BOP1 | 1 | 1 | 1 | 83.6 |
| Master Protein | P0DPH8 | TUBA3D | 2 | 1 | 1 | 49.9 |
| Master Protein | Q14146 | URB2 | 1 | 1 | 1 | 170.4 |
| Master Protein | P05556 | ITGB1 | 1 | 1 | 1 | 88.4 |
| Master Protein | Q16777 | H2AC20 | 5 | 1 | 1 | 14 |
| Master Protein | Q13428 | TCOF1 | 1 | 1 | 1 | 152 |
| Master Protein | Q9NNW5 | WDR6 | 1 | 1 | 1 | 121.6 |
| Master Protein | B9A064 | IGLL5 | 4 | 1 | 1 | 23 |
| Master Protein | Q04637 | EIF4G1 | 1 | 1 | 1 | 175.4 |
| Master Protein | Q92841 | DDX17 | 2 | 1 | 1 | 80.2 |
| Master Protein | Q8NI08 | NCOA7 | 5 | 1 | 1 | 106.1 |

**Table S3. Oligonucleotide primers used in reverse transcription real-time quantitative PCR**

| Primer Names | Primer sequence |
| --- | --- |
| *GAPDH*-F | 5’-AGGTCGGTGTGAACGGATTTG-3’ |
| *GAPDH*-R | 5’-GGGGTCGTTGATGGCAACA-3’ |
| *NLRP3*-F | 5’-ATCAACAGGCGAGACCTCTG-3’ |
| *NLRP3*-R | 5’-GTCCTCCTGGCATACCATAGA-3’ |
| *IL-1β*-F | 5’-AAATACCTGTGGCCTTGGGC-3’ |
| *IL-1β*-R | 5’-CTTGGGATCCACACTCTCCAG-3’ |
| *IL-6*-F | 5’-CTGCAAGAGACTTCCATCCAG-3’ |
| *IL-6*-R | 5’-AGTGGTATAGACAGGTCTGTTGG-3’ |
| *IL-17-F* | 5’-GTCCAGGGAGAGCTTCATCTG-3’ |
| *IL-17*-R | 5’-CTTGGCCTCAGTGTTTGGAC-3’ |
| *TNF-α*-F | 5’-CTGAACTTCGGGGTGATCGG-3’ |
| *TNF-α*-R | 5’-GGCTTGTCACTCGAATTTTGAGA-3’ |
| *IFN-γ-*F | 5’-CTCATGGCTGTTTCTGGCTG-3’ |
| *IFN-γ*-R | 5’-CCTTTTGCCAGTTCCTCCAG-3’ |
